# Non-pharmacological Treatment for Elderly Individuals With Insomnia: A Systematic Review and Network Meta-Analysis

**DOI:** 10.3389/fpsyt.2020.608896

**Published:** 2021-01-28

**Authors:** Chan-Young Kwon, Boram Lee, Moon Joo Cheong, Tae-Hun Kim, Bo-Hyoung Jang, Sun Yong Chung, Jong Woo Kim

**Affiliations:** ^1^Department of Oriental Neuropsychiatry, Dong-eui University College of Korean Medicine, Busan, South Korea; ^2^Clinical Medicine Division, Korea Institute of Oriental Medicine, Daejeon, South Korea; ^3^Education Graduate of Wonkwang University, Iksan-si, South Korea; ^4^Korean Medicine Clinical Trial Center, Korean Medicine Hospital, Kyung Hee University, Seoul, South Korea; ^5^Department of Preventive Medicine, College of Korean Medicine, Kyung Hee University, Seoul, South Korea; ^6^Department of Neuropsychiatry, Kyung Hee University Korean Medicine Hospital at Gangdong, Seoul, South Korea

**Keywords:** aged, systematic review, network meta-analysis, elderly, insomnia

## Abstract

**Background:** Insomnia causes a huge socioeconomic burden among the elderly, and is not simply a health problem. This study aimed to determine the comparative advantage of the effectiveness and acceptability of non-pharmacological interventions available for elderly individuals with insomnia.

**Methods:** Comprehensive searches in 13 medical databases were performed to find relevant randomized controlled trials (RCTs) up to August 2019. Two independent reviewers performed study selection, data extraction, and quality assessment of included RCTs using the Cochrane Collaboration's risk of bias. A network meta-analysis within the frequentist model was performed by combining direct and indirect evidence from all available RCTs. The primary outcomes were effectiveness as measured by the Pittsburgh Sleep Quality Index (PSQI) total score and acceptability by the incidence of all-cause drop-out.

**Results:** Twenty-eight RCTs involving 2,391 participants were included. Compared to wait-list, acupuncture (standardized mean difference −4.37, 95% confidence interval −8.53 to −0.12), acupuncture combined with benzodiazepines (−5.20, −9.82 to −0.57), behavioral therapy (−10.44, −17.31 to −3.58), benzodiazepines (−4.28, −8.45 to −0.11), benzodiazepines combined with cognitive behavioral therapy (CBT) (−7.18, −12.17 to −2.19), and CBT (−4.93, −8.63 to −1.22) showed significant superiority in their effectiveness. No significant comparative superiority or inferiority was found in terms of acceptability.

**Conclusions:** In terms of effectiveness as indicated by the PSQI total score, compared to wait-list, superior benefits were observed for acupuncture, acupuncture combined with benzodiazepines, behavioral treatment, benzodiazepines, benzodiazepines combined with CBT, and CBT. Importantly, combined treatments, including benzodiazepines combined with CBT or with acupuncture, were generally superior to other monotherapies. In terms of acceptability, there was not enough data to draw conclusions. However, most RCTs included had methodological problems related to the lack of blinding procedure, suggesting a risk of effect size overestimation.

**Registration:** CRD42019145518.

## Introduction

Insomnia is a common mental health problem that can be defined as “*a complaint of trouble initiating or maintaining sleep which is associated with daytime consequences and is not attributable to environmental circumstances or inadequate opportunity to sleep*,” according to the American Academy of Sleep Medicine (1). The American Insomnia Survey, an epidemiological survey, showed that insomnia in the general population reached 22.1% based on the *Diagnostic and Statistical Manual of Mental Disorders* (*DSM*)-IV-TR (2). The prevalence of insomnia increases with age, and its prevalence in the elderly is known to be around 20–55% (1, 3, 4). Chronic insomnia constitutes a vicious cycle that makes the disease more susceptible to fixation. Risk factors for insomnia include poor mental health, poor sleep, and obesity (5). Several non-pharmacological approaches and pharmacotherapy can be applied to treat insomnia. According to international guidelines, cognitive-behavioral therapy for insomnia (CBT-I) is considered a standard treatment (1, 6, 7). If CBT-I alone is not effective, pharmacotherapy or other behavioral interventions may be considered (8). However, the administration of drugs—especially benzodiazepine hypnotics—should be carefully performed considering the benefits and risks (1, 6, 7).

In the elderly, insomnia has important characteristics that are quite different from insomnia in the general population. There are several reasons why insomnia issues are important to the elderly. First, in elderly people, the vulnerability to insomnia increases with aging-related changes (9), and some medications that the elderly are regularly taking can cause insomnia. Second, there is growing evidence that insomnia is associated with cognitive impairments (10), dementia (11), depression (12), cardio-cerebral vascular events (13, 14), other health conditions (15), and even mortality (16), which are often associated with the elderly. Therefore, insomnia in the elderly is not only a health-related problem but also carries a socioeconomic burden. Third, among current available existing treatments for insomnia, pharmacotherapies are sometimes associated with serious adverse reactions in the elderly. Although benzodiazepines and other sedative-hypnotic drugs can be used to treat problems related to anxiety or insomnia, their use is not recommended or limited because they are associated with serious side effects such as increased risks of falls and hip fractures among the elderly (17–19). Furthermore, CBT-I is an effective treatment for the improvement of insomnia in the elderly (20); however, there is still a need for some treatment options that can complement or be alternated with this treatment because it is labor-intensive and usually takes generally 6 to 8 weeks. Therefore, it is important to find an effective, simple, and safe treatment method for insomnia in the elderly. This is particularly true for non-pharmacological methods. Moreover, although several non-pharmacological interventions, including CBT-I, have been discussed in the treatment of insomnia, understanding their comparative effectiveness and acceptability allows for optimal medical choice.

Although evidence-based clinical guidelines specifically addressing sleep disturbances in the elderly are lacking, the most recent evidence-based recommendation in 2009, developed by international experts on sleep disorders, include pharmacotherapies including benzodiazepines, non-benzodiazepines, and melatonin receptor agonists, as well as non-pharmacological treatments including CBT-I (21). Moreover, these guidelines also suggested non-pharmacological treatments, including complementary and integrative medicine (CIM) modalities such as acupuncture/acupressure, Tai Chi, and weight training (21). For the efficient distribution of medical resources and optimal medical choices, it is important to prioritize among the various approaches. In this regard, network meta-analysis (NMA) is a useful tool for developing clinical practice guidelines (CPGs) because it enables direct and indirect quantitative comparisons of different interventions and, above all, helps to prioritize these interventions (22). In other words, NMA allows both direct and indirect comparisons to compare the effectiveness, safety, and acceptability of three or more treatment options. This has led to NMA to be adopted as a new methodology by many international CPGs (23, 24).

The aim of this review was to compare individual non-pharmacological interventions in the treatment of insomnia in the elderly in terms of effectiveness, acceptability, and safety. We applied a systematic review and NMA methodology to generate a clinically useful evidence-based hierarchy of non-pharmacological interventions for insomnia in the elderly, according to their effectiveness, acceptability, and safety, using both NMA and classical pair-wise meta-analysis.

## Methods

The protocol of this NMA was registered in PROSPERO (registration number CRD42019145518). This review was reported per the Preferred Reporting Items for Systematic Reviews and Meta-Analyses (PRISMA) statement for reporting of systematic review incorporating NMA of health care interventions (Supplementary Table 1) (25).

### Search Strategy

Comprehensive searches were conducted in the following 13 electronic medical databases from their inception dates to August 5, 2019: six English-language databases [MEDLINE via PubMed, EMBASE via Elsevier, the Cochrane Central Register of Controlled Trials (CENTRAL), the Allied and Complementary Medicine Database (AMED) via EBSCO, the Cumulative Index to Nursing and Allied Health Literature (CINAHL) via EBSCO, and PsycARTICLES via ProQuest], five Korean-language databases [Oriental Medicine Advanced Searching Integrated System (OASIS), Koreanstudies Information Service System (KISS), Research Information Service System (RISS), Korean Medical Database (KMbase), and Korea Citation Index (KCI)], and two Chinese-language databases [China National Knowledge Infrastructure (CNKI) and Wanfang Data]. Manual searches of the reference lists of the relevant systematic reviews and included studies were also conducted to identify further eligible studies. Not only the literature published in journals, but also gray literature, such as theses and conference proceedings, were allowed. No restriction on language was imposed. The search strategies for each database are presented in Supplementary Tables 2–14.

### Eligible Criteria

#### Types of Studies

Only randomized controlled trials (RCTs) were included. Studies using inappropriate random sequence generation methods such as allocation by odd or even date of birth or admission day were excluded. To minimize sources of potential heterogeneity, we excluded cluster-randomized trials and cross-over trials.

#### Types of Participants

Studies on elderly people with a minimum age of 60 years, with the diagnosis of insomnia, using standardized diagnostic tools such as *DSM* (26), the International Statistical Classification of Diseases and Related Health Problems (ICD) (27), the International Classification of Sleep Disorders (ICSD) (28), and the Chinese Classification of Mental Disorders (CCMD) (29), were included. If the study did not present the participants' age criteria or age ranges and presented only the average age, they were excluded because their minimum age was not identified. There was no restriction on the severity of insomnia, sex, ethnicity, or race of patients. Studies were excluded if the patients have drug allergies or other serious medical conditions such as cancer, liver disease, or kidney disease.

#### Types of Interventions and Comparators

Studies comparing any two of the following non-pharmacological interventions proposed in the most recent international guideline for elderly individuals with insomnia (21) were included irrespective of the form (e.g., group or individual) and duration of treatment: cognitive behavioral therapy (CBT), behavioral treatment (BT) including multi-component behavioral treatments for insomnia, sleep hygiene only (including sleep education), sleep restriction only, stimulus control only, relaxation therapy (including meditation), exercise (including walking and weight training), Tai Chi (including qigong), and acupuncture (including acupressure). Placebo, no treatment, or active controls, including conventional medication, were allowed as control interventions. The inclusion for conventional medication was as follow according to the Cochrane NMA review protocol of pharmacological treatments for insomnia (30): antidepressants (amitriptyline, doxepin, mirtazapine, and trazodone), benzodiazepines (brotizolam, clonazepam, diazepam, estazolam, flunitrazepam, flurazepam, haloxazolam, loprazolam, lorazepam, lormetazepam, midazolam, nimetazepam, nitrazepam, quazepam, rilmazafone, temazepam, and triazolam), benzodiazepine-like agents (eszopiclone, zaleplon, zolpidem, and zopiclone), melatoninergic drugs (melatonin and ramelteon), and orexin receptor antagonists (suvorexant). For the combined treatment study, up to two combinations of defined interventions for the intervention group and control group (e.g., CBT-I plus walking, relaxation plus benzodiazepines) were allowed. In multi-arm trials, study groups assessing interventions other than those mentioned above were not eligible.

#### Types of Outcome Measures

##### Primary outcomes

(1) Sleep quality measured by validated assessment tools, such as the Pittsburgh Sleep Quality Index (PSQI) (31), the Insomnia Severity Index (ISI) (32), or the Leeds Sleep Evaluation Questionnaire (LSEQ) (33).

(2) Acceptability measured by drop-outs for any reason (as an indirect indicator of participants' adherence).

##### Secondary outcomes

(1) Drop-outs because of any adverse event (AE).

(2) Data from polysomnography including sleep onset latency (SOL), wake time after sleep onset (WASO), and total sleep time (TST).

(3) AEs measured by the Treatment Emergent Symptom Scale (TESS) (34) or the incidence of AEs.

#### Timing of Outcome Assessment

For the outcomes of sleep quality and polysomnography data, we considered the outcomes at 6-week post-treatment. If there was no 6-week post-treatment evaluation, the results at the closest time point were considered. However, the results of 6–8 weeks were given priority. That is, the 8-week post-treatment evaluation result was preferred over the 4-week post-treatment evaluation result.

### Study Selection and Data Extraction

Two researchers (C-YK, BL) independently conducted study selection and data extraction processes. Any disagreement about study selection and data extraction was resolved through discussion. The titles and abstracts of all searched studies were reviewed for relevance, and then the full texts of the eligible studies were evaluated for final inclusion. The data were extracted using a standardized data collection form (Excel 2007, Microsoft, Redmond, WA, USA). The extracted items included the first author's name; year of publication; country; sample size and the number of drop-outs; details about the participants, treatment intervention, control intervention, and comparisons; duration of the intervention; outcome measures; and AEs associated with interventions. If the data were insufficient or ambiguous, the corresponding authors of the included studies were contacted by e-mail to request additional information.

### Risk of Bias Assessment

Two researchers (C-YK, BL) independently assessed the methodological quality of the included RCTs, using the Cochrane risk of bias assessment tool (35), which includes the following items: random sequence generation, allocation concealment, blinding of participants, personnel and outcomes assessors, incomplete outcome data, selective outcome reporting, and other sources of bias. Each item was assessed as being of “low risk,” “high risk,” or “unclear risk” of bias. Moreover, the Jadad scale was used to supplement the methodological quality assessment (36). This scale is used to evaluate the appropriateness of the randomization, blinding, and the descriptions of withdrawals and dropouts, with a total score ranging from 0 (very poor) to 5 (rigorous) (36). Any discrepancies were resolved through their discussion. The potential baseline imbalance can cause a bias in the estimated effects of intervention in RCTs (35), which in turn can affect the similarity hypothesis in NMA. Therefore, in cases of other sources of bias, we assessed them as “low risk” when the statistical similarity on participant's mean age, insomnia period, insomnia severity, and so on at baseline between the groups, as described. The risk of bias figures were created using Review Manager Version 5.3 software (Cochrane, London, UK).

### Data Analysis

#### Pair-Wise Meta-Analysis (Conventional Meta-Analysis)

Pair-wise meta-analysis was performed on the primary and secondary outcomes for studies using the same types of intervention, comparison, and outcome measure. To perform the pair-wise meta-analysis, Review Manager Version 5.3 software (Cochrane, London, UK) was used. Continuous outcomes and dichotomous outcomes were pooled as the standardized mean difference (SMD) and odds ratio (OR), with 95% confidence intervals (CIs). By using both the chi-squared test and the I-squared statistic (*I*^2^), heterogeneity of effect measures between the studies was assessed. The value of *I*^2^ ≥ 50% was considered to be substantial, and the value of *I*^2^ ≥ 75% to be considerable heterogeneity (37). When the heterogeneity was considerable (*I*^2^ ≥ 75%), a random-effects model was used; otherwise, a fixed-effects model was used. Also, when there were fewer than five studies included in the meta-analysis, only a fixed-effects model was used (38, 39).

#### Network Meta-Analysis

The NMA within the frequentist model was performed by combining direct and indirect evidence from all available RCTs. Stata software version 16.0 (StataCorp, Texas, USA) was used to perform the analysis. For NMA in Stata software, a multivariate meta-analysis package was installed and utilized. Performing NMA using the Stata software in this review generally followed the methodology described by Shim and Yoon (40). The data entered into Stata were converted into analysis data through network setup. If the number of occurrences was zero (d = 0), this was corrected using the augmented method and then included in the analysis. That is, a default value of 0.5 was assigned to the intervention group and the control group instead of 0. This increased the sample size per treatment by 1 (40). The reference group was set up as a wait-list group. For the assessment of consistency, inconsistency and consistency models were tested by using the design by treatment interaction model (i.e., global approach) and node-splitting test identifying the statistical difference between direct and indirect comparisons for each treatment (i.e., local approach), respectively. The effect sizes and 95% CIs between each intervention were presented as network forest and intervalplot. In addition, network rank and surface under the cumulative ranking probabilities (SUCRA) were used to confirm comparative advantages between the treatments. In SUCRA, a cumulative probability graph is drawn, and the area under the curve (AUC) is calculated for each treatment, allowing for ranking comparison. Finally, raw data of effect size by treatment was described through the network league table.

#### Additional Analysis

If sufficient studies were available, we performed subgroup analyses for the primary outcomes according to the disease period (>3 months, which means chronic insomnia) to investigate sources of potential inconsistency or heterogeneity. If sufficient studies were available, we performed sensitivity analyses for the primary outcomes to identify the robustness of meta-analysis results by only including studies with low risks of bias, having a low risk of bias in all domains. Moreover, the robustness of meta-analysis results was also confirmed by removing outliers.

### Publication Bias

In NMA, there is no validated statistical test method other than visual confirmation using a funnel plot for the detection of publication bias. In addition, conventional funnel plots used in the pair-wise meta-analysis cannot assess publication bias in NMA. Therefore, in this review, we tried to identify the asymmetry of the network funnel plot for the primary outcomes to detect the possibility of publication bias.

### Quality of Evidence

We assessed the quality of evidence regarding the effect estimates derived from NMA for the primary outcome measures using the Grading of Recommendations Assessment, Development and Evaluation (GRADE) approach (41). For direct comparisons, we assessed the risk of bias, indirectness, imprecision, inconsistency, and publication bias. For indirect comparisons, the lowest ratings of the two direct comparisons forming the most dominant first-order loop and intransitivity were considered. The higher rating of the direct or indirect estimates was applied to the quality of evidence for NMA and categorized as high, moderate, low, or very low.

## Results

### Characteristics of Included Studies

**Figure 1:**
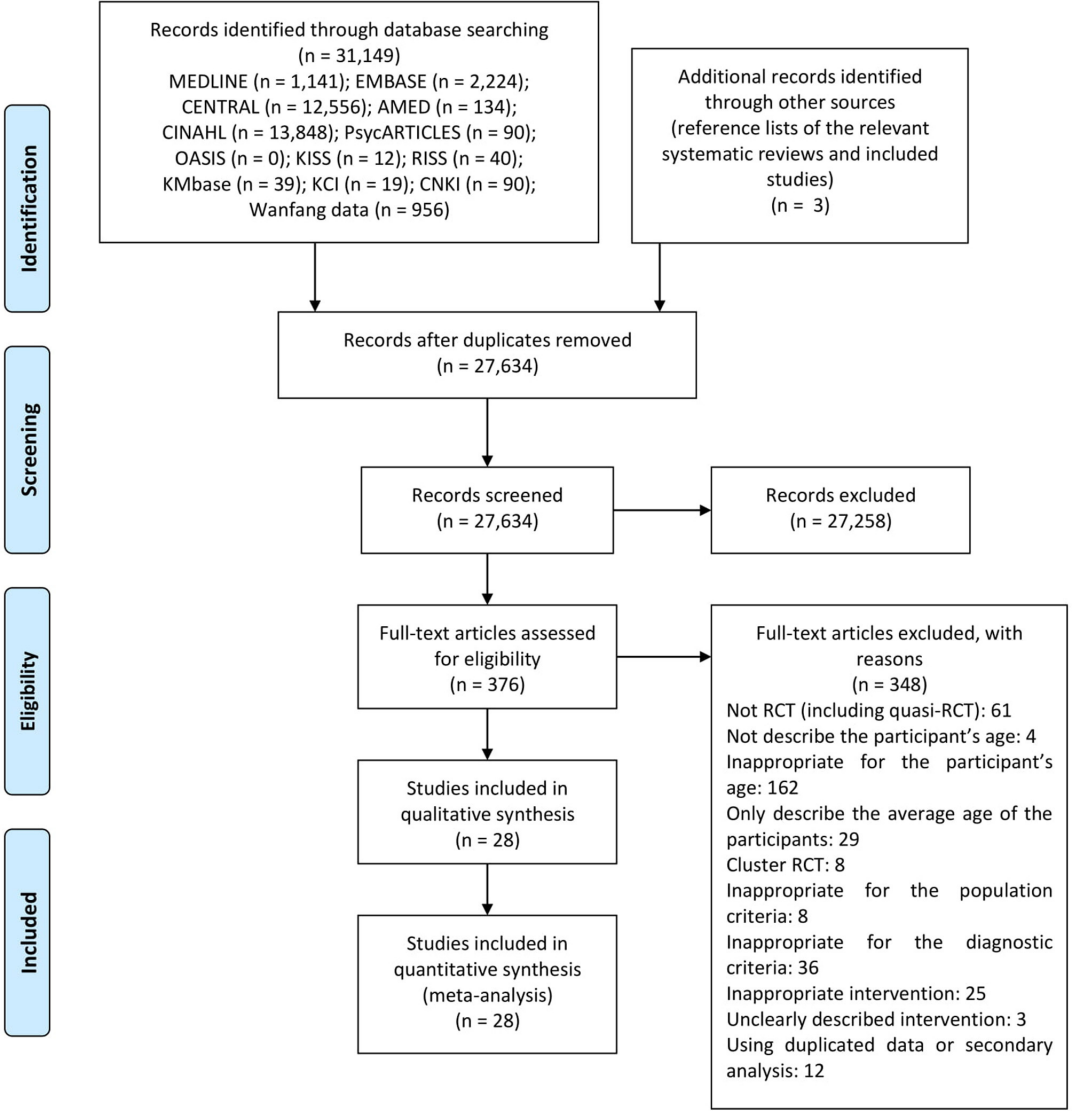
PRISMA flow chart. AMED, the allied and complementary medicine database; CENTRAL, the Cochrane central register of controlled trials; CINAHL, the cumulative index to nursing and allied health literature; CNKI, China national knowledge infrastructure; KCI, Korea citation index; KISS, Koreanstudies Information Service System; KMbase, Korean medical database; OASIS, Oriental medicine advanced searching integrated system; RCT, randomized controlled trial; RISS, research information service system.

A total of 31,149 citations and three citations were identified through the database search and manual search, respectively. After screening of title and abstract and careful review of full-text, 28 RCTs (42–69) with 2,391 participants in 64 arms were included in this review (Figure 1). The following 17 kinds of interventions were used: (1) acupuncture, (2) acupuncture combined with benzodiazepines, (3) acupuncture combined with CBT, (4) acupuncture combined with relaxation, (5) acupuncture combined with sleep education, (6) attention control, (7) benzodiazepines, (8) benzodiazepines combined with CBT, (9) benzodiazepines combined with exercise, (10) BT, (11) CBT, (12) sleep education, (13) selective serotonin reuptake inhibitor (SSRI), (14) melatonin, (15) qigong, (16) relaxation, and (17) wait-list.

The mean sample size of included RCTs was 85.39, and the range of participants' ages ranged from 63 to 85. Five studies (17.86%) were conducted in America (42, 45, 47, 55, 58), while 22 (78.57%) were conducted in China (43, 44, 46, 48–54, 57, 59–69), and the remaining one (3.57%) in Iran (56). Twenty studies (71.43%) described the mean disease duration, ranging from 3 months to 18 years (42, 45, 48, 49, 51–55, 57–59, 61–66, 68, 69). Nineteen studies (67.86%) described the participants' baseline PSQI total scores, ranging from 9 to 17 points (47–49, 51–57, 59, 60, 62–65, 67–69). Converting 1 month and 30 days to 4 weeks, the average treatment duration in the included studies was 6 weeks, except for one study with an unclear treatment duration (50). One study (3.57%) was a four-arm study (65), six studies (21.43%) were three-arm studies (46, 48, 52, 54, 63, 64), and the remaining 21 studies (75%) were two-arm studies (42–45, 47, 49–51, 53, 55–62, 66–69). As outcomes of insomnia severity, PSQI was used the most (n = 20, 71.43%) (46–49, 51–57, 59–64, 67–69), followed by total effective rate (TER) (*n* = 14, 50.00%) (43, 50, 52, 54, 57, 59–61, 63, 64, 66–69), sleep diary (*n* = 6, 21.43%) (42, 45, 47, 55, 58, 66), polysomnography data (*n* = 4, 14.29%) (42, 44, 47, 50), the Athens Insomnia Scale (AIS) (*n* = 3, 10.71%) (46, 49, 63), ISI (*n* = 2, 7.14%) (55, 59), and actigraphy data (*n* = 2, 7.14%) (55, 58). In addition, outcomes of mental health such as the Hamilton Depression Rating Scale (HAMD), the Hamilton Anxiety Rating Scale (HAMA), the Geriatric Depression Scale (GDS) were used in nine studies (32.14%) (42, 47, 48, 51, 53, 55, 57, 66, 69). Two studies (7.14%) used outcomes related to quality of life such as the 36-Item Short Form Survey (SF-36) (47, 69). No study reported AE by TESS, while there were 10 studies reporting the incidence of AE during study (43, 45, 47, 48, 51, 52, 63–65, 69). The baseline characteristics of each study are presented in Table 1. Excluded studies and reasons are presented in Supplementary Tables 15–26.

**Table 1:** Characteristics of included randomized controlled trials.

**Study ID (Country)**	**Sample size (include → analyzed)**	**Mean age (range) (yr)**	**Diagnostic criteria (and severity)**	**Duration (range)**	**(A) Treatment intervention**	**(B) Control intervention**	**Duration of treatment/** **F/U**	**Outcomes**	Jadad score^*^ (Q1–5)
Morin et al. (42) (America)	24 (12:12) → 24 (12:12)	67.1 ± 5.3 (NR)	ICSD-I	13 ± 14.1 yr (NR)	CBT-I	Wait-list	8 wk/3, 12 mon	1. Sleep diary 2. Polysomnography 3. Self-made questionnaire (sleep-related distress, severity, interference, etc.) 4. BDI 5. STAI 6. POMS	1 (1/0/0/0/0)
Song et al. (43) (China)	96 (48:48) → 96 (48:48)	(A) 65 (60–78) (B) 67 (63–80)	CCMD-2-R	(A)5–54 mon (NR) (B) 6–52 mon (NR)	ACU, relaxation	Paroxetine 10–20 mg qd	10 wk/NA	1. TER	1 (1/0/0/0/0)
Yang et al. (44) (China)	82 (40:42) → 82 (40:42)	71.4 ± 6.27 (NR)	CCMD-3	NR	Zolpidem 10 mg qd, CBT-I	Zolpidem 10 mg qd	3 mon/NA	1. Self-made questionnaire (sleep hygiene evaluation) 2. Polysomnography	2 (1/1/0/0/0)
McCrae et al. (45) (America)	24 (unclear) → 20 (11:9)	77.2 ± 8.0 (NR)	ICSD-II, DSM-4 (Average number of insomnia nights/wk was 4.7 (SD: 1.5).)	10.6 ± 17.0 yr (1.5–62.0)	Multicomponent behavioral treatment	Sleep hygiene education	4 wk/NA	1. Sleep diary	3 (1/1/0/0/1)
Weng and Liao (46) (China)	78 (26:26:26) → 78 (26:26:26)	70.71 (NR)	CCMD-3	NR	(A1) EA (A2) EA, estazolam 1 mg qd	Estazolam 1 mg qd	4 wk/NA	1. PSQI 2. AIS	1 (1/0/0/0/0)
Buysse et al. (47) (America)	82 (42:40) → 79 (39:40)	(A) 72.5 ± 6.6 (NR) (B) 70.8 ± 7.8 (NR)	DSM-IV-TR, ICSD-2 (PSQI (A) 10.44 ± 0.48 (B) 10.38 ± 0.47)	NR	BBTI	Information control	4 wk/6 mon	1. HAMD 2. HAMA 3. PSQI 4. Epworth Sleepiness Scale 5. SF-36 6. Sleep diary 7. Actigraphy 8. Polysomnography	3 (1/1/0/0/1)
Lin et al. (48) (China)	150 (50:50:50) → 133 (46:43:44)	(A1) 67.88 ± 4.38 (NR) (A2) 67.75 ± 4.80 (NR) (B) 66.21 ± 7.68 (NR)	ICSD-R (PSQI > 7 (A1) 17.78 ± 2.26 (A2) 17.81 ± 2.21 (B) 17.66 ± 2.42)	(A1) 4.67 ± 3.14 yr (NR) (A2) 4.92 ± 2.43 yr (NR) (B) 4.93 ± 2.62 yr (NR)	(A1) ACU (A2) ACU, biofeedback relaxation	Biofeedback relaxation	8 wk/6 mon	1. PSQI 2. HAMA 3. HAMD	3 (1/1/0/0/1)
Zhu et al. (49) (China)	220 (111:109) → 220 (111:109)	(A) 71.03 ± 8.38 (60–79) (B) 71.54 ± 6.34 (60–79)	CCMD-3 (PSQI 9.77 ± 1.82 (A) 9.73 ± 1.71)	1.2 ± 0.59 yr (0.5–2) (B) 1.2 ± 0.66 yr (0.5–2)	1. Aerobic exercise 2. Estazolam 0.5–1.5 mg qd	Estazolam 0.5–1.5 mg qd	8 wk/NA	1. PSQI 2. AIS 3. Cardiopulmonary exercise testing 4. TER	3 (1/1/0/0/1)
Liu (50) (China)	67 (34:33) → 67 (34:33)	(A) 83 ± 2.5 (80–88) (B) 85 ± 3.4 (82–90)	CCMD-3	NR	Acupressure, sleep education	Sleep education	NR	1. TER 2. Polysomnography	2 (1/0/0/0/1)
Ren and Li (51) (China)	64 (32:32) → 64 (32:32)	69.8 ± 7.8 (65–79)	ICSD-II (PSQI ≥ 8 (A) 14.38 ± 2.24 (B) 13.56 ± 2.50)	21.3 ± 6.7 mon (NR)	ACU	Melatonin capsule (melatonin 3 mg qd)	4wk/NA	1. PSQI 2. GDS	3 (1/1/0/0/1)
Wang et al. (52) (China)	98 (33:35:30) → 98 (33:35:30)	(A1) 73 ± 6 (65–84) (A2) 73 ± 6 (65–81) (B) 73 ± 6 (65–87)	CCMD-3 (PSQI (A1) 15.73 ± 3.79 (A2) 15.86 ± 3.75 (B) 15.67 ± 3.67)	(A1) 15.0 ± 7.1 mon (4–26) (A2) 15.0 ± 6.9 mon (3–26) (B) 15.1 ± 7.3 mon (6–28)	(A1) ACU (A2) ACU, estazolam 1 mg qd, oryzanol 20 mg tid	Estazolam 1 mg qd, oryzanol 20 mg tid	4 wk/4 wk	1. PSQI 2. Clinical effective rate	2 (1/1/0/0/0)
Zhang et al. (53) (China)	64 (32:32) → 64 (32:32)	(A) 78.57 ± 2.94 (NR) (B) 77.63 ± 3.01 (NR)	DSM-IV (PSQI (A) 11.50 ± 3.28 (B) 11.27 ± 3.62)	6 mon	MBSR	Wait-list	8 wk NA	1. PSQI 2. SAS 3. GDS	3 (1/1/0/0/1)
Xu et al. (54) (China)	81 (27:27:27) → 81 (27:27:27)	68.15 ± 7.25 (60–72)	CCMD-3 (PSQI (A1) 14.02 ± 3.58 (A2) 14.21 ± 3.84 (B) 14.05 ± 3.28)	6.51 ± 2.18 yr (3 mon−10 yr)	(A1) CBT (A2) CBT, oxazepam 15 mg 1T qd	Oxazepam 15 mg 1T qd	4 wk/NA	1. TER 2. PSQI 3. Serum levels of IL-1 (ng/L), IL-6 (ng/L), TNF-α (ug/ml), and cortisol (ug/L)	2 (1/1/0/0/0)
Alessi et al. (55) (America)	159 (106:53) → 159 (106:53)	(A) 72.2 ± 7.7 (NR) (B) 72.1 ± 7.9 (NR)	ICSD-2 (PSQI (A) 9.4 ± 3.5 (B) 8.3 ± 3.2)	3 mon	CBT-I	Sleep education	6 wk/6, 12 mon	1. Sleep diary 2. Sleep efficiency (Actigraphy) 3. PSQI 4. ISI 5. PHQ-9 6. 12-item Short-Form Study	5 (1/1/1/1/1)
Reza (56) (Iran)	44 (22:22) → 39 (19:20)	(A) 69.21 ± 5.96 (NR) (B) 66.70 ± 5.89 (NR)	DSM-IV (PSQI>5 (A) 12.95 ± 2.73 (B) 12.7 ± 2.96)	NR	CBT	Wait-list	4 wk/3mon	1. PSQI	2 (1/0/0/0/1)
Duan (57) (China)	78 (39:39) → 78 (39:39)	(A) 72.19 ± 13.58 (NR) (B) 73.74 ± 13.26 (NR)	ICSD (PSQI (A) 14.26 ± 2.32 (B) 13.98 ± 2.53)	(A) 21.5 ± 6.7 mon (NR) (B) 21.7 ± 6.3 mon (NR)	ACU	Melatonin capsule (melatonin 2.05 mg) qd	4 wk/NA	1.PSQI 2. GDS 3. TER	2 (1/0/0/0/1)
Chan et al. (58) (America)	62 (32:30) → 62 (32:30)	(A) 67.97 ± 5.97 (NR) (B) 71.03 ± 9.06 (NR)	ICSD-II	(A) 9.51 ± 12.37 yr (NR) (B) 18.55 ± 16.95 yr (NR)	BBTI	Attention control	4 wk/3 mon	1. Sleep diary 2. Actigraphy	3 (1/1/0/0/1)
Liang (59) (China)	70 (35:35) → 70 (35:35)	(A) 68 ± 6 (61–64) (B) 67 ± 7 (60–75)	CCMD-3 (PSQI (A) 13.97 ± 3.05 (B) 14.02 ± 2.64)	(A) 5.37 ± 2.66 yr (0.5–11) (B) 5.44 ± 3.12 yr (1–12)	Ear acupuncture	Estazolam 1 mg 1T qod	30 d/NA	1. TER 2. PSQI 3. ISI	2 (1/1/0/0/0)
Lin et al. (60) (China)	90 (46:44) → 90 (46:44)	NR	ICD-10, DSM-IV, CCMD-3 (15 ≥ PSQI ≥ 7 (A) 12.30 ± 1.35 (B) 12.63 ± 1.44)	NR	Ear acupressure	Estazolam 1 mg 1T qd	4 wk/NA	1.PSQI 2. TER	2 (1/0/0/0/1)
Xue et al. (61) (China)	80 (40:40) → 80 (40:40)	(A) 69.1 ± 2.15 (60–78) (B) 69.02 ± 2.14 (61–79)	Criteria from Chinese Medical Association Neurology Branch Sleep Disorders Group	(A) 1.86 ± 0.35 yr (7 mon−3 yr) (B) 1.87 ± 0.34 yr (6 mon−3 yr)	ACU	Estazolam 1–2 mg qd	2 mon/NA	1. TER 2.PSQI (total score was not presented) 3. Transcranial doppler (systolic flow velocity of the vertebral artery and basilar artery)	3 (1/0/1/0/1)
Zhang (62) (China)	160 (80:80) → 160 (80:80)	66.3 ± 4.2 (65–82) (B) 68.5 ± 3.2 (65–80)	Chinese Guideline of Adult Insomnia Diagnosis and Treatment 2012 Edition ≥ PSQI ≥ 21 18.3 ± 3.1 (B) 19.1 ± 3.4)	(A) 23 ± 3.8 mon (6 mon−10 yr) (B) 26 ± 4.2 mon (6 mon−13 yr)	CBT-I, estazolam 2 mg qd	Estazolam 2 mg qd	8 wk/6 mon	1. PSQI (the score was not presented) 2. Drug reduction rate	2 (1/0/0/0/1)
Chen et al. (63) (China)	90 (30:30:30) → 90 (30:30:30)	(A) 63.4 ± 2.4 (61–68) (B1) 64.4 ± 2.5 (60–69) (B2) 64.6 ± 2.7 (60–70)	CCMD-3 (PSQI (A) 14.2 ± 0.72 (B1) 14.1 ± 0.73 (B2) 13.9 ± 0.80)	(A) 2.7 ± 1.5 yr (6 mon−7 yr) (B1) 3.0 ± 1.5 yr (1–8) (B2) 2.5 ± 1.5 yr (2–7)	(A) ACU	(B1) Alprazolam 0.4 mg 1T qd (B2) HM, ACU	1 mon/NA	1. TER 2. PSQI 3. AIS	2 (1/0/0/0/1)
Mo (64) (China)	90 (30:30:30) → 83 (27:27:29)	(A1) 69.78 ± 7.21 (NR) (A2) 71.07 ± 6.57 (NR) (B) 70.21 ± 6.39 (NR)	CCMD-3 (PSQI > 7 PSQI (A1) 14.30 ± 1.41 (A2) 13.52 ± 1.67 (B) 13.55 ± 1.53)	(A1) 10.48 ± 9.37 mon (NR) (A2) 10.37 ± 11.90 mon (NR) (B) 10.59 ± 9.42 mon (NR)	(A1) ACU (method A) (A2) ACU (method B)	Estazolam 1 mg qd	4 wk/1 mon	1. TER 2. PSQI 3. FS-14 4. Recurrence rate	3 (1/1/0/0/1)
Yuan et al. (65) (China)	120 (30:30:30:30) → 120 (30:30:30:30)	(A) 67.4 ± 6.00 (NR) (B1) 65.5 ± 5.12 (NR) (B2) 66.3 ± 4.23 (NR) (B3) 65.5 ± 5.12 (NR)	CCMD-3 (PSQI (A) 13.21 ± 2.01 (B1) 14.62 ± 1.85 (B2) 14.62 ± 1.85 (B3) 14.1 ± 3.60)	(A) 13.4 ± 3.28 mon (NR) (B1) 13.2 ± 10.25 mon (NR) (B2) 12.7 ± 9.65 mon (NR) (B3) 14.2 ± 5.03 mon (NR)	(A) ACU	(B1) Estazolam 1 mg 1T qd (B2) HM (B3) HM, ACU	4 wk/NA	1. TER 2. PSQI 3. TCM symptom score	2 (1/1/0/0/0)
Xu et al. (65) (China)	86 (43:43) → 86 (43:43)	(A) 73.4 ± 11.6 (NR) (B) 74.5 ± 12.1 (NR)	ICSD-3 (self-rating scale (not specified)>40)	(A) 6.8 ± 1.1 yr (NR) (B) 7.1 ± 1.3 yr (NR)	CBT, ACU	CBT	8 wk/NA	1. TER (ISI score) 2. Sleep diary 3. SAS 4. SDS	2 (1/1/0/0/0)
Liu (67) (China)	78 (39:39) → 78 (39:39)	(A) 75.20 ± 4.38 (66–83) (B) 75.18 ± 4.32 (69–82)	CCMD (PSQI (A) 15.10 ± 2.23 (B) 15.15 ± 2.20)	NR	Estazolam 1 mg 1T qd, oryzanol 10 mg 2T tid, ACU	Estazolam 1 mg 1T qd, oryzanol 1 0 mg 2T tid	4 wk/NA	1. PSQI 2. TER (clinical symptom)	2 (1/1/0/0/0)
Yu and Gao (68) (China)	60 (30:30) → 56 (28:28)	(A) 71.3 ± 5.7 (60–80) (B) 72.3 ± 4.8 (63–79)	CCMD-3 (PSQI ≥ 7 (A) 14.82 ± 2.07 (B) 14.29 ± 2.67)	(A) 4.43 ± 2.50 yr (0.5–10) (B) 4. 99 ± 2.44 yr (1–11)	ACU	Estazolam 1–2 mg 1T qd	4 wk/NA	1. PSQI 2. TER (PSQI score) 3. MoCA	3 (1/1/0/0/1)
Wei (69) (China)	74 (37:37) → 61 (32:29)	(A) 66.76 ± 3.58 (NR) (B) 66.24 ± 4.30 (NR)	DSM-5 (PSQI (A) 13.38 ± 3.22 (B) 12.76 ± 3.86)	(A) 29.21 ± 40.57 mon (NR) (B) 30.95 ± 42.25 mon (NR)	Qigong	CBT	8 wk/NA	1. TER (clinical symptom) 2. PSQI 3. SF-36 4. SAS 5. SDS	3 (1/1/0/0/1)

*ACU, acupuncture; AIS, Athens insomnia scale; BBTI, brief behavioral treatment for insomnia; BDI, Beck depression inventory; CBT-I, cognitive behavioral therapies for insomnia; CCMD, Chinese classification of mental disorders; DSM, diagnostic and statistical manual of mental disorders; EA, electro-acupuncture; FS-14, fatigue scale-14; GDS, geriatric depression scale; HAMA, Hamilton anxiety rating scale; HAMD, Hamilton depression rating scale; HM, herbal medicine; ICSD, international classification of sleep disorders; IL, interleukin; ISI, insomnia severity index; MBSR, mindfulness-based stress reduction; MoCA, Montreal cognitive assessment; NA, not applicable; NR, not reported; PHQ-9, patient health questionnaire-9; POMS, profile of mood states; PSQI, Pittsburgh sleep quality index; SAS, self-rating anxiety scale; SDS, self-rating depression scale; SF-36, 36-item short form survey; STAI, state-trait anxiety inventory; TCM, traditional Chinese medicine; TER, total effective rate; TNF, tumor necrosis factor*.

Among the included studies, Mo (64) with three-arm, compared two different kinds acupuncture to benzodiazepines (i.e., estazolam). Therefore, we divided Mo (64) into Mo (2018a) and Mo (2018b) in the data analysis to compare the two acupuncture methods and benzodiazepines, respectively. That is, Mo (2018a) included the whole value of the first acupuncture group and half value of the control group, and Mo (2018b) included the whole value of the second acupuncture group and half value of the control group. Chen et al. (63), a three-arm RCT, included herbal medicine combined with acupuncture group, that did not meet our intervention criteria. Moreover, Yuan et al. (65), a four-arm RCT, included herbal medicine combined with acupuncture group and herbal medicine group. Therefore, those groups including herbal medicine were excluded from our analysis.

* The five questions (Q1–5) for the Jadad score were as follows: (1) Was the study described as randomized? (2) Was the appropriate randomization method applied? (3) Was the study described as double-blind? (4) Was the appropriate blinding method applied? (5) Was there a description of withdrawals and dropouts?

### Risk of Bias of Included Studies

Regarding the Jadad scale, the average score was 2.39 and only 12 studies had scores of 3 or higher. Regarding the risk of bias tool, most of the included studies used random sequence generation methods with low risk of bias (44, 45, 47–49, 51–55, 58, 59, 64–69), such as random number tables or simple randomization, while some studies without a description of randomization method was rated to have an unclear risk of bias on this domain (42, 43, 46, 50, 56, 57, 60–63). Except for three studies that used sealed opaque envelopes for allocation concealment (55, 59, 69), the rest of the studies did not describe allocation concealment. Only Alessi et al. (55) performed blinding on both participants and personnel, and outcome assessors. Among the remaining studies, 24 studies did not describe the blinding of participants and personnel, and the risk of performance bias was rated as high due to the nature of the intervention (42–52, 54, 56–60, 62–68). Two studies described that double-blinding was not performed (53, 69). Xue et al. (61) described that they performed double-blinding but did not describe the method, therefore, the risk of bias for the domain was assessed as unclear. Lin et al. (48) and Alessi et al. (55) described that they performed the blinding of outcome assessors. In 18 studies, there were no drop-out cases (42–44, 46, 49–52, 54, 57, 59–63, 65–67). Some drop-out cases existed in the remaining studies; however, the numbers of drop-out cases were considered to not affect the study results (45, 47, 48, 56, 64, 68, 69), and/or appropriate statistical analysis (i.e., intent-to-treat analysis) was applied (53, 55, 58). One study that did not report some of outcome data in the control group (44), two studies that did not preset the raw PSQI data collected (43, 62), one study that did not present the PSQI total score (61), and one study that did not present the raw ISI data collected (66) were assessed as having a high risk of bias in the selective reporting domain. Regarding other biases, according to our protocol, 25 studies described statistical homogeneity between groups in the baseline were evaluated as having a low risk of bias (42–50, 52–57, 59, 61–69) (Supplementary Figures 1, 2). Only seven studies (47, 53, 55, 56, 58, 65, 67) described approval from the Institutional Review Board (IRB), and 19 studies (47, 48, 50–53, 55–65, 68, 69) described that they had received participants' consent before the trial.

### Comparative Effectiveness

#### PSQI Total Score (Primary Outcome)

##### NMA

**Figure 2:**
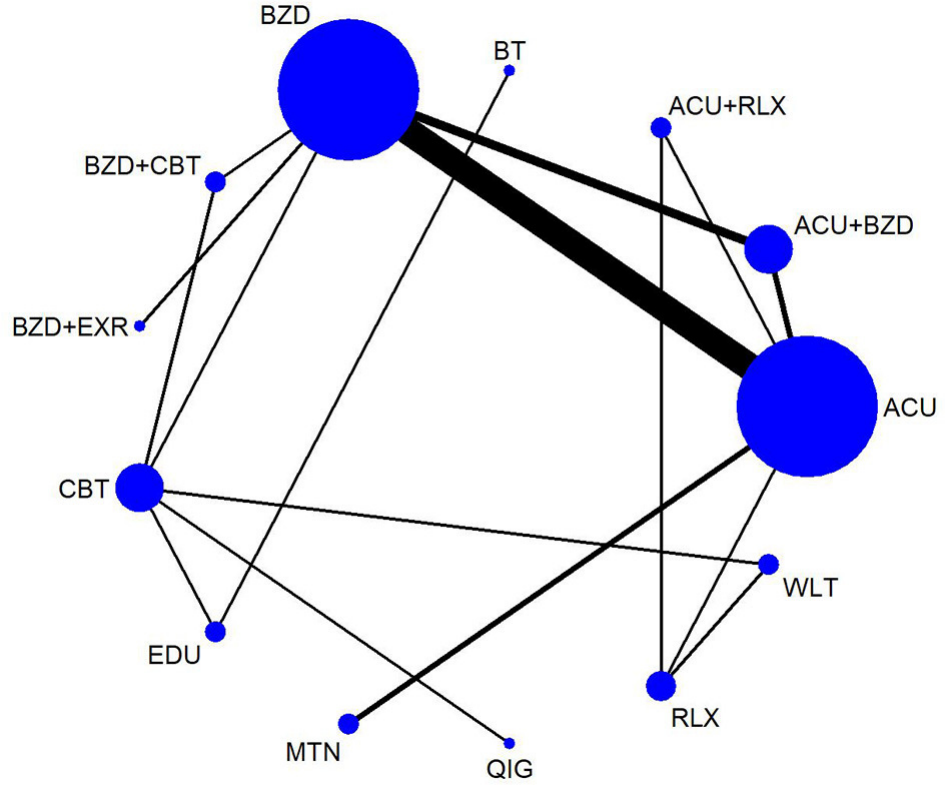
Network map for PSQI total score. ACU, acupuncture; BT, behavioral treatment; BZD, benzodiazepines; CBT, cognitive behavioral therapy; EDU, sleep education; EXR, exercise; MTN, melatonin; QIG, qigong; RLX, relaxation.

**Figure 3:**
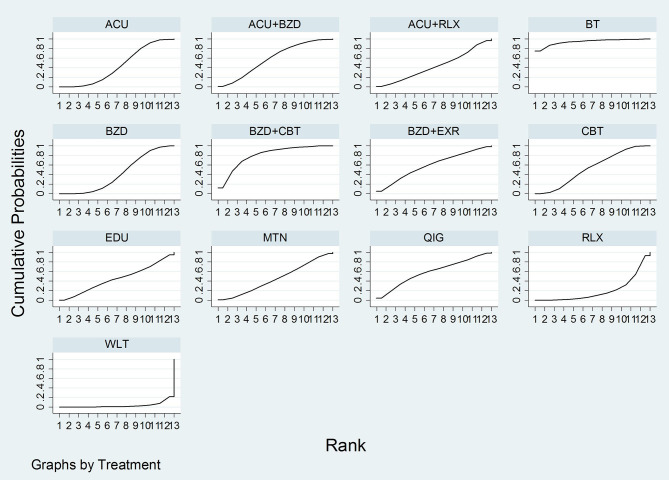
SUCRA for PSQI total score. ACU, acupuncture; BT, behavioral treatment; BZD, benzodiazepines; CBT, cognitive behavioral therapy; EDU, sleep education; EXR, exercise; MTN, melatonin; QIG, qigong; RLX, relaxation.

Through NMA for PSQI total score, we estimated the relative effect of the 13 interventions (Figure 2). The consistency model could be accepted (*p*-value of testing for inconsistency = 0.1901, of node-splitting test = 0.104 to 0.988). Based on the mean rank and SUCRA, the priorities in terms of effectiveness measured by PSQI total score were as follow: (1) BT, (2) benzodiazepines combined with CBT-I, (3) acupuncture combined with benzodiazepines, (4) benzodiazepines combined with exercise, (5) qigong, (6) CBT-I, (7) melatonin, (8) sleep education, (9) acupuncture, (10) acupuncture combined with relaxation, (11) benzodiazepines, (12) relaxation, and (13) wait-list. According to the netleague table presenting the comparative effectiveness of treatments (in favor of bolding marks), **acupuncture** (SMD −4.37, 95% CI −8.53 to −0.12), **acupuncture combined with benzodiazepines** (SMD −5.20, 95% CI −9.82 to −0.57), **BT** (SMD −10.44, 95% CI −17.31 to −3.58), **benzodiazepines** (SMD −4.28, 95% CI −8.45 to −0.11), **benzodiazepines combined with CBT** (SMD −7.18, 95% CI −12.17 to −2.19), and **CBT** (SMD −4.93, 95% CI −8.63 to −1.22) were all statistically significantly superior to wait-list. Moreover, sleep education and relaxation were both statistically significantly inferior to **BT** (SMD 6.13, 95% CI 1.98 to 10.28; SMD 8.00, 95% CI 0.87 to 15.13). Compared to wait-list, acupuncture combined with relaxation (SMD −4.18, 95% CI −9.08 to 0.72), benzodiazepines combined with exercise (SMD −5.41, 95% CI −11.21 to 0.38), melatonin (SMD −4.38, 95% CI −9.42 to 0.67), and qigong (SMD −5.46, 95% CI −10.94 to 0.02) showed superior benefits, with borderline significances. However, sleep education (SMD −4.31, 95% CI −9.78 to 1.15), or relaxation (SMD −2.44, 95% CI −5.98 to 1.09) did not show significant or near-significant differences with the wait-list (Table 2, Figure 3, Supplementary Figures 3–9, Supplementary Table 27).

**Table 2:** Head-to-head comparisons for effectiveness and acceptability of the non-pharmacological interventions.

**WLT**	**−4.37** **(−8.53,** **−0.21)**	**−5.20** **(−9.82,** **−0.57)**	**–**	**–**	**−4.18** **(−9.08,** **0.72)**	**–**	**−10.44** **(−17.31,** **−3.58)**	**−4.28** **(−8.45,** **−0.11)**	**−7.18** **(−12.17,** **−2.19)**	**−5.41 (−11.21, 0.38)**	**−4.93** **(−8.63,** **−1.22)**	**−4.31** **(−9.78,** **1.15)**	**−4.38** **(−9.42,** **0.67)**	**−5.46** **(−10.94,** **0.02)**	**−2.44** **(−5.98,** **1.09)**	**–**
1.06 (0.11, 10.66)	**ACU**	−0.82 (−3.10, 1.45)	–	**–**	0.19 (−3.72, 4.11)	**–**	**−6.07 (−12.91, 0.77)**	0.09 (−1.23, 1.42)	−2.81 (−6.91, 1.30)	−1.04 (−5.27, 3.19)	−0.55 (−4.22, 3.11)	0.06 (−5.38, 5.50)	−0.01 (−2.86, 2.85)	−1.09 (−6.54, 4.37)	1.93 (−1.60, 5.46)	**–**
1.08 (0.05, 23.85)	1.02 (0.12, 8.68)	**ACU+BZD**	–	**–**	1.02 (−3.48, 5.51)	**–**	−5.25 (−12.34, 1.85)	0.92 (−1.25, 3.09)	−1.99 (−6.47, 2.50)	−0.22 (−4.79, 4.35)	0.27 (−3.84, 4.38)	0.88 (−4.87, 6.63)	0.82 (−2.83, 4.47)	−0.26 (−6.03, 5.50)	2.75 (−1.38, 6.89)	**–**
0.71 (0.01, 50.32)	0.67 (0.01, 69.85)	0.66 (<0.01, 104.67)	**ACU+CBT**	**–**	**–**	**–**	**–**	**–**	**–**	**–**	**–**	**–**	**–**	**–**	**–**	**–**
0.37 (<0.01, 121.71)	0.35 (<0.01, 153.31)	0.34 (<0.01, 209.01)	0.52 (<0.01, 477.22)	**ACU+EDU**	**–**	**–**	**–**	**–**	**–**	**–**	**–**	**–**	**–**	**–**	**–**	**–**
2.31 (0.18, 29.02)	2.18 (0.52, 9.18)	2.14 (0.16, 28.07)	3.26 (0.03, 388.24)	6.30 (0.01, 3,087.11)	**ACU+RLX**	**–**	−6.26 (−13.87, 1.34)	−0.10 (−4.18, 3.98)	−3.00 (−8.47, 2.46)	−1.24 (−6.96, 4.49)	−0.75 (−5.70, 4.20)	−0.14 (−6.51, 6.24)	−0.20 (−5.04, 4.64)	−1.28 (−7.67, 5.11)	1.73 (−2.18, 5.65)	**–**
0.03 (<0.01, 6.40)	0.03 (<0.01, 8.25)	0.03 (<0.01, 11.52)	0.04 (<0.01, 26.97)	0.08 (<0.01, 13.74)	0.01 (<0.01, 4.22)	**ATC**	**–**	**–**	**–**	**–**	**–**	**–**	**–**	**–**	**–**	**–**
0.05 (<0.01, 9.02)	0.05 (<0.01, 11.73)	0.05 (<0.01, 16.51)	0.07 (<0.01, 39.03)	0.14 (<0.01, 19.22)	0.02 (<0.01, 6.02)	1.64 (0.46, 5.88)	**BT**	**6.16 (−0.61, 12.94)**	3.26 (−3.74, 10.26)	5.03 (−2.85, 12.91)	**5.52 (−0.27, 11.30)**	**6.13** **(1.98, 10.28)**	6.06 (−1.35, 13.48)	4.98 (−2.07, 12.04)	**8.00** **(0.87, 15.13)**	**–**
1.03 (0.10, 10.99)	0.97 (0.39, 2.40)	0.95 (0.12, 7.69)	1.45 (0.01, 152.62)	2.80 (0.01, 1,249.05)	0.44 (0.08, 2.40)	34.04 (0.12, 9,929.17)	20.71 (0.08, 5,225.40)	**BZD**	−2.90 (−6.84, 1.04)	−1.14 (−5.15, 2.88)	−0.65 (−4.19, 2.89)	−0.04 (−5.39, 5.32)	−0.10 (−3.25, 3.05)	−1.18 (−6.55, 4.19)	1.83 (−1.82, 5.49)	**–**
0.94 (0.05, 18.00)	0.89 (0.08, 9.38)	0.87 (0.04, 18.13)	1.33 (0.01, 180.82)	2.57 (<0.01, 1,395.30)	0.41 (0.03, 6.21)	31.16 (0.09, 11,252.12)	18.96 (0.06, 5,953.63)	0.92 (0.10, 8.40)	**BZD+CBT**	1.77 (−3.86, 7.39)	2.25 (−1.70, 6.20)	2.87 (−2.77, 8.50)	2.80 (−2.20,7.80)	1.72 (−3.93, 7.37)	**4.74 (−0.23, 9.70)**	**–**
1.05 (0.01, 102.95)	0.99 (0.02, 55.70)	0.97 (0.01, 82.99)	1.48 (<0.01, 653.30)	2.85 (<0.01, 4,040.03)	0.45 (0.01, 32.58)	34.66 (0.03, 34,491.43)	21.09 (0.02, 18,635.54)	1.02 (0.02, 51.77)	1.11 (0.01, 101.21)	**BZD+EXR**	0.49 (−4.87, 5.84)	1.10 (−5.60, 7.80)	1.04 (−4.07,6.14)	−0.04 (−6.75, 6.66)	2.97 (−2.46, 8.40)	**–**
0.71 (0.14, 3.58)	0.67 (0.06, 7.85)	0.66 (0.03, 15.95)	1.00 (0.02, 51.54)	1.93 (0.01, 509.11)	0.31 (0.02, 4.58)	23.48 (0.14, 3,874.82)	14.29 (0.10, 2,005.45)	0.69 (0.06, 8.22)	0.75 (0.04, 14.17)	0.68 (0.01, 70.49)	**CBT**	0.61 (−3.41, 4.64)	0.55 (−4.09,5.19)	−0.53 (−4.57, 3.51)	2.48 (−1.69, 6.65)	**–**
0.36 (0.01, 25.07)	0.34 (<0.01, 34.83)	0.33 (<0.01, 52.23)	0.50 (<0.01, 131.73)	0.97 (0.02, 50.36)	0.15 (<0.01, 18.24)	11.80 (0.45, 305.91)	7.18 (0.36, 143.50)	0.35 (<0.01, 36.20)	0.38 (<0.01, 51.21)	0.34 (<0.01, 149.73)	0.50 (0.01, 25.67)	**EDU**	−0.06 (−6.21,6.08)	−1.14 (−6.85, 4.56)	1.87 (−3.92, 7.66)	**–**
1.06 (0.03, 39.75)	1.00 (0.06, 16.34)	0.98 (0.03, 33.17)	1.49 (0.01, 338.59)	2.89 (<0.01, 2,354.13)	0.46 (0.02, 10.62)	35.09 (0.06, 19,489.65)	21.35 (0.04, 10,413.06)	1.03 (0.05, 19.45)	1.13 (0.03, 43.55)	1.01 (0.01, 136.69)	1.49 (0.04, 61.90)	2.97 (0.01, 669.72)	**MTN**	−1.08 (−7.24, 5.08)	1.93 (−2.60, 6.47)	**–**
1.25 (0.16, 9.53)	1.18 (0.08, 18.48)	1.16 (0.04, 35.35)	1.77 (0.03, 109.60)	3.41 (0.01, 1,026.76)	0.54 (0.03, 10.55)	41.45 (0.22, 7,907.93)	25.22 (0.15, 4,111.71)	1.22 (0.08, 19.32)	1.33 (0.06, 31.97)	1.20 (0.01, 145.87)	1.77 (0.52, 6.01)	3.51 (0.06, 216.35)	1.18 (0.02, 59.55)	**QIG**	3.01 (−2.79, 8.82)	**–**
1.75 (0.21, 14.74)	1.65 (0.46, 5.94)	1.62 (0.14, 19.33)	2.46 (0.02, 247.43)	4.76 (0.01, 2,047.50)	0.76 (0.16, 3.51)	57.81 (0.21, 16,233.03)	35.18 (0.14, 8,534.35)	1.70 (0.37, 7.85)	1.86 (0.14, 24.71)	1.67 (0.02, 113.09)	2.46 (0.23, 26.86)	4.90 (0.05, 488.86)	1.65 (0.08, 35.63)	1.39 (0.10, 20.45)	**RLX**	**–**
2.31 (0.02, 249.75)	2.18 (0.03, 144.57)	2.14 (0.02, 236.73)	3.26 (0.01, 1,597.30)	6.30 (<0.01, 9,718.97)	1.00 (0.02, 51.42)	76.50 (0.07, 8,3336.72)	46.55 (0.05, 45,096.91)	2.25 (0.03, 163.24)	2.45 (0.02, 295.34)	2.21 (0.01, 739.13)	3.26 (0.03, 387.50)	6.49 (0.01, 3161.95)	2.18 (0.01, 336.63)	1.85 (0.01, 256.16)	1.32 (0.02, 90.86)	**SSRI**

*ACU, acupuncture; ATC, attention control; BT, behavioral treatment; BZD, benzodiazepines; CBT, cognitive behavioral therapy; EDU, sleep education; EXR, exercise; MTN, melatonin; PSQI, the Pittsburgh Sleep Quality Index; QIG, qigong; RLX, relaxation; SSRI, selective serotonin reuptake inhibitor; WLT, wait-list*.

*The table shows the acceptability as drop-outs for any reasons (lower left portion) and effectiveness as PSQI total score (upper right portion). Interventions are reported in order of alphabet. In case of acceptability, if the number of occurrences is zero, this is corrected by the augmented method to include in the analysis. That is, a default value of 0.5 is assigned to the intervention group and the control group instead of 0, which increase the sample size per treatment by 1. Effects are presented as odds ratio for drop-outs for any reasons and standardized mean difference for PSQI total score. The results bolding and underlined meant it had statistical significance, while only bolding results meant it had borderline significance. Comparisons between treatments should be read from left to right. The cell where the row of one treatment and the column of the other treatment meet shows the estimated effect size. In the case of effectiveness, if the mean difference is <0, it means that the effectiveness of the column treatment is better. In the case of acceptability, if the odds ratio is <1, it means that the acceptance of row treatment is better*.

##### Pair-wise meta-analysis

As results of pair-wise meta-analysis, statistically significant differences existed between the following comparisons (in favor of bolding marks): (1) acupuncture vs. **acupuncture combined with relaxation** (SMD 0.56, 95% CI 0.14 to 0.99), (2) **acupuncture** vs. relaxation (SMD −0.50, 95% CI −0.92 to −0.08), (3) **acupuncture combined with benzodiazepines** vs. benzodiazepines (SMD −1.24, 95% CI −1.56 to −0.91; *I*^2^ = 95%), (4) **acupuncture combined with relaxation** vs. relaxation (SMD −1.00, 95% CI −1.44 to −0.55), (5) **BT** vs. sleep education (SMD −6.13, 95% CI −7.20 to −5.05), (6) benzodiazepines vs. **benzodiazepines combined with CBT** (SMD 2.51, 95% CI 1.78 to 3.23), (7) benzodiazepines vs. **benzodiazepines combined with exercise** (SMD 1.14, 95% CI 0.85 to 1.42), (8) **benzodiazepines** vs. CBT (SMD −2.77, 95% CI −3.53 to −2.01), (9) **benzodiazepines combined with CBT** vs. CBT (SMD −2.89, 95% CI −3.67 to −2.11), (10) **CBT** vs. sleep education (SMD −0.61, 95% CI −0.95 to −0.28), (11) CBT vs. **qigong** (SMD 0.53, 95% CI 0.02 to 1.04), (12) **CBT** vs. wait-list (SMD −6.55, 95% CI −8.20 to −4.89), and (13) **relaxation** vs. wait-list (SMD −1.04, 95% CI −1.56 to −0.52) (Supplementary Table 31).

##### Sensitivity analysis

According to network funnel plots (Supplementary Figure 26) and pair-wise meta-analysis of PSQI total scores, Weng and Liao (46) was the major outlier. After removing this outlier, in NMA, the consistency model could be accepted (*p*-value of testing for inconsistency = 0.4359, of node-splitting test = 0.172 to 0.987). According to the results of the netleague table, except for the significant difference (SMD −4.37, 95% CI −8.53 to−0.21) in the acupuncture vs. wait-list being changed to borderline significance (SMD −4.27, 95% CI −8.57 to 0.04), no change was found in the remaining significant differences, compared to before removal of the outlier (Supplementary Figure 10, Supplementary Table 28). Moreover, in the pair-wise meta-analysis, significant differences between acupuncture combined with benzodiazepines and benzodiazepines were maintained (SMD −1.47, 95% CI −1.87 to −1.07; *I*^2^ = 97%) (Supplementary Table 32).

#### Polysomnography Data (Secondary Outcome)

##### Pair-wise meta-analysis

Only four studies reported each sleep index from polysomnography data, and the network was disconnected by three components (Supplementary Figure 11). Therefore, NMA for polysomnography data was not appropriate, and only pair-wise meta-analysis was performed. As results, statistically significant differences existed between the following comparisons (the favored treatment is bolded): (1) benzodiazepines vs. **benzodiazepines combined with CBT** (SMD −3.51, 95% CI −4.22 to −2.81), (2) **CBT** vs. wait-list (SMD 0.79, 95% CI 0.20 to 1.38), (3) **acupuncture combined with sleep education** vs. sleep education (SMD 6.84, 95% CI 5.56 to 8.13) in sleep efficiency (%); (4) **BT** vs. sleep education (SMD −0.85, 95% CI −1.31 to −0.38) in WASO (min); (5) benzodiazepines vs. **benzodiazepines combined with CBT** (SMD 2.02, 95% CI 1.49 to 2.56), (6) **acupuncture combined with sleep education** vs. sleep education (SMD −2.11, 95% CI−2.72 to −1.51) in number of awakenings (*n*); (7) benzodiazepines vs. **benzodiazepines combined with CBT** (SMD −1.05, 95% CI −1.51 to −0.59), (8) **acupuncture combined with sleep education** vs. sleep education (SMD 2.38, 95% CI 1.74 to 3.01) in sleep maintenance rate (%); (9) BT vs. **sleep education** (SMD 0.75, 95% CI 0.29 to 1.21) in SOL (min); (10) BT vs. **sleep education** (SMD −0.90, 95% CI −1.36 to −0.43) in TST (min); (11) **CBT** vs. wait-list (SMD −0.99, 95% CI −1.59 to −0.39), (12) benzodiazepines vs. **benzodiazepines combined with CBT** (SMD 1.65, 95% CI 1.15 to 2.15), and (13) **acupuncture combined with sleep education** vs. sleep education (SMD −2.25, 95% CI −2.87 to −1.63) in awake duration (min) (Supplementary Table 33).

### Comparative Acceptability

#### Drop-Outs for Any Reasons (Primary Outcome)

##### NMA

**Figure 4:**
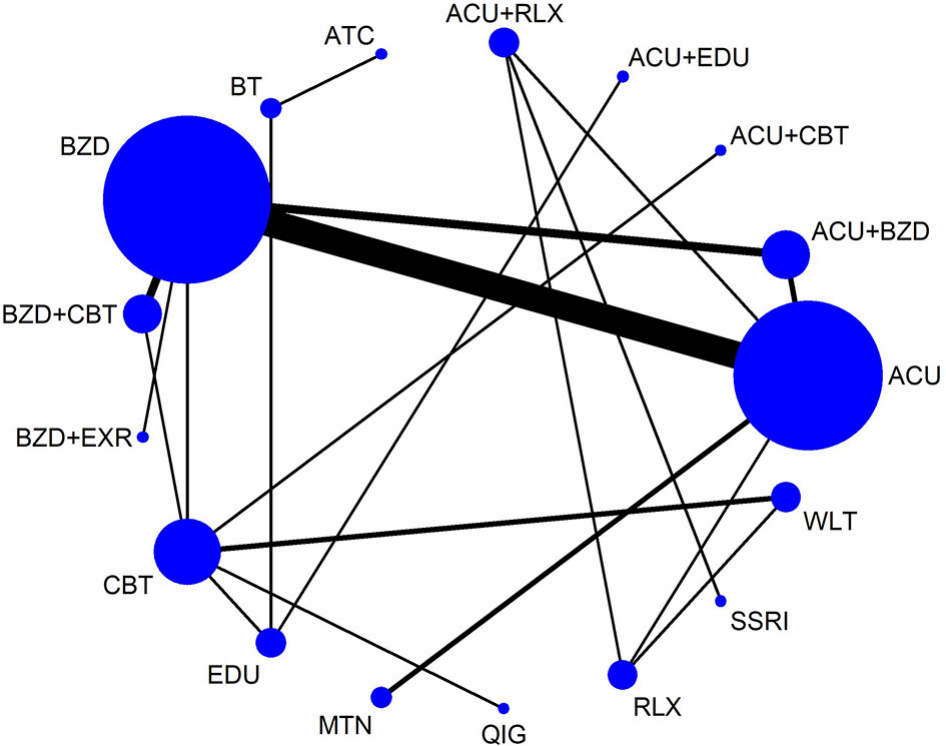
Network map for drop-outs for any reason. ACU, acupuncture; ATC, attention control; BT, behavioral treatment; BZD, benzodiazepines; CBT, cognitive behavioral therapy; EDU, sleep education; EXR, exercise; MTN, melatonin; QIG, qigong; RLX, relaxation; SSRI, selective serotonin reuptake inhibitor; WLT, wait-list.

A total of 17 interventions were analyzed for the NMA for drop-outs for any reason (Figure 4). The consistency model could be accepted (*p*-value of testing for inconsistency = 0.9993, of node-splitting test = 0.786 to 1.000). According to the netleague table presenting the comparative acceptability of treatments, there was no statistically significant head-to-head comparison. In most comparisons, the 95% CI was very wide, which is thought to be due to the very small number of events (Table 2, Supplementary Figures 12–18, Supplementary Table 29).

##### Pair-wise meta-analysis

In most studies, there were no drop-out cases. No significant differences were found between the groups in the pair-wise meta-analysis (Supplementary Table 34).

#### Drop-Outs for AEs (Secondary Outcome)

##### NMA

A total of 15 interventions were analyzed for the NMA for drop-outs for AEs. The consistency model could be accepted (*p*-value of testing for inconsistency = 1.0000, of node-splitting test = 0.980 to 1.000). According to the netleague table presenting the comparative acceptability of treatments, there was no statistically significant head-to-head comparison. In most comparisons, the 95% CI was very wide, which is thought to be due to the very small number of events (Supplementary Figures 19–25, Supplementary Table 30).

##### Pair-wise meta-analysis

In most studies, there were no drop-out cases. No significant differences were found between the groups in the pair-wise meta-analysis (Supplementary Table 35).

### Safety

#### Incidence of AEs (Secondary Outcome)

For the incidence of AEs, quantitative synthesis was judged to be inadequate because the number of AEs and the number of patients experiencing AE were mixed. There were 10 studies (43, 45, 47, 48, 51, 52, 63–65, 69) that reported the incidences of AEs. Among them, five (45, 47, 48, 51, 69) reported no AEs, while one (52) only reported some dry mouth occurred in estazolam group, without noting the exact number of episodes. In Song et al. (43), there were 18 cases of dry mouth, 16 cases of constipation, five cases of nausea, four cases of excessive sweating, and three cases of dizziness or mild headache occurred in paroxetine groups (*n* = 48), while there were no AEs in the acupuncture combined with relaxation group (*n* = 48). In Chen et al. (63), there was one case of narcolepsy, one case of dizziness, one case of fatigue, and one case of dry mouth in the alprazolam group (*n* = 30), while there were no AEs in the acupuncture group (*n* = 30). In Mo (64), there was one case of ecchymoma in each of the two acupuncture groups (both, *n* = 27), while there were two cases of dry mouth, two cases of fatigue, two cases of day sleepiness, and one case of both dry mouth and fatigue occurred in the estazolam group (*n* = 29). In Yuan et al. (65), there was one case of mild dizziness and one case of stomach discomfort in the acupuncture group (*n* = 30), while there were two cases of mild dizziness and one case of fatigue in the estazolam group (*n* = 30).

### Publication Bias

To assess publication bias, network funnel plots without reference intervention of primary outcomes were made as follows. In the PSQI total score, there was a pronounced outlier on the left side (Supplementary Figure 26), and sensitivity analysis was performed excluding this outlier, Weng and Liao (46). As a result, the removal of this outlier did not significantly affect the results of the study. In the drop-outs for any reason analysis, no cue of obvious asymmetry was observed; therefore, the probability of publication bias was considered to be low (Supplementary Figure 27).

### Quality of Evidence

The GRADE levels of NMA for the PSQI total score were mostly moderate to low (Supplementary Table 36). The GRADE levels of NMA for drop-outs for any reason were generally low (Supplementary Table 37). The main reasons for downgrading were the risk of bias and imprecision of the meta-analyzed results.

## Discussion

This systematic review with NMA conducted a comprehensive search to assess the comparative effectiveness and acceptability of non-pharmacological interventions on insomnia in the elderly. As a result, a total of 28 RCTs were included in this review.

### Summary of Evidence

In terms of methodological quality, randomizations were performed properly in most of the included studies. However, the absence of blinded participants and personnel in most included studies could have led to overestimation of the effect sizes, although this can be considered as an inevitable limitation given the basic characteristics of non-pharmacological interventions. In addition, the lack of assessor blinding in most studies suggests a risk of expectation bias and should be addressed in further trials. Proper randomization and verification of statistical similarity between groups at baseline (other bias) in the included studies support the similarity assumptions in NMA. In general, the overall quality of the RCTs included in this review was low to moderate, leading to low quality of evidence for NMA findings. High-quality studies were rare, and there was some risk of overestimation associated with a lack of blinding.

(1) In terms of comparative effectiveness for the PSQI total score, it was found that the following interventions were more effective than wait-list: acupuncture, acupuncture combined with benzodiazepines, BT, benzodiazepines, benzodiazepines combined with CBT, and CBT with statistical significance, and acupuncture combined with relaxation, benzodiazepines combined with exercise, melatonin, and qigong with borderline significance. Some interventions showed significantly better effectiveness than CBT or benzodiazepines based on pair-wise meta-analysis: benzodiazepines (*p* < 0.00001), benzodiazepines combined with CBT (*p* = 0.005) and qigong (*p* = 0.04) compared to CBT, and acupuncture combined with benzodiazepines (*p* < 0.00001), benzodiazepines combined with CBT (*p* = 0.005), and benzodiazepines combined with exercise (*p* < 0.00001) compared to benzodiazepines. Interestingly, in general, combined treatments tended to be more effective than monotherapy. In other words, benzodiazepines combined with CBT and acupuncture combined with benzodiazepines showed overall superior effectiveness in the results of NMA as well as of pair-wise meta-analysis. For the polysomnography data, similar to the results of the PSQI total score, the combined treatment showed overall superiority for some outcomes of sleep architecture. (2) In terms of comparative acceptability, NMAs with 17 interventions and 15 interventions were performed for drop-outs for any reason and any AEs. In both cases, however, the number of events occurred was very small, which did not produce meaningful results, including statistical significance in either NMA or pair-wise meta-analysis. (3) In terms of comparative safety, heterogeneity of the reported safety profiles made quantitative synthesis impossible. Although there have been few reported cases of AE, the incidence of AEs during pharmacological treatments, including estazolam, paroxetine, and alprazolam, tended to be higher than that of acupuncture. Gastrointestinal AEs such as dry mouth, constipation, and nausea were most common in the pharmacological treatment groups.

Subgroup analysis based on disease duration was planned, and the disease durations were longer than at least 3 months in all included studies that specified participants' baseline disease duration. Therefore, subgroup analysis, according to the disease duration, could not be performed. Moreover, sensitivity analyses based on the methodological quality of included RCTs were planned; however, only one study (55) was rated to be a high-quality RCT, having a low risk of bias in all domains. Given the nature of non-pharmacological interventions, even though high risks of blinding of participants and personnel domains were allowed, none of the studies rated the risks of bias in the remaining domains low. Therefore, sensitivity analyses, according to the methodological quality, could not be performed. According to the result of the network funnel plot of PSQI total score, significant asymmetry was found, and sensitivity analysis was performed to remove the outliers (46). In the sensitivity analysis to remove outliers, except for the significant difference in the acupuncture vs. wait-list being changed to borderline significance in NMA, no change was found in the remaining significant differences, compared to before removal of the outlier.

The prescription of benzodiazepines is increasing today, and the important thing is that this tendency is more pronounced in primary care than in psychiatrists (33, 70). However, the American Geriatrics Society does not recommend the use of benzodiazepines or non-benzodiazepine hypnotics in the elderly (18), as this vulnerable group may experience greater harms including fatal side effects such as falls and hip fractures (17–19). However, further evidence-based strategies still need to be established for discontinuing benzodiazepines in the elderly and some alternatives to complement these drugs (71). In this sense, to overcome the limitations of pharmacotherapies, especially of benzodiazepine in the elderly, a recent systematic review also analyzed the efficacy and safety of non-benzodiazepine and non-Z-drug hypnotic medications in elderly individuals with insomnia (72). The authors analyzed 24 clinical studies, including 21 RCTs, and concluded that limited evidence suggests suvorexant, doxepin, and possibly ramelteon may be effective and safe pharmacological alternatives for treating elderly individuals with insomnia (72). As the authors excluded non-pharmacological interventions at the study selection process, the findings of the study could be complementary to the results of this review.

In summary, this review found some comparatively effective strategies, especially combined non-pharmacological treatments, for insomnia in the elderly, while it did not find any significant comparative advantage in terms of acceptability. In the safety profile, there was limited evidence that acupuncture is overall safe. However, due to the methodological limitations of the included studies, the inability to conduct sensitivity analysis on high-quality RCTs is a limitation of the reliability of the results. In particular, strict allocation concealment and assessor blinding seem to be a major issue for further researches in this area to enhance their methodological quality.

### Clinical Interpretation

The most interesting finding of this review was that combined treatments were effective strategies for treating elderly individuals with insomnia in terms of overall effectiveness. In other words, combined treatments such as acupuncture combined with benzodiazepines and benzodiazepines combined with CBT-I showed excellent effectiveness in improving insomnia in the elderly. Based on the meanrank and SUCRA, the priorities of combined treatments, including benzodiazepines combined with CBT-I, acupuncture combined with benzodiazepines, and benzodiazepines combined with exercise, were generally confirmed. Moreover, pair-wise meta-analyses of PSQI total score and polysomnography data also confirmed the superiority of combined treatments for sleep quality and sleep architecture, respectively.

The other notable result was the comparative effectiveness of CIM approaches on elderly individuals with insomnia. Especially in the case of acupuncture, it was an efficient adjuvant strategy for benzodiazepines to improve their effectiveness. None of the included studies used acupuncture combined with CBT-I. However, some previously published studies suggest that CBT-I, known as the first-line treatment for insomnia, and acupuncture may have different therapeutic characteristics. These studies have found that acupuncture showed weaker effects of improving insomnia itself compared to CBT-I, but showed an excellent effect in improving accompanying conditions, especially pain and pain-related insomnia (73–76). Although acupuncture may still need more solid evidence to be recommended for routine treatment of elderly individuals with insomnia (77), the treatment seems to be useful as an adjuvant strategy to complement conventional treatments. Given that benzodiazepines should be used very carefully in the elderly (18), these drugs in combination with acupuncture may increase the effectiveness. This interaction could possibly reduce the dose of benzodiazepines. Also, given the high prevalence of pain in the elderly (78), acupuncture may have the potential to improve both pain-related insomnia and pain condition in this population.

Another interesting finding is that BT was ranked the most effective in the SUCRA of PSQI total score. According to the results of NMA, BT was significantly superior to wait-list as well as sleep education and relaxation in improving PSQI total score and tended to be superior to benzodiazepines and CBT with borderline significance. This finding was based on the results of a 4-week RCT comparing multicomponent behavioral treatment and sleep education (47). Two other studies (45, 58) also used multicomponent behavioral treatments, but they were not included in this analysis because they did not report the PSQI total score. Buysse et al. (47) described the BT, brief behavioral treatment for insomnia (BBTI), which focuses on behavioral elements of insomnia treatment rather than cognitive components compared to CBT-I. They also explain that because CBT-I is limited by the number of specialty-trained clinicians and by its duration or cost of treatment, a simpler and more acceptable BBTI can be more efficient and effective. Although the PSQI total score was not reported, McCrae et al. (45) also found that the BT group showed significant improvements compared to the sleep education group in sleep diary-measured SOL (*p* < 0.01) and sleep efficiency (*p* < 0.01), after 4 weeks of treatment. Moreover, Chan et al. (58), which used BBTI for 4 weeks, found that the BT group showed significant improvements compared to the attention control group in sleep diary-measured sleep variability outcomes including sleep efficiency (*p* < 0.01) and TST (*p* = 0.03), and actigraphy-measured sleep variability outcomes including SOL (*p* = 0.01) and sleep efficiency (*p* = 0.03). Although there is still little evidence to conclude, BT, which removes cognitive components from CBT-I and emphasizes behavioral elements, is worth comparing to CBT-I, which is considered as the first-line treatment of elderly individuals with insomnia. In particular, in older people with cognitive impairments such as dementia, BT with less cognitive components may be more effective, but this is still a hypothesized effect. It is expected that further studies will be conducted to compare the effectiveness and acceptability of BT and CBT-I according to the characteristics of patients with insomnia. Regarding cognitive impairments, although not included in the outcomes of interest, one of the included studies reported changes in cognitive function using the Montreal Cognitive Assessment (MoCA) (68). In this study (68), acupuncture for 4 weeks was associated with significantly improved total MoCA scores as well as spatiotemporal/executive ability, attention, and delayed memory compared to estazolam (all *p* < 0.05). However, since only one study reported changes in cognitive function, the reliability of the findings was low.

Lastly, the difference between BT and sleep education in the pair-wise meta-analysis of polysomnography data should be pointed out. The results are based on one RCT (47) with 4 weeks of treatment and 3 months of follow-up. After treatment duration (at the fourth week), compared with the sleep education group, the BT group showed better results in WASO and sleep efficiency, but showed significantly inferior results in SOL and TST. Buysse et al. (47) interpreted these results as being influenced by the initial sleep restriction. In other words, due to the initial strict sleep restriction, the TST temporarily decreased while sleep efficiency increased. As this sleep restriction was relaxed, the SOL and TST of the BT group were improved at the 6-month follow-up.

Based on the effectiveness, acceptability, and safety data found in this review, when treating elderly individuals with insomnia in clinical practice, it may be helpful to combine two or more treatments, and individual treatment strategies can be established based on the patient's preferences and accompanying symptoms. For example, acupuncture may be an important treatment component for patients with insomnia and pain or with poor cognitive status. CBT-I may be difficult to apply to these individuals. Moreover, BT without the cognitive component may also be an alternative in elderly individuals with insomnia who suffer from such cognitive difficulties. However, since the treatment may reduce TST in a short period of 4 weeks or fewer, it is necessary to consider other strategies or provide sufficient explanations before treatment in cases where compliance is a concern. Although not found in our review, adherence to treatments in elderly patients may be related to factors such as disease-related knowledge, health literacy, cognitive function (79), and frailty syndrome (80). Therefore, compliance with non-pharmacological treatment, pharmacological treatment, or combined treatment strategies in elderly patients with insomnia require further investigation.

### Strengths and Limitations

NMA is a valuable meta-analysis method that allows the selection of the most efficient options among several treatment options. Although non-pharmacological treatments are very important for elderly individuals with insomnia owing to the limited availability of pharmacotherapy in comparison to adults with insomnia (18), to the best of our knowledge, no attempt has been made to analyze the comparative effectiveness of the different non-pharmacological treatments available, until recently. This review has the advantage of using NMA methodology to derive the comparative advantage of several non-pharmacological treatments in terms of effectiveness, acceptability, and safety in elderly individuals with insomnia based on current evidence. The results can help clinicians, patients, and policymakers to make informed decisions as to the optimal non-pharmacological treatments for the treatment of insomnia in the elderly.

However, several limitations should be pointed out. First, the number of RCTs included is small compared to the interventions covered in this review. This leads to the limitation that most of the results, especially in pair-wise meta-analysis results, are based on one or two RCTs. This may indicate a lack of relevant trials on this issue. Indeed, the issues of “older adults” and “non-pharmacological treatments” seem to have received less attention in research compared with “pharmacological treatments” (81–83). Elderly individuals with insomnia, however, carry huge medical and social burdens (10–16). It is therefore urgent to support clinical trials of non-pharmacological treatments for elderly individuals with insomnia at the social and/or national levels. Second, unlike the protocol in this study, SMD, rather than the mean difference (MD), was used for continuous outcomes. This is because the consistency model between some comparisons was not established in the inconsistency test of the PSQI global score. Instead, SMD was used. Here, a consistency model was established between all comparisons. In addition, in the meta-analysis, SMD has a generalizability advantage over MD, so it may be a better unit for this review (31). Third, the various methods of acupuncture were not considered in the analysis of this review. This review found that acupuncture may be a promising adjuvant for elderly individuals with insomnia. However, different methods of acupuncture can also have different effects on insomnia. For example, a recent NMA with Bayesian analysis analyzed 52 RCTs and concluded that scale acupuncture is most effective for treating primary insomnia (84). Therefore, in future studies, expert consensus about the most effective acupuncture methods for treating elderly individuals with insomnia in clinical settings should be derived, and acupuncture trials based on the standardized acupuncture methods should be conducted. Fourth, only nine RCTs (42, 47, 48, 52, 55, 56, 58, 62, 64) conducted follow-up and only five (42, 47, 48, 55, 62) of them reported long-term follow-up data over 6 months. Like in the case of Buysse et al. (47), the sleep improvement effect of BT may need to be observed in the long-term. Moreover, CBT-I, which corrects dysfunctional beliefs about sleep itself, may have different effects in the long-term than other non-pharmacological interventions, considering its mechanism (e.g., prevent relapse of insomnia) (85, 86). On the other hand, recent research indicated that the cognitive effects of CBT-I are not significantly associated with improvements in insomnia symptoms (87). Therefore, these issues need to be further clarified through long-term follow-up trials to determine which factors, including cognitive elements of CBT-I, affect long-term insomnia symptoms. Fifth, in terms of acceptability and safety, there were not enough cases reported in the original RCT included in this review to conclude. This may suggest that non-pharmacological treatments were generally acceptable and safe; however, it also may indicate potentially poor reporting in drop-out and safety profiles among original RCTs. Given the importance of these outcomes, especially in older people, future studies should report more stringent drop-out and AEs occurrences. Sixth, the overall quality of the RCTs included in this review was low to moderate, particularly at risk of some overestimation due to lack of blinding procedures. Due to the nature of non-pharmacological interventions, the lack of blinding of participants and personnel seems inevitable. However, the rigorous implementation of assessor blinding can be an important quality assurance procedure that addresses the problem of overestimation. Future studies should address efforts to minimize the risk of overestimation, with particular emphasis on assessor blinding. Seventh, in this review, pharmacological treatments, including benzodiazepines, were considered in assessing the relative effectiveness of non-pharmacological treatments of interest. Since this review aimed to investigate the comparative effectiveness of some non-pharmacological treatments or combination treatment strategies for elderly insomnia, the findings should not be interpreted to indicate the effectiveness and safety profile of pharmacological treatment alone. Finally, cost-effectiveness is an important area of health care, especially CBT-I, which has barriers to use due to the shortage of trained practitioners and its duration and/or cost of treatment (86). The results of this review have shown promising results for a CIM modality, acupuncture. Given that the cost-effectiveness of this treatment has been demonstrated in various clinical conditions (88–90), the cost-effectiveness of interventions, including acupuncture for elderly individuals with insomnia, should be further investigated.

## Conclusions

In terms of effectiveness in PSQI total score, compared to wait-list, acupuncture, acupuncture combined with benzodiazepines, BT, benzodiazepines, benzodiazepines combined with CBT, and CBT showed superior benefits. Importantly, combined treatments, including benzodiazepines combined with CBT or with acupuncture, were generally superior to other monotherapies. In terms of acceptability, there was not enough data to conclude. In terms of safety, there was limited evidence that acupuncture is overall safe than pharmacological interventions. However, most of the RCTs included had methodological problems, especially related to the lack of blinding procedure, suggesting the risk of overestimation of their effect size. Therefore, future studies should address efforts to minimize the risk of overestimation, with particular emphasis on the assessor blinding procedure.

## Data Availability Statement

The original contributions presented in the study are included in the article/Supplementary Materials, further inquiries can be directed to the corresponding author/s.

## Author Contributions

The study was conceptualized by C-YK. C-YK and BL searched and selected the trials, and extracted, analyzed, and interpreted the data. C-YK drafted the manuscript. MC, T-HK, B-HJ, SC, and JK helped with the study design and critically reviewed the manuscript. All authors read and approved the final version of the manuscript.

## Conflict of Interest

The authors declare that the research was conducted in the absence of any commercial or financial relationships that could be construed as a potential conflict of interest.

## Abbreviations

AE: adverse event
AIS: Athens Insomnia Scale
AMED: the Allied and Complementary Medicine Database
AUC: area under the curve
BBTI: brief behavioral treatment for insomnia
BT: behavioral treatment
CBT: cognitive behavioral therapy
CBT-I: cognitive-behavioral therapy for insomnia
CCMD: the Chinese Classification of Mental Disorders
CENTRAL: the Cochrane Central Register of Controlled Trials
CI: confidence interval
CIM: complementary and integrative medicine
CINAHL: the Cumulative Index to Nursing and Allied Health Literature
CNKI: China National Knowledge Infrastructure
CPG: clinical practice guideline
*DSM*: the *Diagnostic and Statistical Manual of Mental Disorders*
GDS: the Geriatric Depression Scale
GRADE: the Grading of Recommendations Assessment, Development and Evaluation
HAMA: the Hamilton Anxiety Rating Scale
HAMD: the Hamilton Depression Rating Scale
ICD: the International Statistical Classification of Diseases and Related Health Problems
ICSD: the International Classification of Sleep Disorders
IRB: the Institutional Review Board
ISI: the Insomnia Severity Index
KCI: Korea Citation Index
KISS: Koreanstudies Information Service System
KMbase: Korean Medical Database
LSEQ: the Leeds Sleep Evaluation Questionnaire
MD: mean difference
MoCA: Montreal cognitive assessment
NMA: network meta-analysis
OASIS: Oriental Medicine Advanced Searching Integrated System
OR: odds ratio
PRISMA: the Preferred Reporting Items for Systematic Reviews and Meta-Analyses
PSQI: the Pittsburgh Sleep Quality Index
RCT: randomized controlled trial
RISS: Research Information Service System
SF-36: the 36-Item Short Form Survey
SMD: standardized mean difference
SOL: sleep onset latency
SSRI: selective serotonin reuptake inhibitor
SUCRA: surface under the cumulative ranking probabilities
TER: total effective rate
TESS: the Treatment Emergent Symptom Scale
TST: total sleep time
WASO: wake time after sleep onset.

## References

[1] Sateia MJ Buysse DJ Krystal AD Neubauer DN Heald JL. Clinical practice guideline for the pharmacologic treatment of chronic insomnia in adults: an American academy of sleep medicine clinical practice guideline. J Clin Sleep Med. (2017) 13:307–49. 10.5664/jcsm.6470 27998379 PMC5263087

[2] Roth T Coulouvrat C Hajak G Lakoma MD Sampson NA Shahly V. Prevalence and perceived health associated with insomnia based on DSM-IV-TR; international statistical classification of diseases and related health problems, tenth revision; and research diagnostic criteria/international classification of sleep disorders, second edition criteria: results from the America insomnia survey. Biol Psychiatry. (2011) 69:592–600. 10.1016/j.biopsych.2010.10.023 21195389

[3] Kim KW Kang SH Yoon IY Lee SD Ju G Han JW. Prevalence and clinical characteristics of insomnia and its subtypes in the Korean elderly. Archi Gerontol Geriatr. (2017) 68:68–75. 10.1016/j.archger.2016.09.005 27665575

[4] Zou Y Chen Y Yu W Chen T Tian Q Tu Q. The prevalence and clinical risk factors of insomnia in the Chinese elderly based on comprehensive geriatric assessment in Chongqing population. Psychogeriatrics. (2019) 19:384–90. 10.1111/psyg.12402 30739358

[5] Singareddy R Vgontzas AN Fernandez-Mendoza J Liao D Calhoun S Shaffer ML. Risk factors for incident chronic insomnia: a general population prospective study. Psychogeriatrics. (2012) 13:346–53. 10.1016/j.sleep.2011.10.033 22425576 PMC3319648

[6] Qaseem A Kansagara D Forciea MA Cooke M Denberg TD. Management of chronic insomnia disorder in adults: a clinical practice guideline from the American college of physicians. Ann Intern Med. (2016) 165:125–33. 10.7326/M15-2175 27136449

[7] Riemann D Baglioni C Bassetti C Bjorvatn B. European guideline for the diagnosis and treatment of insomnia. J Sleep Res. (2017) 26:675–700. 10.1111/jsr.12594 28875581

[8] Rios P Cardoso R Morra D Nincic V Goodarzi Z Farah B. Comparative effectiveness and safety of pharmacological and non-pharmacological interventions for insomnia: an overview of reviews. Syst Rev. (2019) 8:281. 10.1186/s13643-019-1163-9 31730011 PMC6857325

[9] Foley DJ Monjan AA Brown SL Simonsick EM Wallace RB Blazer DG. Sleep complaints among elderly persons: an epidemiologic study of three communities. Sleep. (1995) 18:425–32. 10.1093/sleep/18.6.425 7481413

[10] Haimov I. Association between memory impairment and insomnia among older adults. Euro J Ageing. (2006) 3:107. 10.1007/s10433-006-0026-0 28794756 PMC5546261

[11] de Almondes KM Costa MV Malloy-Diniz LF Diniz BS. Insomnia and risk of dementia in older adults: systematic review and meta-analysis. J Psychiatric Res. (2016) 77:109–15. 10.1016/j.jpsychires.2016.02.021 27017287

[12] Li L Wu C Gan Y Qu X Lu Z. Insomnia and the risk of depression: a meta-analysis of prospective cohort studies. BMC Psychiatry. (2016) 16:375. 10.1186/s12888-016-1075-3 27816065 PMC5097837

[13] Hsu CY Chen YT Chen MH Huang CC Chiang CH Huang PH. The association between insomnia and increased future cardiovascular events: a nationwide population-based study. Psychosom Med. (2015) 77:743–51. 10.1097/PSY.0000000000000199 26355726

[14] He Q Zhang P Li G Dai H Shi J. The association between insomnia symptoms and risk of cardio-cerebral vascular events: a meta-analysis of prospective cohort studies. Euro J Prev Cardiol. (2017) 24:1071–82. 10.1177/2047487317702043 28359160

[15] Wang YM Song M Wang R Shi L He J Fan TT. Insomnia and multimorbidity in the community elderly in China. J Clin Sleep Med. (2017) 13:591–7. 10.5664/jcsm.6550 28212690 PMC5359336

[16] Parthasarathy S Vasquez MM Halonen M Bootzin R Quan SF Martinez FD. Persistent insomnia is associated with mortality risk. Am J Med. (2015) 128:268–75.e2. 10.1016/j.amjmed.2014.10.015 25447616 PMC4340773

[17] McMillan JM Aitken E Holroyd-Leduc JM. Management of insomnia and long-term use of sedative-hypnotic drugs in older patients. CMAJ. (2013) 185:1499–505. 10.1503/cmaj.130025 24062170 PMC3832558

[18] By the American Geriatrics Society 2015 Beers Criteria Update Expert Panel American geriatrics society 2015 updated beers criteria for potentially inappropriate medication use in older adults. J Am Geriatr Soc. (2015) 63:2227–46. 10.1111/jgs.13702 26446832

[19] Lam S Macina LO. Therapy Update for Insomnia in the Elderly. Consult Pharm. (2017) 32:610–22. 10.4140/TCP.n.2017.610 28992822

[20] Sadler P McLaren S Klein B Harvey J Jenkins M. Cognitive behavior therapy for older adults with insomnia and depression: a randomized controlled trial in community mental health services. Sleep. (2018) 41: 1–12. 10.1093/sleep/zsy104 29800468

[21] Bloom HG Ahmed I Alessi CA Ancoli-Israel S Buysse DJ Kryger MH. Evidence-based recommendations for the assessment and management of sleep disorders in older persons. J Am Geriatr Soc. (2009) 57:761–89. 10.1111/j.1532-5415.2009.02220.x 19484833 PMC2748127

[22] Kanters S Ford N Druyts E Thorlund K Mills EJ Bansback N. Use of network meta-analysis in clinical guidelines. Bull World Health Organ. (2016) 94:782–4. 10.2471/BLT.16.174326 27843171 PMC5043215

[23] National Institute for Health and Care Excellence The guidelines manual: London: National Institute for Health and Care Excellence (2012).

[24] World Health Organization Consolidated Guidelines on HIV Prevention, Diagnosis, Treatment and Care for Key Populations. Geneva: WHO (2016). 27559558

[25] Hutton B Salanti G Caldwell DM Chaimani A Schmid CH Cameron C. The PRISMA extension statement for reporting of systematic reviews incorporating network meta-analyses of health care interventions: checklist and explanations. Ann Intern Med. (2015) 162:777–84. 10.7326/M14-2385 26030634

[26] Edition F Diagnostic and Statistical Manual of Mental Disorders. Philadelphia, PA: American Psychiatric Association (2013).

[27] World Health Organization International Statistical Classification of Diseases and Related Health Problems: Instruction Manual. Geneva: World Health Organization (2004).

[28] Sateia MJ. International classification of sleep disorders. Chest. (2014) 146:1387–94. 10.1378/chest.14-0970 25367475

[29] Chen YF. Chinese classification of mental disorders (CCMD-3): towards integration in international classification. Psychopathology. (2002) 35:171–5. 10.1159/000065140 12145505

[30] De Crescenzo F Foti F Ciabattini M Del Giovane C Watanabe N Sañé Schepisi M Comparative efficacy and acceptability of pharmacological treatments for insomnia in adults: a systematic review and network meta-analysis. Cochrane Database System Rev. (2016) 2016:CD012364 10.1002/14651858.CD012364

[31] Buysse DJ Reynolds CF III Monk TH Berman SR Kupfer DJ. The pittsburgh sleep quality index: a new instrument for psychiatric practice and research. Psychiatry Res. (1989) 28:193–213. 10.1016/0165-1781(89)90047-4 2748771

[32] Bastien CH Vallières A Morin CM. Validation of the insomnia severity index as an outcome measure for insomnia research. Sleep Med. (2001) 2:297–307. 10.1016/S1389-9457(00)00065-4 11438246

[33] Parrott AC Hindmarch I. The leeds sleep evaluation questionnaire in psychopharmacological investigations - a review. Psychopharmacology. (1980) 71:173–9. 10.1007/BF00434408 6777817

[34] Health NIoM TESS (treatment emergent symptom scale-write-in). Psychopharmacol Bull. (1985) 21:1069–72.

[35] Higgins J Altman D Sterne J Chapter 8: assessing risk of bias in included studies. In: Cochrane Handbook for Systematic Reviews of Interventions Version 5.1. Nanning: The Cochrane Collaboration (2011).

[36] Jadad AR Moore RA Carroll D Jenkinson C Reynolds DJ Gavaghan DJ. Assessing the quality of reports of randomized clinical trials: is blinding necessary? Control Clin Trials. (1996) 17:1–12. 10.1016/0197-2456(95)00134-4 8721797

[37] Higgins J Green S Identifying and measuring heterogeneity. In: Cochrane Handbook for Systematic Reviews of Interventions Version. London: The Cochrane Collaboration (2008). p. 510.

[38] Borenstein M Hedges LV Higgins JP Rothstein HR. A basic introduction to fixed-effect and random-effects models for meta-analysis. Res Synth Methods. (2010) 1:97–111. 10.1002/jrsm.12 26061376

[39] Murad M Montori V Ioannidis J Guyatt G Rennie D Meade M. Fixed effects and random-effects models. In: Guyatt G Rennie D Meade MO Cook DJ editors. Users' Guide to the Medical Literature: A Manual for Evidence-Based Clinical Practice McGraw-Hill, 3rd ed. New York, NY: McGraw-Hill Education (2015).

[40] Shim S Yoon BH. Network meta-analysis: application and practice using stata. (2017) 39:e2017047. 10.4178/epih.e2017047 29092392 PMC5733388

[41] Puhan MA Schünemann HJ Murad MH Li T Brignardello-Petersen R Singh JA A GRADE Working Group approach for rating the quality of treatment effect estimates from network meta-analysis. BMJ. (2014) 349:g5630 10.1136/bmj.g5630 25252733

[42] Morin CM Kowatch RA Barry T Walton E. Cognitive-behavior therapy for late-life insomnia. J Consult Clin Psychol. (1993) 61:137–46. 10.1037/0022-006X.61.1.137 8450099

[43] Song LZ Li H Zhang H Zhang XR Acupuncture combined with relaxation therapy for the treatment of senile sleep disorders. Liaoning J Tradit Chin Med. (2005) 32:359–60.

[44] Yang YJ Fei CL Zhang JH. Effect of nursing intervention on sleep quality of elderly patients with insomnia. Nurs J Chin Peopl Liberat Army. (2006) 23:14–6. 32810953

[45] McCrae CS McGovern R Lukefahr R Stripling AM. Research evaluating brief behavioral sleep treatments for rural elderly (RESTORE): a preliminary examination of effectiveness. Am J Geriatr Psychiatry. (2007) 15:979–82. 10.1097/JGP.0b013e31813547e6 17974868

[46] Weng M Liao HQ Analysis of the therapeutic effect of electroacupuncture on senile insomnia. J Clin Acupunct Moxibust. (2007) 23:33–4.

[47] Buysse DJ Germain A Moul DE Franzen PL Brar LK Fletcher ME. Efficacy of brief behavioral treatment for chronic insomnia in older adults. Arch Intern Med. (2011) 171:887–95. 10.1001/archinternmed.2010.535 21263078 PMC3101289

[48] Lin JZ Zhang ML Ou LM Wang CJ Ye RF Long-term effect study of acupuncture therapy combining with biofeedback relaxation therapy on senile chronic insomnia patients. CJTCMP. (2012) 27:2222–4.

[49] Zhu L Wu W Yu E Zhou J Zhou H Aerobic exercise on the clinical efficacy of elderly patients with insomnia. Zhejiang Med J. (2012) 34:177–8.e95.

[50] Liu Y. Evaluation of the effect of acupoint massage on the head for improving insomnia in the elderly. J Chin Phys. (2014) 274-6. 16813176

[51] Ren LN Li X Acupuncture and moxibustion for the treatment of senile insomnia. J Taishan Med Colle. (2014) 35:660–1.

[52] Wang J Wang J Wang L Zhang Y. Senile insomnia treated with integrated acupuncture and medication therapy: a randomized controlled trial. Chin Acupunct Moxibust. (2015) 35:544–8. 26480547

[53] Zhang JX Liu XH Xie XH Zhao D Shan MS Zhang XL. Mindfulness-based stress reduction for chronic insomnia in adults older than 75 years: a randomized, controlled, single-blind clinical trial. Explore. (2015) 11:180–5. 10.1016/j.explore.2015.02.005 25843539

[54] Xu P Ji WD Pan YS Effects of cognitive behavioral therapy combined with drug on serum cytokines and cortisol in the elderly patients with sleep disorders. Pract Geriatr. (2015) 29:137–41.

[55] Alessi C Martin JL Fiorentino L Fung CH Dzierzewski JM Rodriguez Tapia JC. Cognitive behavioral therapy for insomnia in older veterans using nonclinician sleep coaches: Randomized controlled trial. J Am Geriatr Soc. (2016) 64:1830–8. 10.1111/jgs.14304 27550552 PMC5351772

[56] Mottaghi R Kamkar A Maredpoor A Effectiveness of cognitive behavior therapy on the quality of sleep in elderly people with insomnia disorder. Iran J Ageing. (2016) 11:234–43. 10.21859/sija-1102234

[57] Duan SD Preliminary observation and evaluation of acupuncture treatment of insomnia in the elderly. Good Health for All. (2016) 10:39.

[58] Chan WS Williams J Dautovich ND McNamara JPH Stripling A Dzierzewski JM Night-to-night sleep variability in older adults with chronic insomnia: mediators and moderators in a randomized controlled trial of brief behavioral therapy (BBT-I). J Clin Sleep Med. (2017) 13:1243–54. 10.5664/jcsm.6790 28992829 PMC5656473

[59] Liang XM Clinical observations on the therapeutic effect of ear acupoint thumbtack needle embedding on senile primary insomnia. Shanghai J Acu-mox. (2017) 36:719–22.

[60] Lin Q Mo Y Sun S Clinical observation on 90 cases of senile insomnia treated with auricular point sticking. Aging Res. (2017) 4:1–5. 10.12677/AR.2017.41001

[61] Xue WX Zhang JY Ge LL Effect of acupuncture at five shu points on sleep quality of elderly patients with neurological insomnia. Chin J Gerontology. (2017) 37:5390–2.

[62] Zhang Y Clinical effect analysis of cognitive behavioral therapy (CBT-I) combined with estazolam in the treatment of elderly patients with chronic severe insomnia. Electro J Clin Med Literat. (2017) 4:13937–8.

[63] Chen P Luo W Qi L Tang W Clinical effect of hilum therapy combined with acupuncture in the treatment of senile insomnia. J Hunan Univ Chin Med. (2017) 37:1013–6.

[64] Mo JJ Clinical Research of “Sancai Acupoints Combination” for Senile Primary Nsomnia. (Beijing:Master's degree), Guangxi Traditional Chinese Medical University (2018).

[65] Yuan F Zhao X Huang Y Luo B Clinical evaluation on guipi decoction combined with acupuncture in the treatment of elderly patients with insomnia (syndrome of deficiency of both qi and blood). China Pharmaceuticals. (2018) 27:34–7.

[66] Xu Y Wang R Yang Z Guo J Cognitive-behavioral therapy and acupuncture therapy in the treatment of senile chronic insomnia. Med J Chin PAP. (2018) 29:1125–8.

[67] Liu L Evaluation of short-term curative effect of acupuncture on senile insomnia. Home Med. (2019):68.

[68] Yu XP Gao QC Clinical study on the effect of acupuncture on sleep quality and cognitive function in elderly patients with primary insomnia. Jiangsu J Tradit Chin Med. (2019) 51:62–4.

[69] Wei DL Study on the Intervention of Ba Duan Jin Combined with Cognitive Behavioral Therapy in Elderly Insomnia. (Master's degree). Beijing: Beijing University of Chinese Medicine (2019).

[70] Davies J Rae TC Montagu L. Long-term benzodiazepine and Z-drugs use in England: a survey of general practice [corrected]. Br J Gener Pract. (2017) 67:e609–13. 10.3399/bjgp17X691865 28716996 PMC5569740

[71] Markota M Rummans TA Bostwick JM Lapid MI. Benzodiazepine Use in older adults: dangers, management, and alternative therapies. Mayo Clinic proceedings. (2016) 91:1632–9. 10.1016/j.mayocp.2016.07.024 27814838

[72] Sys J Van Cleynenbreugel S Deschodt M Van der Linden L Tournoy J. Efficacy and safety of non-benzodiazepine and non-Z-drug hypnotic medication for insomnia in older people: a systematic literature review. Euro J Clin Pharmacol. (2020) 76:363–81. 10.1007/s00228-019-02812-z 31838549

[73] Ganguly G. Acupuncture may be helpful only for patients with comorbid insomnia secondary to chronic pain syndromes. Evid Based Complement Alternat Med. (2011) 7:411. 10.5664/JCSM.1208 21897781 PMC3161776

[74] Bergdahl L Broman JE Berman AH Haglund K von Knorring L Markstrom A. Auricular acupuncture and cognitive behavioural therapy for insomnia: a randomised controlled study. Epidemiology Health. (2016) 2016:7057282. 10.1155/2016/7057282 27242930 PMC4876000

[75] Liu F You J Li Q Fang T. Acupuncture for chronic pain-related insomnia: a systematic review and meta-analysis. Evid Based Complement Alternat Med. (2019) 2019:5381028. 10.1155/2019/5381028 31341495 PMC6612974

[76] Romero SAD Jiang E Bussell J Eriksen W Duhamel KN Barg FK. What makes one respond to acupuncture for insomnia? Perspectives of cancer survivors. Sleep Disord. (2019) 18:1–6. 10.1017/S1478951519000762 31571560 PMC7108961

[77] Cheuk DK Yeung WF Chung KF Wong V Acupuncture for insomnia. Cochrane Database Syst Rev. (2012) Cd005472. 10.1002/14651858.CD005472.pub3 17636800

[78] Patel KV Guralnik JM Dansie EJ Turk DC. Prevalence and impact of pain among older adults in the United States: findings from the 2011 national health and aging trends study. Pain. (2013) 154:2649–57. 10.1016/j.pain.2013.07.029 24287107 PMC3843850

[79] Gellad WF Grenard JL Marcum ZA. A systematic review of barriers to medication adherence in the elderly: looking beyond cost and regimen complexity. Am J Geriatr Pharmacother. (2011) 9:11–23. 10.1016/j.amjopharm.2011.02.004 21459305 PMC3084587

[80] Jankowska-Polańska B Zameta K Uchmanowicz I Szymańska-Chabowska A Morisky D Mazur G. Adherence to pharmacological and non-pharmacological treatment of frail hypertensive patients. J Geriatr Cardiol. (2018) 15:153–61. 10.11909/j.issn.1671-5411.2018.02.002 29662509 PMC5895955

[81] Knechel NA. The challenges of enrolling older adults into intervention studies. Yale J Biol Med. (2013) 86:41–7. 23482244 PMC3584494

[82] Shenoy P Harugeri A. Elderly patients' participation in clinical trials. Perspect Clin Res. (2015) 6:184–9. 10.4103/2229-3485.167099 26623388 PMC4640010

[83] Cristea IA Gentili C Pietrini P Cuijpers P. Sponsorship bias in the comparative efficacy of psychotherapy and pharmacotherapy for adult depression: meta-analysis. Br J Psychiatry. (2017) 210:16–23. 10.1192/bjp.bp.115.179275 27810891

[84] Xu H Shi Y. Efficacy comparison of different acupuncture treatments for primary insomnia: a bayesian analysis. Evid Based Complement Alternat Med. (2019) 2019:8961748. 10.1155/2019/8961748 31565065 PMC6745175

[85] Morin CM Belanger L Bastien C Vallieres A. Long-term outcome after discontinuation of benzodiazepines for insomnia: a survival analysis of relapse. Behav Res Ther. (2005) 43:1–14. 10.1016/j.brat.2003.12.002 15531349

[86] Rossman J. Cognitive-behavioral therapy for insomnia: an effective and underutilized treatment for insomnia. Am J Lifestyle Med. (2019) 13:544–7. 10.1177/1559827619867677 31662718 PMC6796223

[87] Okajima I Nakajima S Ochi M Inoue Y Reducing dysfunctional beliefs about sleep does not significantly improve insomnia in cognitive behavioral therapy. Evid Based Complement Alternat Med. (2014) 9:e102565 10.1371/journal.pone.0102565 PMC4099188 25025164

[88] Spackman E Richmond S Sculpher M Bland M Brealey S Gabe R. Cost-effectiveness analysis of acupuncture, counselling and usual care in treating patients with depression: the results of the ACUDep trial. PloS ONE. (2014) 9:e113726. 10.1371/journal.pone.0113726 25426637 PMC4245224

[89] Taylor P Pezzullo L Grant SJ Bensoussan A. Cost-effectiveness of acupuncture for chronic nonspecific low back pain. Pain Pract. (2014) 14:599–606. 10.1111/papr.12116 24138020

[90] Witt CM Reinhold T Jena S Brinkhaus B Willich SN. Cost-effectiveness of acupuncture in women and men with allergic rhinitis: a randomized controlled study in usual care. Am J Epidemiol. (2009) 169:562–71. 10.1093/aje/kwn370 19126587

