# Supplementary material for: Non-pharmacological Treatment for Elderly Individuals With Insomnia: A Systematic Review and Network Meta-Analysis
Source: Front Psychiatry. 2021 Jan 28;11:608896. doi: 10.3389/fpsyt.2020.608896 (PMC7876437; doi:10.3389/fpsyt.2020.608896)
Supplement: Supplementary file 1 [file Data_Sheet_1.docx]

**Supplementary Material 1. PRISMA NMA checklist**

**Table S1. PRISMA NMA checklist**

| **Section/Topic** | **Item #** | **Checklist Item** | **Reported on Page #** |
| --- | --- | --- | --- |
| **TITLE** |  |  |  |
| Title | 1 | Identify the report as a systematic review *incorporating a network meta-analysis (or related form of meta-analysis).* | Title |
| **ABSTRACT** |  |  |  |
| Structured summary | 2 | Provide a structured summary including, as applicable:  **Background:** main objectives  **Methods:** data sources; study eligibility criteria, participants, and interventions; study appraisal; and *synthesis methods, such as network meta-analysis.*  **Results:** number of studies and participants identified; summary estimates with corresponding confidence/credible intervals; *treatment rankings may also be discussed. Authors may choose to summarize pairwise comparisons against a chosen treatment included in their analyses for brevity.*  **Discussion/Conclusions:** limitations; conclusions and implications of findings.  **Other:** primary source of funding; systematic review registration number with registry name. | 2 |
| **INTRODUCTION** |  |  |  |
| Rationale | 3 | Describe the rationale for the review in the context of what is already known*, including mention of why a network meta-analysis has been conducted.* | 4-5 |
| Objectives | 4 | Provide an explicit statement of questions being addressed, with reference to participants, interventions, comparisons, outcomes, and study design (PICOS). | 5 |
| **METHODS** |  |  |  |
| Protocol and registration | 5 | Indicate whether a review protocol exists and if and where it can be accessed (e.g., Web address); and, if available, provide registration information, including registration number. | 6 |
| Eligibility criteria | 6 | Specify study characteristics (e.g., PICOS, length of follow-up) and report characteristics (e.g., years considered, language, publication status) used as criteria for eligibility, giving rationale. *Clearly describe eligible treatments included in the treatment network, and note whether any have been clustered or merged into the same node (with justification).* | 6-7 |
| Information sources | 7 | Describe all information sources (e.g., databases with dates of coverage, contact with study authors to identify additional studies) in the search and date last searched. | 6 |
| Search | 8 | Present full electronic search strategy for at least one database, including any limits used, such that it could be repeated. | 6, Appendix 2 |
| Study selection | 9 | State the process for selecting studies (i.e., screening, eligibility, included in systematic review, and, if applicable, included in the meta-analysis). | 8-9 |
| Data collection process | 10 | Describe method of data extraction from reports (e.g., piloted forms, independently, in duplicate) and any processes for obtaining and confirming data from investigators. | 8-9 |
| Data items | 11 | List and define all variables for which data were sought (e.g., PICOS, funding sources) and any assumptions and simplifications made. | 8-9 |
| **Geometry of the network** | **S1** | Describe methods used to explore the geometry of the treatment network under study and potential biases related to it. This should include how the evidence base has been graphically summarized for presentation, and what characteristics were compiled and used to describe the evidence base to readers. | None |
| Risk of bias within individual studies | 12 | Describe methods used for assessing risk of bias of individual studies (including specification of whether this was done at the study or outcome level), and how this information is to be used in any data synthesis. | 9 |
| Summary measures | 13 | State the principal summary measures (e.g., risk ratio, difference in means). *Also describe the use of additional summary measures assessed, such as treatment rankings and surface under the cumulative ranking curve (SUCRA) values, as well as modified approaches used to present summary findings from meta-analyses.* | 9-10 |
| Planned methods of analysis | 14 | Describe the methods of handling data and combining results of studies for each network meta-analysis. This should include, but not be limited to:   - *Handling of multi-arm trials;* - *Selection of variance structure;* - *Selection of prior distributions in Bayesian analyses; and* - *Assessment of model fit.* | 9-10 |
| **Assessment of Inconsistency** | **S2** | Describe the statistical methods used to evaluate the agreement of direct and indirect evidence in the treatment network(s) studied. Describe efforts taken to address its presence when found. | 9-10 |
| Risk of bias across studies | 15 | Specify any assessment of risk of bias that may affect the cumulative evidence (e.g., publication bias, selective reporting within studies). | 11 |
| Additional analyses | 16 | Describe methods of additional analyses if done, indicating which were pre-specified. This may include, but not be limited to, the following:   - Sensitivity or subgroup analyses; - Meta-regression analyses; - *Alternative formulations of the treatment network; and* - *Use of alternative prior distributions for Bayesian analyses (if applicable).* | 10-11 |
| **RESULTS†** |  |  |  |
| Study selection | 17 | Give numbers of studies screened, assessed for eligibility, and included in the review, with reasons for exclusions at each stage, ideally with a flow diagram. | 12, Figure 1 |
| **Presentation of network structure** | **S3** | Provide a network graph of the included studies to enable visualization of the geometry of the treatment network. | Figure 2 |
| **Summary of network geometry** | **S4** | Provide a brief overview of characteristics of the treatment network. This may include commentary on the abundance of trials and randomized patients for the different interventions and pairwise comparisons in the network, gaps of evidence in the treatment network, and potential biases reflected by the network structure. | None |
| Study characteristics | 18 | For each study, present characteristics for which data were extracted (e.g., study size, PICOS, follow-up period) and provide the citations. | 12, Table 1 |
| Risk of bias within studies | 19 | Present data on risk of bias of each study and, if available, any outcome level assessment. | 12-13, Appendix 3 |
| Results of individual studies | 20 | For all outcomes considered (benefits or harms), present, for each study: 1) simple summary data for each intervention group, and 2) effect estimates and confidence intervals. *Modified approaches may be needed to deal with information from larger networks.* | None |
| Synthesis of results | 21 | Present results of each meta-analysis done, including confidence/credible intervals. *In larger networks, authors may focus on comparisons versus a particular comparator (e.g. placebo or standard care), with full findings presented in an appendix. League tables and forest plots may be considered to summarize pairwise comparisons.* If additional summary measures were explored (such as treatment rankings), these should also be presented. | 14-18 |
| **Exploration for inconsistency** | **S5** | Describe results from investigations of inconsistency. This may include such information as measures of model fit to compare consistency and inconsistency models, *P* values from statistical tests, or summary of inconsistency estimates from different parts of the treatment network. | 14-18 |
| Risk of bias across studies | 22 | Present results of any assessment of risk of bias across studies for the evidence base being studied. | 18 |
| Results of additional analyses | 23 | Give results of additional analyses, if done (e.g., sensitivity or subgroup analyses, meta-regression analyses*, alternative network geometries studied, alternative choice of prior distributions for Bayesian analyses,* and so forth). | 15 |
| **DISCUSSION** |  |  |  |
| Summary of evidence | 24 | Summarize the main findings, including the strength of evidence for each main outcome; consider their relevance to key groups (e.g., healthcare providers, users, and policy-makers). | 19-21 |
| Limitations | 25 | Discuss limitations at study and outcome level (e.g., risk of bias), and at review level (e.g., incomplete retrieval of identified research, reporting bias). *Comment on the validity of the assumptions, such as transitivity and consistency. Comment on any concerns regarding network geometry (e.g., avoidance of certain comparisons).* | 23-25 |
| Conclusions | 26 | Provide a general interpretation of the results in the context of other evidence, and implications for future research. | 25-26 |
| **FUNDING** |  |  |  |
| Funding | 27 | Describe sources of funding for the systematic review and other support (e.g., supply of data); role of funders for the systematic review. This should also include information regarding whether funding has been received from manufacturers of treatments in the network and/or whether some of the authors are content experts with professional conflicts of interest that could affect use of treatments in the network. | 26 |

**Supplementary Material 2. Search strategy used in each database**

**Table S2. Medline via PubMed**

|  | Searches | Results |
| --- | --- | --- |
| #1 | Sleep[mh] OR “Sleep Wake Disorders”[mh] OR sleep*[tiab] OR insomnia*[tiab] OR wakeful*[tiab] OR dyssomn*[tiab] | 210090 |
| #2 | Geriatrics[mh] OR Aged[mh] OR aged[tiab] OR older[tiab] OR elderly[tiab] OR senior*[tiab] OR geriatric*[tiab] | 3584025 |
| #3 | “Cognitive Behavioral Therapy”[mh] OR “cognitive behavior*”[tiab] OR “Sleep Hygiene”[mh] OR “sleep hygiene”[tiab] OR “sleep education”[tiab] OR “sleep health”[tiab] OR “sleep restriction”[tiab] OR “stimulus control”[tiab] OR Relaxation[mh] OR “Relaxation Therapy”[mh] OR “Muscle Relaxation”[mh] OR relaxation[tiab] OR Exercise[mh] OR “Exercise Therapy”[mh] OR “Exercise Movement Techniques”[mh] OR exercis*[tiab] OR “Walking”[mh] OR walking[tiab] OR “weight train*”[tiab] OR “Tai Ji”[mh] OR “Tai Chi”[tiab] OR “T’ai Chi”[tiab] OR Taiji[tiab] OR “Tai ji”[tiab] OR taijiquan[tiab] OR “Tai Chih”[tiab] OR Acupuncture[mh] OR “Acupuncture Therapy”[mh] OR Auriculotherapy[mh] OR Acupressure[mh] OR Electroacupuncture[mh] OR acupuncture[tiab] OR acupressure[tiab] OR acupoint*[tiab] OR electroacupuncture[tiab] OR electro‐acupuncture[tiab] | 642340 |
| #4 | “randomized controlled trial”[pt] OR “controlled clinical trial”[pt] OR randomized[tiab] OR placebo[tiab] OR “Clinical Trials as Topic”[mesh: noexp] OR randomly[tiab] OR trial[ti] | 1239821 |
| #5 | animals[mh] NOT humans[mh] | 4605934 |
| #6 | #1 AND #2 AND #3 AND #4 NOT #5 | **1141** |

**Table S3. EMBASE via Elsevier**

|  | Searches | Results |
| --- | --- | --- |
| #1 | ‘Sleep’/exp OR ‘sleep*’:ab,ti OR ‘sleep disorder’/exp OR ‘sleep disorder’:ab,ti OR ‘insomnia’:ab,ti OR ‘wakeful*’:ab,ti OR ‘dyssomn*’:ab,ti | 425403 |
| #2 | ‘Geriatrics’/exp OR ‘Aged’/exp OR ‘aged’:ab,ti OR ‘older’:ab,ti OR ‘elderly’:ab,ti OR ‘senior*’:ab,ti OR ‘geriatric*’:ab,ti | 3831272 |
| #3 | ‘Cognitive Behavioral Therapy’/exp OR ‘cognitive behavior*’:ab,ti OR ‘Sleep Hygiene’/exp OR ‘sleep hygiene’:ab,ti OR ‘sleep education’:ab,ti OR ‘sleep health’:ab,ti OR ‘sleep restriction’:ab,ti OR ‘stimulus control’:ab,ti | 37421 |
| #4 | ‘Relaxation Training’/exp OR ‘Muscle Relaxation’/exp OR ‘relaxation’:ab,ti OR ‘Exercise’/exp OR ‘exercis*’:ab,ti OR ‘Walking’/exp OR ‘walking’:ab,ti OR ‘weight train*’:ab,ti | 728911 |
| #5 | ‘Tai Chi’/exp OR ‘Tai Chi’:ab,ti OR ‘Taiji’:ab,ti OR ‘Tai ji’:ab,ti OR ‘taijiquan’:ab,ti OR ‘Tai Chih’:ab,ti OR ‘Acupuncture’/exp OR ‘Acupressure’/exp OR ‘acupuncture’:ab,ti OR ‘acupressure’:ab,ti OR ‘acupoint*’:ab,ti OR ‘electroacupuncture’/exp OR ‘electroacupuncture’:ab,ti OR ‘electro‐acupuncture’:ab,ti OR ‘auricular acupuncture’/exp OR ‘auricular acupressure’/exp OR ‘auricular acup*’:ab,ti OR ‘auriculotherapy’:ab,ti | 49923 |
| #6 | 'crossover procedure':de OR 'double-blind procedure':de OR 'randomized controlled trial':de OR 'single-blind procedure':de OR (random* OR factorial* OR crossover* OR cross NEXT/1 over* OR placebo* OR doubl* NEAR/1 blind* OR singl* NEAR/1 blind* OR assign* OR allocat* OR volunteer*):de,ab,ti | 2441004 |
| #7 | #1 AND #2 AND (#3 OR #4 OR #5) AND #6 | **2224** |

**Table S4. CENTRAL**

|  | Searches | Results |
| --- | --- | --- |
| #1 | MeSH descriptor: [Sleep] explode all trees | 5348 |
| #2 | MeSH descriptor: [Sleep Wake Disorders] explode all trees | 7152 |
| #3 | (sleep* OR insomnia* OR wakeful* OR dyssomn*):ti,ab,kw | 40243 |
| #4 | #1 OR #2 OR #3 | 40520 |
| #5 | MeSH descriptor: [Geriatrics] explode all trees | 198 |
| #6 | MeSH descriptor: [Aged] explode all trees | 1248 |
| #7 | (aged OR older OR elderly OR senior* OR geriatric*):ti,ab,kw | 514409 |
| #8 | #5 OR #6 OR #7 | 514409 |
| #9 | MeSH descriptor: [Cognitive Behavioral Therapy] explode all trees | 8092 |
| #10 | MeSH descriptor: [Sleep Hygiene] explode all trees | 15 |
| #11 | MeSH descriptor: [Relaxation] explode all trees | 1622 |
| #12 | MeSH descriptor: [Relaxation Therapy] explode all trees | 1808 |
| #13 | MeSH descriptor: [Muscle Relaxation] explode all trees | 1496 |
| #14 | MeSH descriptor: [Exercise] explode all trees | 22235 |
| #15 | MeSH descriptor: [Exercise Therapy] explode all trees | 12204 |
| #16 | MeSH descriptor: [Exercise Movement Techniques] explode all trees | 1890 |
| #17 | MeSH descriptor: [Walking] explode all trees | 5202 |
| #18 | MeSH descriptor: [Tai Ji] explode all trees | 321 |
| #19 | MeSH descriptor: [Acupuncture] explode all trees | 141 |
| #20 | MeSH descriptor: [Acupuncture Therapy] explode all trees | 4342 |
| #21 | MeSH descriptor: [Auriculotherapy] explode all trees | 201 |
| #22 | MeSH descriptor: [Acupressure] explode all trees | 316 |
| #23 | MeSH descriptor: [Electroacupuncture] explode all trees | 737 |
| #24 | (“cognitive behavior*” OR “sleep hygiene” OR “sleep education” OR “sleep health” OR “sleep restriction” OR “stimulus control” OR relaxation OR exercis* OR walking OR “weight train*” OR “Tai Chi” OR “T’ai Chi” OR Taiji OR “Tai ji” OR taijiquan OR “Tai Chih” OR acupuncture OR acupressure OR acupoint* OR electroacupuncture OR electro‐acupuncture):ti,ab,kw | 124231 |
| #25 | #9 OR 10 OR #11 OR #12 OR #13 OR #14 OR #15 OR #16 OR #17 OR #18 OR #19 OR #20 OR #21 OR #22 OR #23 OR #24 | 911817 |
| #26 | (“randomized controlled trial” OR "controlled clinical trial"):pt | 560971 |
| #27 | MeSH descriptor: [Clinical Trials as Topic] this term only | 33213 |
| #28 | (randomized OR placebo OR randomly OR trial):ti,ab,kw | 1070482 |
| #29 | #26 OR #27 OR #28 | 1213766 |
| #30 | MeSH descriptor: [Animals] explode all trees | 15435 |
| #31 | MeSH descriptor: [Humans] explode all trees | 8262 |
| #32 | #30 NOT #31 | 7173 |
| #31 | (#4 AND #8 AND #25 AND #29 NOT #32) in Trials | **12556** |

**Table S5. AMED via EBSCO**

|  | Searches | Results |
| --- | --- | --- |
| #1 | Sleep[SU] OR “Sleep Wake Disorders”[SU] OR sleep*[TX] OR insomnia*[TX] OR wakeful*[TX] OR dyssomn*[TX] | 3252 |
| #2 | Geriatrics[SU] OR Aged[SU] OR aged[TX] OR older[TX] OR elderly[TX] OR senior*[TX] OR geriatric*[TX] | 28740 |
| #3 | “Cognitive Behavioral Therapy”[SU] OR “cognitive behavior*”[TX] OR “Sleep Hygiene”[SU] OR “sleep hygiene”[TX] OR “sleep education”[TX] OR “sleep health”[TX] OR “sleep restriction”[TX] OR “stimulus control”[TX] OR Relaxation[SU] OR “Relaxation Therapy”[SU] OR “Muscle Relaxation”[SU] OR relaxation[TX] OR Exercise[SU] OR “Exercise Therapy”[SU] OR “Exercise Movement Techniques”[SU] OR exercis*[TX] OR “Walking”[SU] OR walking[TX] OR “weight train*”[TX] OR “Tai Ji”[SU] OR “Tai Chi”[TX] OR “T’ai Chi”[TX] OR Taiji[TX] OR “Tai ji”[TX] OR taijiquan[TX] OR “Tai Chih”[TX] OR Acupuncture[SU] OR “Acupuncture Therapy”[SU] OR Auriculotherapy[SU] OR Acupressure[SU] OR Electroacupuncture[SU] OR acupuncture[TX] OR acupressure[TX] OR acupoint*[TX] OR electroacupuncture[TX] OR electro‐acupuncture[TX] | 48255 |
| #4 | #1 AND #2 AND #3 | **134** |

**Table S6. CINAHL via EBSCO**

|  | Searches | Results |
| --- | --- | --- |
| #1 | Sleep[MH] OR “Sleep Wake Disorders”[MH] OR sleep*[TX] OR insomnia*[TX] OR wakeful*[TX] OR dyssomn*[TX] | 138426 |
| #2 | Geriatrics[MH] OR Aged[MH] OR aged[TX] OR older[TX] OR elderly[TX] OR senior*[TX] OR geriatric*[TX] | 1552869 |
| #3 | “Cognitive Behavioral Therapy”[MH] OR “cognitive behavior*”[TX] OR “Sleep Hygiene”[MH] OR “sleep hygiene”[TX] OR “sleep education”[TX] OR “sleep health”[TX] OR “sleep restriction”[TX] OR “stimulus control”[TX] OR Relaxation[MH] OR “Relaxation Therapy”[MH] OR “Muscle Relaxation”[MH] OR relaxation[TX] OR Exercise[MH] OR “Exercise Therapy”[MH] OR “Exercise Movement Techniques”[MH] OR exercis*[TX] OR “Walking”[MH] OR walking[TX] OR “weight train*”[TX] OR “Tai Ji”[MH] OR “Tai Chi”[TX] OR “T’ai Chi”[TX] OR Taiji[TX] OR “Tai ji”[TX] OR taijiquan[TX] OR “Tai Chih”[TX] OR Acupuncture[MH] OR “Acupuncture Therapy”[MH] OR Auriculotherapy[MH] OR Acupressure[MH] OR Electroacupuncture[MH] OR acupuncture[TX] OR acupressure[TX] OR acupoint*[TX] OR electroacupuncture[TX] OR electro‐acupuncture[TX] | 393380 |
| #4 | “randomized controlled trial”[PT] OR “controlled clinical trial”[PT] OR randomized[TX] OR placebo[TX] OR “Clinical Trials as Topic”[MH] OR randomly[TX] OR trial[TX] | 671522 |
| #5 | animals[MH] NOT humans[MH] | 70952 |
| #6 | #1 AND #2 AND #3 AND #4 NOT #5 | **13848** |

**Table S7. PsycARTICLES via ProQuest**

|  | Searches | Results |
| --- | --- | --- |
| #1 | Mesh(sleep OR “Sleep Wake Disorders”) OR SU(sleep* OR insomnia* OR wakeful* OR dyssomn*) | 1300 |
| #2 | Mesh(Geriatrics OR Aged) OR SU(aged OR older OR elderly OR senior* OR geriatric*) | 82888 |
| #3 | Mesh(“Cognitive Behavioral Therapy” OR “Sleep Hygiene” OR Relaxation OR “Relaxation Therapy” OR “Muscle Relaxation” OR Exercise OR “Exercise Therapy” OR “Exercise Movement Techniques” OR “Walking” OR “Tai Ji” OR Acupuncture OR “Acupuncture Therapy” OR Auriculotherapy OR Acupressure OR Electroacupuncture) OR SU(“cognitive behavior*” OR “sleep hygiene” OR “sleep education” OR “sleep health” OR “sleep restriction” OR “stimulus control” OR relaxation OR exercis* OR walking OR “weight train*” OR “Tai Chi” OR “T’ai Chi” OR Taiji OR “Tai ji” OR taijiquan OR “Tai Chih” OR acupuncture OR acupressure OR acupoint* OR electroacupuncture OR electro‐acupuncture) | 3163 |
| #4 | #1 AND #2 AND #3 | **90** |

**Table S8. OASIS**

|  | Searches | Results |
| --- | --- | --- |
| #1 | (인지행동치료 OR 수면위생 OR 수면교육 OR 수면제한 OR 자극조절) AND (불면 OR 수면) AND (노인 OR 노년) | **0** |
| #2 | (이완 OR 운동 OR 걷기 OR 태극권 OR 침) AND (불면 OR 수면) AND (노인 OR 노년) | **0** |
| #3 | (혈위 OR 지압 OR 이압) AND (불면 OR 수면) AND (노인 OR 노년) | **0** |
| #4 | #1 OR #2 OR #3 | **0** |

**Table S9. KISS**

|  | Searches | Results |
| --- | --- | --- |
| #1 | 제목: (인지행동치료 OR 수면위생 OR 수면교육 OR 수면제한 OR 자극조절) AND (불면 OR 수면) AND (노인 OR 노년) | 0 |
| #2 | 제목: (이완 OR 운동 OR 걷기 OR 태극권 OR 침) AND (불면 OR 수면) AND (노인 OR 노년) | 5 |
| #3 | 제목: (혈위 OR 지압 OR 이압) AND (불면 OR 수면) AND (노인 OR 노년) | 10 |
| #4 | #1 OR #2 OR #3 | **12** |

**Table S10. RISS**

|  | Searches | Results |
| --- | --- | --- |
| #1 | 제목: (인지행동치료 OR 수면위생 OR 수면교육) AND (불면 OR 수면) AND (노인 OR 노년) | 5 |
| #2 | 제목: (수면제한 OR 자극조절 OR 이완) AND (불면 OR 수면) AND (노인 OR 노년) | 2 |
| #3 | 제목: (운동 OR 걷기 OR 태극권) AND (불면 OR 수면) AND (노인 OR 노년) | 28 |
| #4 | 제목: (침 OR 혈위 OR 지압) AND (불면 OR 수면) AND (노인 OR 노년) | 1 |
| #5 | 제목: 이압 AND (불면 OR 수면) AND (노인 OR 노년) | 4 |
| #6 | #1 OR #2 OR #3 OR #4 OR #5 | **40** |

**Table S11. KMbase**

|  | Searches | Results |
| --- | --- | --- |
| #1 | 전체: (인지행동치료 OR 수면위생 OR 수면교육 OR 수면제한 OR 자극조절 OR 이완 OR 운동 OR 걷기 OR 태극권 OR 침 OR 혈위 OR 지압 OR 이압) | 25999 |
| #2 | 전체: (불면 OR 수면) | 2687 |
| #3 | 전체: (노인 OR 노년) | 11075 |
| #4 | #1 AND #2 AND #3 | **39** |

**Table S12. KCI**

|  | Searches | Results |
| --- | --- | --- |
| #1 | 제목: (인지행동치료 OR 수면위생 OR 수면교육) AND (불면 OR 수면) AND (노인 OR 노년) | 3 |
| #2 | 제목: (수면제한 OR 자극조절 OR 이완) AND (불면 OR 수면) AND (노인 OR 노년) | 0 |
| #3 | 제목: (운동 OR 걷기 OR 태극권) AND (불면 OR 수면) AND (노인 OR 노년) | 8 |
| #4 | 제목: (침 OR 혈위 OR 지압) AND (불면 OR 수면) AND (노인 OR 노년) | 0 |
| #5 | 제목: 이압 AND (불면 OR 수면) AND (노인 OR 노년) | 8 |
| #6 | #1 OR #2 OR #3 OR #4 OR #5 | **19** |

**Table S13. CNKI**

|  | Searches | Results |
| --- | --- | --- |
| #1 | (SU='失眠'+'不寐'+'不眠'+'不睡'+'不得眠'+'不得卧') and (SU='老年'+'老人'+'老龄') and (SU='认知行为治疗'+'认知行为疗法'+'睡眠卫生'+'睡眠教育'+'睡眠限制'+'刺激控制'+'松弛'+'运动'+'步行'+'太极'+'针'+'穴位'+'指压'+'按压'+'耳压') | **90** |

**Table S14. Wanfang data**

|  | Searches | Results |
| --- | --- | --- |
| #1 | (“失眠”+”不寐”+”不眠”+”不睡”+”不得眠”+”不得卧”) AND (“老年”+”老人”+”老龄”) AND (“认知行为治疗”+”认知行为疗法”+”睡眠卫生”+”睡眠教育”+”睡眠限制”+”刺激控制”+”松弛”+”运动”+”步行”+”太极”+”针”+”穴位”+”指压”+”按压”+”耳压”) | **956** |

**Supplementary Material 3. Risk of bias of included studies**

**Figure S1. Risk of bias graph**


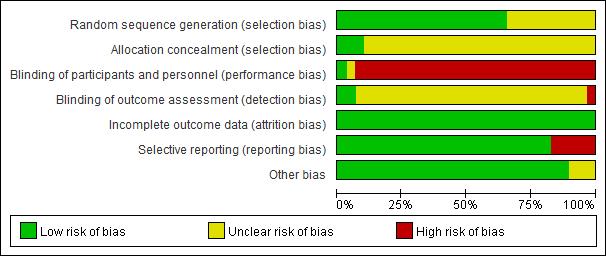


**Figure S2. Risk of bias summary**


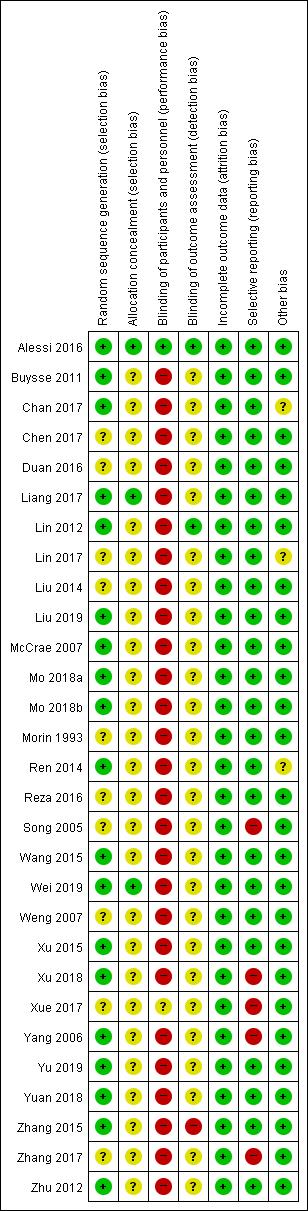


**Supplementary Material 4. Excluded studies and reason after full-text review**

**Table S15. Not RCT (including quasi RCT) (n = 61)**

| **Year** | **Title** | **Journal** |
| --- | --- | --- |
| 1991 | Progressive relaxation and the sleep of older noninstitutionalized women | Appl Nurs Res |
| 1993 | Relaxation for insomnia and hypnotic medication use in older women | Psychology and Aging |
| 1999 | Cognitive behavior therapy, temazepam, or both improved short-term outcomes for older adults with persistent insomnia | Evidence-based medicine |
| 2000 | 노인의 불면증에 대한 이압요법의 효과 | 성인간호학회지 |
| 2000 | 耳穴压迫治疗老年人失眠效果观察 | 福州总医院学报 |
| 2001 | 头、体针结合治疗老年性失眠83例临床研究 | 针灸临床杂志 |
| 2002 | Scalp and body acupuncture for treatment of senile insomnia - a report of 83 cases | Journal of Traditional Chinese Medicine |
| 2003 | The long-term effects of auricular therapy using magnetic pearls on elderly with insomnia | Complementary therapies in medicine |
| 2003 | 针刺结合耳压治疗失眠症110例 | 上海针灸杂志 |
| 2004 | Cognitive behavioral therapy aided discontinuation of benzodiazepine in chronic insomnia | ACP Journal Club |
| 2004 | CBT added to tapering helped patients with chronic insomnia discontinue benzodiazepine use | Evidence Based Medicine |
| 2004 | More promise for the effects of tai chi: improvement in sleep quality for older adults | Focus on Alternative & Complementary Therapies |
| 2004 | 神阙穴外敷治疗老年人失眠 | 四川中医 |
| 2005 | *针灸治疗老年顽固性失眠48例* | 睡眠医学与精神卫生研讨会中国科协2005年学术年会第50分会论文汇编 |
| 2006 | Treatment of chronic insomnia with cognitive behavioral therapy vs zopiclone | JAMA |
| 2006 | 针刺阴经为主治疗老年人失眠症38例 | 河南中医 |
| 2006 | 穴位按压治疗失眠症患者的作用 | 上海护理 |
| 2007 | Auriculotherapy with Magnetic Pellets Produces Longitudinal Changes in Sleep Patterns of Elderly Patients with Insomnia | Journal of Alternative & Complementary Medicine-New York |
| 2007 | 针灸治疗失眠症26例观察 | 中国老年保健医学 |
| 2007 | *护理干预对老年失眠患者的效果观察* | 全国第十届老年护理学术交流暨专题讲座会议 |
| 2008 | 30例理疗配合针灸治疗老年性失眠的临床观察 | 中国老年保健医学 |
| 2009 | 简易太极拳对社区老年人失眠症的疗效观察 | 社区卫生保健 |
| 2009 | 노인의 불면증에 대한 사상체질별 이압요법의 적용 효과 비교연구 - 태음인, 소양인, 소음인 중심으로 | 지역사회간호학회지 |
| 2009 | 针刺加耳穴贴豆法治疗失眠症30例 | 中国社区医师（医学专业半月刊） |
| 2009 | 수면장애 노인에 대한 귀자극요법의 적용효과 | 동서간호학연구지 |
| 2010 | 针刺配合药物治疗失眠32例 | 中外健康文摘 |
| 2010 | 简易太极拳对社区老年人失眠症的疗效观察 | 上海医药 |
| 2010 | 针灸治疗老年性失眠80例观察 | 浙江中医杂志 |
| 2010 | 针刺太溪三阴交涌泉穴治疗老年人失眠40例 | 陕西中医 |
| 2010 | 针刺结合生物反馈疗法治疗老年慢性失眠的临床研究 | 中华行为医学与脑科学杂志 |
| 2011 | Brief behavioral therapy diminishes insomnia in the elderly | Journal of the National Medical Association |
| 2012 | 针刺调理跷脉治疗老年失眠症36例 | 中国保健营养（中旬刊） |
| 2012 | 景衣安神散穴位贴敷治疗失眠临床观察 | 吉林中医药 |
| 2012 | 针灸治疗老年性失眠的临床观察 | 中国中医药现代远程教育 |
| 2013 | The effect of light therapy on the sleep quality of the elderly: an intervention study | Australian Journal of Advanced Nursing |
| 2013 | *针灸配合穴位按摩辨证施护治疗老年性失眠的疗效观察* | 中国针灸学会2013年专题学术会议暨夹脊穴的理论研究与临床应用学术研讨会论文集 |
| 2013 | 调补心肾针刺法治疗老年性失眠症临床观察 | 新中医 |
| 2013 | 中药穴位敷贴治疗老年失眠症50例 | 临床合理用药杂志 |
| 2013 | 温泉水联合运动疗法治疗老年失眠61例临床观察 | 中国疗养医学 |
| 2014 | 交通心肾针法治疗顽固性失眠60例 | 中医药临床杂志 |
| 2014 | 老年睡眠障碍患者护理干预效果观察 | 天津护理 |
| 2015 | 辨证施护在针灸配合穴位按摩治疗老年失眠中的应用效果 | 当代护士(中旬刊) |
| 2015 | 循证护理在老年失眠症患者中的应用 | 医学美学美容（中旬刊） |
| 2015 | 三联疗法在改善老年护理院病人失眠中的疗效观察 | 全科护理 |
| 2016 | 电项针联合臭氧对心脾两虚型老年高血压伴失眠症患者疗效观察 | 现代中西医结合杂志 |
| 2016 | 电项针联合臭氧治疗心脾两虚型老年高血压伴失眠症疗效观察 | 现代中西医结合杂志 |
| 2016 | 针灸治疗老年性失眠临床取穴特点 | 中医药临床杂志 |
| 2016 | **引火归原法对不同证候的老年失眠患者临床疗效差异研究** | 广州中医药大学 |
| 2016 | 腹针十字坐标经典穴组治疗老年失眠症90例临床观察 | 云南中医中药杂志 |
| 2016 | 腹针十字坐标经典穴组治疗老年失眠症临床观察 | 四川中医 |
| 2017 | Acupressure can help improve sleep quality in residents | Canadian Nursing Home |
| 2017 | Effects of sleep management with self-help treatment for the Japanese elderly with chronic insomnia: a quasi-experimental study | Journal of behavioral medicine |
| 2017 | 开设中医失眠门诊在社区老年失眠患者管理中的应用效果 | 中华全科医学 |
| 2017 | 穴位按摩联合理疗在社区老年失眠患者治疗中的应用 | 中国基层医药 |
| 2017 | 老年性失眠患者在服用药物同时使用穴位埋线治疗效果观察 | 华夏医学 |
| 2017 | 佐匹克隆和放松训练结合耳穴贴压治疗老年人失眠30例 | 江西中医药 |
| 2018 | 穴位按摩结合理疗治疗社区老年失眠患者疗效分析 | 现代医学与健康研究电子杂志 |
| 2018 | 心理护理对老年失眠患者睡眠质量的改善疗效观察 | 实用临床护理学电子杂志 |
| 2018 | 睡眠行为干预对老年失眠症患者睡眠质量的影响 | 医疗装备 |
| 2018 | **电针肾俞、会阳穴配合常规针刺治疗老年慢性失眠症的临床观察** | 黑龙江中医药大学 |
| 2019 | 穴位按摩联合情志护理在老年住院失眠患者中的应用效果 | 河南医学研究 |

Note. Italic article title and bolding article title mean conference proceeding and dissertation, respectively.

**Table S16. No information about participants’ age (n = 4)**

| **Year** | **Title** | **Journal** |
| --- | --- | --- |
| 2012 | *CBT treatment of sleep medication dependence* | Sleep |
| 2013 | Scraping technique of stuck needle at Anmian point in the treatment of insomnia: a randomized controlled trial | Zhongguo zhen jiu [Chinese acupuncture & moxibustion] |
| 2014 | A Study to Determine the effectiveness of Therapeutic Back Massage on Quality of Sleep among Elderly in Selected old Age Homes at Mangalore | International Journal of Nursing Education |
| 2018 | 中医杵针疗法结合情志护理在老年失眠伴焦虑症状患者中的应用 | 国际护理学杂志 |

**Table S17. Not appropriate for the participants’ age range (n = 162)**

| **Year** | **Title** | **Journal** |
| --- | --- | --- |
| 1979 | Behavioral treatment of presleep tension and intrusive cognitions in patients with severe predormital insomnia | Journal of behavioral medicine |
| 1979 | Controlled comparison of progressive relaxation, stimulus control, and paradoxical intention therapies for insomnia | Journal of Consulting and Clinical Psychology |
| 1982 | Progressive relaxation, EMG biofeedback and biofeedback placebo in the treatment of sleep-onset insomnia | British journal of medical psychology |
| 1988 | Behavioral and cognitive treatments of geriatric insomnia | J Consult Clin Psychol |
| 1989 | A controlled comparative investigation of psychological treatments for chronic sleep-onset insomnia | Behaviour research and therapy |
| 1989 | Hypnotic relaxation and the reduction of sleep onset insomnia | International journal of psychosomatics |
| 1997 | The efficacy of sleep hygiene measures in the treatment of insomnia | Gaceta medica de mexico |
| 1999 | Cognitive behavior therapy, temazepam, or both improved short-term outcomes for older adults with persistent insomnia...commentary on Morin CM, Colecchi C, Stone J et al. Behavioral and pharmacological therapies for late-life insomnia: a randomized controlled trial. JAMA 1999 Mar 17;281:991-9 | ACP Journal Club |
| 1999 | Acupuncture and insomnia | Forschende komplementarmedizin |
| 1999 | Behavioral and pharmacological therapies for late-life insomnia: a randomized controlled trial | JAMA |
| 2000 | Pharmacologic or behavioural therapy for elderly people's insomnia. Which is better? | Canadian family physician medecin de famille canadien |
| 2001 | Relaxation and sleep compression for late-life insomnia: a placebo-controlled trial | Journal of consulting and clinical psychology |
| 2001 | 耳压磁珠治疗失眠32例 | 中医外治杂志 |
| 2001 | 电针加耳压治疗老年性失眠189例 | 时珍国医国药 |
| 2002 | Short-term training increases diagnostic and treatment rate for insomnia in general practice | European archives of psychiatry and clinical neuroscience |
| 2002 | Efficacy of two behavioral treatment programs for comorbid geriatric insomnia | Psychology and aging |
| 2002 | Clinical observation on intractable insomnia treated by point pressure in 42 cases | Journal of traditional chinese medicine = chung i tsa chih ying wen pan |
| 2003 | Discontinuation of benzodiazepines among older insomniac adults treated with cognitive-behavioural therapy combined with gradual tapering: a randomized trial | CMAJ: Canadian Medical Association Journal |
| 2003 | 针药结合治疗失眠的临床观察 | 针灸临床杂志 |
| 2004 | Comparing Tai Chi Training to a Low-Stress Physical Activity to Enhance Sleep in Older Adults (clinical trial protocol) | Https://clinicaltrials.gov/show/nct00079664 |
| 2004 | Sequential treatment for chronic insomnia: a pilot study | Behavioral sleep medicine |
| 2004 | 子午流注纳甲法治疗失眠症疗效观察 | 黑龙江中医药 |
| 2004 | Randomized clinical trial of supervised tapering and cognitive behavior therapy to facilitate benzodiazepine discontinuation in older adults with chronic insomnia | Am J Psychiatry |
| 2005 | Cognitive-behavioral group therapy as an early intervention for insomnia: a randomized controlled trial | Journal of occupational rehabilitation |
| 2005 | The Effectiveness of Regular Exercise on Improving Sleep in Older Adults (clinical trial protocol) | Https://clinicaltrials.gov/show/nct00149747 |
| 2005 | Sequential combinations of drug and cognitive behavioral therapy for chronic insomnia: an exploratory study | Behaviour research and therapy |
| 2005 | *神阙穴敷贴治疗老年性失眠50例临床观察* | 中国针炙学会2005年学术年会论文汇编 |
| 2005 | 银杏叶制剂神阙穴敷贴治疗老年性失眠25例临床观察 | 中西医结合学报 |
| 2005 | *针刺配合灸关元治疗老年人失眠41例的疗效观察* | 中国针炙学会临床分会第三届全国代表大会暨全国针炙临床学术论坛论文集 |
| 2006 | Cognitive behavioral therapy vs zopiclone for treatment of chronic primary insomnia in older adults: a randomized controlled trial | JAMA |
| 2006 | Effect of rolling needle therapy on quality of life in the patient of non-organic chronic insomnia: a randomized controlled trial | Zhongguo zhen jiu [Chinese acupuncture & moxibustion] |
| 2006 | Multi-central controlled study on three-part massage therapy for treatment of insomnia of deficiency of both the heart and spleen | Zhongguo zhen jiu [Chinese acupuncture & moxibustion] |
| 2006 | 针刺配合灸关元治疗老年人失眠41例的疗效观察 | 云南中医学院学报 |
| 2007 | Clinical observation on effect of electric acupuncture at Sishencong in treating insomnia | Zhongguo zhong xi yi jie he za zhi zhongguo zhongxiyi jiehe zazhi = chinese journal of integrated traditional and western medicine |
| 2007 | Randomized and controlled study on effect of acupuncture on sleep quality in the patient of primary insomnia | Zhongguo zhen jiu [Chinese acupuncture & moxibustion] |
| 2008 | Randomized controlled observation on head point-through-point therapy for treatment of insomnia | Zhongguo zhen jiu [Chinese acupuncture & moxibustion] |
| 2008 | Pretherapy cognitive dispositions and treatment outcome in cognitive behavior therapy for insomnia | Behavior therapy |
| 2008 | Clinical study on the visceral differentiation-based acupuncture therapy for insomnia | Journal of traditional chinese medicine = chung i tsa chih ying wen pan |
| 2008 | Insomnia due to deficiency of both the heart and spleen treated by acupuncture-moxibustion and Chinese tuina | Journal of traditional chinese medicine = chung i tsa chih ying wen pan |
| 2008 | Efficacy of wrists overnight compression (HT 7 point) on insomniacs: possible role of melatonin? | Minerva medica |
| 2008 | Observation on the therapeutic effect of neck clustered needling on insomnia | Zhongguo zhen jiu [Chinese acupuncture & moxibustion] |
| 2008 | Psychological treatment of insomnia in hypnotic-dependant older adults | Sleep medicine |
| 2008 | Comparison of therapeutic effects of electroacupuncture treatment of insomnia at different time | Zhen CI yan jiu = acupuncture research |
| 2008 | Trazodone improves the results of cognitive behaviour therapy of primary insomnia in non-depressed patients | Neuro endocrinology letters |
| 2008 | Effect of acupuncture combined with massage of sole on sleeping quality of the patient with insomnia | Zhongguo zhen jiu [Chinese acupuncture & moxibustion] |
| 2008 | *认知行为疗法联合抗抑郁药治疗慢性失眠症临床效果分析* | 第十次全国行为医学学术会议论文集 |
| 2009 | Clinical observation on therapeutic effect of the pressing plantar reflex area with wooden needle for treatment of patients with insomnia | Zhongguo zhen jiu [Chinese acupuncture & moxibustion] |
| 2009 | Observation on therapeutic effect of Chuzhen therapy on insomnia | Zhongguo zhen jiu [Chinese acupuncture & moxibustion] |
| 2009 | The needle-rolling therapy for treatment of non-organic chronic insomnia in 90 cases | Journal of traditional chinese medicine = chung i tsa chih ying wen pan |
| 2009 | Acupuncture and reflexology for insomnia: a feasibility study | Acupuncture in medicine |
| 2009 | Forty cases of insomnia treated by suspended moxibustion at Baihui (GV 20) | Journal of traditional chinese medicine = chung i tsa chih ying wen pan |
| 2009 | The Complementary Effect of Ear Acupressure on Older Patients With Chronic Insomnia (clinical trial protocol) | Https://clinicaltrials.gov/show/nct00832468 |
| 2009 | Self-help treatment for insomnia through television and book: a randomized trial | Patient education and counseling |
| 2009 | **艾灸及针刺治疗失眠症的临床研究** | 广州中医药大学 |
| 2009 | 24式简易太极拳锻炼对社区老年人失眠症的临床疗效观察 | 中国民间疗法 |
| 2009 | 认知行为疗法联合抗抑郁药治疗慢性失眠症效果分析 | 广东医学 |
| 2010 | An exploratory study on the effects of tele-neurofeedback and tele-biofeedback on objective and subjective sleep in patients with primary insomnia | Applied psychophysiology and biofeedback |
| 2010 | The effect of acupuncture compared with sham acupuncture and estazolam in primary insomnia (clinical trial protocol) | Http://www.who.int/trialsearch/trial2.aspx?Trialid=isrctn12585433 |
| 2010 | Auricular acupuncture for insomnia: a randomized controlled trial | Zhonghua liu xing bing xue za zhi |
| 2010 | Clinical observation on acupuncture treatment of intractable insomnia | Journal of traditional chinese medicine = chung i tsa chih ying wen pan |
| 2010 | Sleep Restriction Is Associated With Increased Morning Plasma Leptin Concentrations, Especially in Women | Biological Research for Nursing |
| 2010 | Sleep restriction therapy and hypnotic withdrawal versus sleep hygiene education in hypnotic using patients with insomnia | Journal of clinical sleep medicine |
| 2010 | Effect of "tranquilization needling" on the sleep quality in patients with insomnia of heart-spleen deficiency type | Zhen CI yan jiu = acupuncture research |
| 2010 | Effect on tranquilizing and allaying excitement needling method on brain blood flow in the patients of insomnia of heart and spleen deficiency | Zhongguo zhen jiu [Chinese acupuncture & moxibustion] |
| 2010 | Scalp penetration acupuncture for insomnia: a randomized controlled trial | Zhong xi yi jie he xue bao [Journal of Chinese integrative medicine] |
| 2010 | Effect of scalp point penetration needling on sleep quality and sleep structure of insomnia patients | Zhongguo zhen jiu [Chinese acupuncture & moxibustion] |
| 2010 | **刺血疗法结合针刺治疗单纯性失眠的临床观察** | 广州中医药大学 |
| 2010 | *阴阳气血配穴针刺法结合耳穴贴压治疗失眠症的临床研究* | 2010国际针法灸法技术演示暨学术研讨会论文集 |
| 2011 | A self-help book is better than sleep hygiene advice for insomnia: a randomized controlled comparative study | Scandinavian journal of psychology |
| 2011 | *脑电仿生电刺激仪治疗失眠的疗效观察* | 中国康复医学会运动疗法分会第十一届全国康复学术大会学术会议论文摘要汇编 |
| 2011 | **针刺配合王不留行籽耳压治疗原发性失眠症的临床研究** | 广州中医药大学 |
| 2011 | **穴位埋线治疗心肾不交型失眠的临床研究** | 广州中医药大学 |
| 2011 | 阴阳气血配穴针刺法结合耳穴贴压治疗失眠症的临床研究 | 辽宁中医杂志 |
| 2012 | Mindfulness-based cognitive therapy improves polysomnographic and subjective sleep profiles in antidepressant users with sleep complaints | Psychotherapy and psychosomatics |
| 2012 | *Dismantling multicomponent behavioral treatment for insomnia in older adults: a randomized controlled trial* | Sleep |
| 2012 | Don't worry, be constructive: a randomized controlled feasibility study comparing behaviour therapy singly and combined with constructive worry for insomnia | The british journal of clinical psychology |
| 2012 | Effectiveness of a cognitive behavioral self-help program for patients with primary insomnia in general practice - a pilot study | Sleep medicine |
| 2012 | Internet-delivered or mailed self-help treatment for insomnia?: a randomized waiting-list controlled trial | Behaviour research and therapy |
| 2012 | The effectiveness of community day-long CBT-I workshops for participants with insomnia symptoms: a randomised controlled trial | Journal of sleep research |
| 2012 | A comparison between acupuncture versus zolpidem in the treatment of primary insomnia | Asian journal of psychiatry |
| 2012 | Clinical observation on insomnia treated with multivariate acupuncture of chronomedicine | Zhongguo zhen jiu [Chinese acupuncture & moxibustion] |
| 2012 | **镇静安神法治疗失眠症（心脾两虚型）临床研究** | 广州中医药大学 |
| 2012 | 华佗夹脊蟠龙针刺法治疗老年顽固性失眠30例 | 亚太传统医药 |
| 2012 | **通督调神法电针配合耳压治疗失眠症临床研究** | 广州中医药大学 |
| 2012 | 穴位按摩治疗老年高血压失眠29例 | 浙江中医杂志 |
| 2013 | Manual-guided cognitive-behavioural therapy for insomnia delivered by ordinary primary care personnel in general medical practice: a randomized controlled effectiveness trial | Journal of sleep research |
| 2013 | Sleep disorder treated mainly with flying needling therapy: a clinical randomized controlled research | Zhongguo zhen jiu [Chinese acupuncture & moxibustion] |
| 2013 | The Stimulation Effect of Auricular Magnetic Press Pellets on Older Female Adults with Sleep Disturbance Undergoing Polysomnographie Evaluation | Evidence-based Complementary & Alternative Medicine (eCAM) |
| 2013 | Curative effect of acupuncture and moxibustion on insomnia: a randomized clinical trial | Journal of traditional chinese medicine = chung i tsa chih ying wen pan |
| 2013 | Efficacy of acupuncture for primary insomnia: a randomized controlled clinical trial | Evidence-based complementary and alternative medicine |
| 2013 | Efficacy of repetitive transcranial magnetic stimulation in the treatment of patients with chronic primary insomnia | Cell biochemistry and biophysics |
| 2013 | Motivational support provided via email improves the effectiveness of internet-delivered self-help treatment for insomnia: a randomized trial | Behaviour research and therapy |
| 2013 | Efficacy observation on refractory insomnia treated with the balance needling therapy | Zhongguo zhen jiu [Chinese acupuncture & moxibustion] |
| 2013 | Efficacy observation on acupuncture prescription of regulating yin-yang and five viscera for intractable insomnia | Zhongguo zhen jiu [Chinese acupuncture & moxibustion] |
| 2014 | Mindfulness Training Versus Sleep Hygiene for Insomnia Symptoms in Older Adults: A Randomized Controlled Comparison Trial | Journal of Alternative & Complementary Medicine |
| 2014 | Auricular point compression in the treatment of 54 patients with type 2 diabetes insomnia | International Journal of Clinical Acupuncture |
| 2014 | Yoga for improving sleep quality and quality of life for older adults | Altern Ther Health Med |
| 2014 | Quality of life improvements after acceptance and commitment therapy in nonresponders to cognitive behavioral therapy for primary insomnia | Psychotherapy & Psychosomatics |
| 2014 | *Cognitive Behavioral Therapy vs. Tai Chi for Late Life Insomnia and Inflammatory Risk: A Randomized Controlled Comparative Efficacy Trial* | Sleep |
| 2014 | Guided Internet-delivered cognitive behavioural treatment for insomnia: a randomized trial | Psychological medicine |
| 2014 | Low Resistance Thought Induction Sleep-regulating Technique (TIP3-2) combined with medication for primary insomnia: a randomized controlled trial | International journal of behavioral medicine |
| 2014 | Effect of pricking Shēnmài (BL 62) and Zhàohăi (KI 6) on daytime arousal of patients with chronic insomnia: a randomized controlled trial | World journal of acupuncture - moxibustion |
| 2014 | Clinical observation on the neurotransmitters regulation in patients of insomnia differentiated as yang deficiency pattern treated with warm acupuncture and auricular point sticking therapy | Zhongguo zhen jiu [Chinese acupuncture & moxibustion] |
| 2014 | Curative observation on Needling Neiguan (PC6), Zusanli (ST36), and Sanyinjiao (SP6) to treat insomnia | International Journal of Clinical Acupuncture |
| 2014 | Effect of acupressure on sleep quality of middle-aged and elderly patients with hypertension | International journal of nursing sciences |
| 2014 | 腕踝针治疗失眠症48例 | 中国老年学杂志 |
| 2015 | Internet-vs. group-delivered cognitive behavior therapy for insomnia: A randomized controlled non-inferiority trial | Behav Res Ther |
| 2015 | Improved sleep quality in older adults with insomnia reduces biomarkers of disease risk: pilot results from a randomized controlled comparative efficacy trial | Psychoneuroendocrinology |
| 2015 | Increased physical activity improves sleep and mood outcomes in inactive people with insomnia: a randomized controlled trial | Journal of sleep research |
| 2015 | Cognitive behavioral therapy and tai chi reverse cellular and genomic markers of inflammation in late-life insomnia: a randomized controlled trial | Biol Psychiatry |
| 2015 | Expressions of neurotransmitters in patients of insomnia differentiated as liver stagnation transforming into fire treated with acupuncture | Zhongguo zhen jiu [Chinese acupuncture & moxibustion] |
| 2015 | Sleep-Related Safety Behaviors and Dysfunctional Beliefs Mediate the Efficacy of Online CBT for Insomnia: a Randomized Controlled Trial | Cognitive behaviour therapy |
| 2015 | Cognitive Arousal, Unhelpful Beliefs and Maladaptive Sleep Behaviors as Mediators in Cognitive Behavior Therapy for Insomnia: a Quasi-Experimental Study | Cognitive therapy and research |
| 2015 | Treatment of insomnia with shujing massage therapy: a randomized controlled trial | Zhongguo zhen jiu [Chinese acupuncture & moxibustion] |
| 2015 | Effectiveness of a CBT Intervention for Persistent Insomnia and Hypnotic Dependency in an Outpatient Psychiatry Clinic | Journal of clinical psychology |
| 2015 | Mindfulness meditation and improvement in sleep quality and daytime impairment among older adults with sleep disturbances: a randomized clinical trial | JAMA Intern Med |
| 2015 | 中药穴位贴敷联合耳穴压豆治疗高血压失眠94例 | 中国中医药科技 |
| 2015 | 放松训练配合耳穴压籽对失眠患者睡眠质量的疗效 | 中医临床研究 |
| 2015 | 太极拳联合穴位按揉治疗老年慢性失眠患者的疗效观察 | 中华物理医学与康复杂志 |
| 2015 | *太极拳联合穴位按揉治疗老年慢性失眠患者的疗效观察* | 第四届吞咽障碍高峰论坛论文集 |
| 2015 | 针对性护理干预对内科老年失眠患者的影响 | 临床合理用药杂 |
| 2016 | Auricular Acupuncture and Cognitive Behavioural Therapy for Insomnia: a Randomised Controlled Study | Sleep disorders |
| 2016 | *Three-Year Follow-Up of Insomnia and Hypnotics after Controlled Internet Treatment for Insomnia* | Sleep |
| 2016 | Group cognitive behavioural treatment for insomnia in primary care: a randomized controlled trial | Psychological medicine |
| 2016 | Acupuncture at SP6 Point in Lower Extremities Improved Patients' Insomnia, Which Was Also Shown Through Ryodoraku Electrical Measurements | Acupuncture & electro-therapeutics research |
| 2016 | Effects of Aquatic Exercise on Sleep in Older Adults with Mild Sleep Impairment: a Randomized Controlled Trial | International journal of behavioral medicine |
| 2016 | *Guided Online or Face-to-Face Cognitive Behavioral Treatment for Insomnia: a Randomized Wait-List Controlled Trial* | Sleep |
| 2016 | Efficacy of dexmedetomidine with cognitive behavioral therapy for treating chronic insomnia related to conditioned arousal: a randomized controlled trial | Sleep and biological rhythms |
| 2016 | Cognitive-behavior therapy singly and combined with medication for persistent insomnia: Impact on psychological and daytime functioning | Behav Res Ther |
| 2016 | Implementation of Brief Insomnia Treatments - Clinical Trial (clinical trial protocol) | Https://clinicaltrials.gov/show/nct02724800 |
| 2016 | The Effects of Exercise Training in Community-dwelling Elderly With Sleep Disturbances With Follow-up (clinical trial protocol) | Https://clinicaltrials.gov/show/nct03005990 |
| 2016 | Brief behavioral treatment for patients with treatment-resistant insomnia | Neuropsychiatric disease and treatment |
| 2016 | Enhancing CBT for Chronic Insomnia: a Randomised Clinical Trial of Additive Components of Mindfulness or Cognitive Therapy | Clinical psychology & psychotherapy |
| 2016 | 微创穴位埋线治疗痰热内扰型不寐临床观察 | 世界中医药 |
| 2016 | 中医内科护理干预老年患者失眠80例探讨 | 饮食保健 |
| 2016 | 循经点穴疗法对老年围绝经期妇女睡眠障碍患者睡眠质量与心理状态的影响 | 辽宁中医杂志 |
| 2017 | Computerized Cognitive Behavioral Therapy for Insomnia in a Community Health Setting | Journal of clinical sleep medicine |
| 2017 | Comparative effectiveness of three occupational therapy sleep interventions: A randomized controlled study | OTJR Occup Particip Health |
| 2017 | Mobile Phone-Delivered Cognitive Behavioral Therapy for Insomnia: a Randomized Waitlist Controlled Trial | Journal of medical internet research |
| 2017 | Comparison Between Acupuncture and Biofeedback as Adjunctive Treatments for Primary Insomnia Disorder | Alternative therapies in health and medicine |
| 2017 | Cognitive behavioral therapy using a mobile application synchronizable with wearable devices for insomnia treatment: a pilot study | Journal of clinical sleep medicine |
| 2017 | Attentional bias modification training for insomnia: a double-blind placebo controlled randomized trial | Plos one |
| 2017 | Effect of a Web-Based Cognitive Behavior Therapy for Insomnia Intervention With 1-Year Follow-up: a Randomized Clinical Trial | JAMA psychiatry |
| 2017 | Efficacy and safety of acupuncture treatment on primary insomnia: a randomized controlled trial | Sleep Medicine |
| 2017 | Penetration needling on complementary acupoints for insomnia | World Journal of Acupuncture - Moxibustion |
| 2017 | **张铁忠教授防治老年病学术思想及从“六郁”论治忧郁伤神型不寐临床研究** | 中国中医科学院 |
| 2017 | 针灸治疗失眠疗效分析 | 实用中医药杂志 |
| 2017 | **隔药灸脐法对肾虚衰老患者睡眠质量的临床研究** | 山东中医药大学 |
| 2017 | 体感音乐疗法联合穴位按摩治疗失眠的临床观察与护理研究 | 医学信息 |
| 2018 | Effectiveness of cognitive behavioral therapy for pharmacotherapy-resistant chronic insomnia: a multi-center randomized controlled trial in Japan | Sleep medicine |
| 2018 | Non-pharmacological interventions for sleep and quality of life: a randomized pilot study | Revista Latino-Americana de Enfermagem (RLAE) |
| 2018 | A randomized controlled pilot study of CBT-I Coach: feasibility, acceptability, and potential impact of a mobile phone application for patients in cognitive behavioral therapy for insomnia | Health informatics journal |
| 2018 | Hypnotherapy for insomnia: a randomized controlled trial comparing generic and disease-specific suggestions | Complementary therapies in medicine |
| 2018 | Effectiveness of sleep self‐management group intervention in Chinese patients with insomnia disorder | Perspectives in Psychiatric Care |
| 2018 | Comparative effectiveness of mindfulness-based therapy in sleep quality of chronic insomnia compared to standard cognitive behavioral therapy : a randomized controlled trial | Chotmaihet thangphaet [journal of the medical association of thailand] |
| 2018 | Effect of Acupuncture Cooperated with Low-frequency Repetitive Transcranial Magnetic Stimulation on Chronic Insomnia: a Randomized Clinical Trial | Current medical science |
| 2018 | 针刺四神聪配合甜梦口服液治疗老年失眠症的疗效观察 | 世界睡眠医学杂志 |
| 2018 | 针药结合治疗失眠的临床疗效 | 中国老年学杂志 |
| 2018 | 揿针耳穴联合经穴治疗心脾两虚型老年失眠症临床疗效观察 | 辽宁中医药大学学报 |
| 2019 | Randomized Controlled Trial to Test the Efficacy of an Unguided Online Intervention with Automated Feedback for the Treatment of Insomnia | Behavioural and cognitive psychotherapy |
| 2019 | Comparing the Effects of Single‐ and Multiple‐Component Therapies for Insomnia on Sleep Outcomes | Worldviews on Evidence-Based Nursing |
| 2019 | 针对性护理对提高老年冠心病并失眠患者睡眠质量的效果观察 | 健康必读 |

Note. Italic article title and bolding article title mean conference proceeding and dissertation, respectively.

**Table S18. Only describing the mean age of participants (n = 29)**

| **Year** | **Title** | **Journal** |
| --- | --- | --- |
| 2001 | 行为疗法与药物治疗老年失眠症的效果分析 | 现代康复 |
| 2002 | Are changes in beliefs and attitudes about sleep related to sleep improvements in the treatment of insomnia? | Behaviour research and therapy |
| 2002 | 行为疗法与药物治疗老年失眠症的对照研究 | 中国行为医学科学 |
| 2006 | 老年糖尿病患者失眠的综合护理干预 | 护理学杂志 |
| 2009 | *Efficacy of Brief Behavioral Treatment for Insomnia (BBTI) in older adults with insomnia: Final results* | Sleep |
| 2009 | 老年人睡眠障碍综合康复治疗的疗效分析 | 中华物理医学与康复杂志 |
| 2010 | Effectiveness of acupressure for residents of long-term care facilities with insomnia: a randomized controlled trial | Int J Nurs Stud |
| 2010 | 低频脉冲电刺激对老年失眠症患者睡眠结构的影响 | 中国康复 |
| 2011 | *A randomized controlled trial of a combined treatment of cognitive behaviour therapy and evening bright light therapy for insomnia in older adults* | Sleep |
| 2011 | *老年高血压患者睡眠问题的健康行为干预* | 浙江省中西医结合学会成立三十周年纪念会 |
| 2012 | *A randomized controlled trial of a combined treatment of cognitive behaviour therapy and evening bright light therapy for insomnia in older adults* | Sleep and biological rhythms |
| 2012 | 老年高血压患者睡眠的健康行为干预 | 浙江预防医学 |
| 2013 | *Evaluation of a brief treatment program of cognitive-behavior therapy for insomnia in older adults* | Sleep |
| 2014 | *Cognitive-behavioral therapy for insomnia in older adults: Improving sleep quality reduces sleep discrepancy* | Sleep |
| 2014 | *Untreated mild sleep disordered breathing does not reduce the efficacy of cognitive behavioral therapy for insomnia in older adults* | Sleep |
| 2014 | *Evaluation of a brief treatment program of cognitive behavior therapy for insomnia in older adults* | Sleep |
| 2014 | 护理干预对老年住院患者睡眠质量的影响 | 中外健康文摘 |
| 2014 | *Cognitive behavioural therapy for treatment of insomnia in older adults with symptomatic knee osteoarthritis: a randomized trial* | Annals of the rheumatic diseases |
| 2015 | *The therapeutic effect of cognitive-behaviour therapy for older adults suffering from insomnia with short objective sleep duration: a randomized controlled trial* | Sleep and biological rhythms |
| 2015 | *The therapeutic effect of cognitive-behaviour therapy for insomniacs with short objective sleep duration: a randomized controlled trial* | Sleep |
| 2015 | 穴位敷贴联合耳穴压豆对老年性失眠的护理疗效观察 | 湖南中医药大学学报 |
| 2016 | *A randomised controlled trial of auricular acupuncture and cognitive behavioural therapy for insomnia: a short-term self-assessment* | Journal of sleep research |
| 2016 | *Auricular acupuncture and cognitive behavioural therapy for insomnia-a randomised controlled study* | Journal of sleep research |
| 2016 | *Comparing the therapeutic effect of cognitive-behavior therapy for older adults suffering from insomnia with short and normal objective sleep duration* | Journal of sleep research |
| 2016 | Tai Chi in combination with acupoint massage can improve sleep quality of elderly patients with chronic insomnia | International journal of clinical and experimental medicine |
| 2016 | 健步走联合穴位按揉及音乐放松训练治疗老年慢性失眠患者的疗效观察 | 中华物理医学与康复杂志 |
| 2017 | Auricular acupuncture versus cognitive behavioural therapy in the discontinuation of hypnotic drug usage, and treatment effects on anxiety, depression and insomnia symptoms − a randomised controlled study | European Journal of Integrative Medicine |
| 2017 | 舒眠胶囊配合针灸四神聪治疗老年失眠症的疗效观察 | 中国医药指南 |
| 2018 | 耳穴压豆治疗老年失眠患者的临床护理干预效果分析 | 中国保健营养 |

Note. Italic article title means conference proceeding.

**Table S19. Cluster RCT (n = 8)**

| **Year** | **Title** | **Journal** |
| --- | --- | --- |
| 2009 | Cognitive behavioral therapy for insomnia improves sleep and decreases pain in older adults with co-morbid insomnia and osteoarthritis | Journal of clinical sleep medicine |
| 2011 | *Cognitive behavior therapy for insomnia improves sleep and decreases pain in older adults with co-morbid insomnia and osteoarthritis* | Sleep and Biological Rhythms |
| 2012 | *Cognitive behavioral therapy for sleep and pain in older adults with co-morbid insomnia and osteoarthritis: results of the lifestyles randomized controlled trial* | Sleep |
| 2012 | Group interventions for co-morbid insomnia and osteoarthritis pain in primary care: the lifestyles cluster randomized trial design | Contemporary clinical trials |
| 2013 | Cognitive-behavioral treatment for comorbid insomnia and osteoarthritis pain in primary care: the lifestyles randomized controlled trial | J Am Geriatr Soc |
| 2014 | Short-term improvement in insomnia symptoms predicts long-term improvements in sleep, pain, and fatigue in older adults with comorbid osteoarthritis and insomnia | Pain |
| 2014 | *Improved sleep quality predicts long-term improvements in sleep, pain, and fatigue in older adults with co-morbid osteoarthritis and insomnia* | Sleep |
| 2019 | The Impact of Benson's Relaxation Technique on the Quality of Sleep in the Elderly | Topics in geriatric rehabilitation |

Note. Italic article title means conference proceeding.

**Table S20. Inappropriate for the population criteria (n = 8)**

| **Year** | **Title** | **Journal** |
| --- | --- | --- |
| 1997 | *A randomized controlled trial of the effect of exercise on sleep* | Sleep |
| 2003 | Older adults and withdrawal from benzodiazepine hypnotics in general practice: effects on cognitive function, sleep, mood and quality of life | Psychological medicine |
| 2003 | Behavioral treatment of insomnia in older adults: an open clinical trial comparing two interventions | Behav Res Ther |
| 2006 | Effects of a brief behavioral treatment for late-life insomnia: preliminary findings | Journal of clinical sleep medicine |
| 2009 | Natural light exposure improves subjective sleep quality in nursing home residents | Journal of the american medical directors association |
| 2014 | 住院老年患者失眠的原因分析及护理干预 | 医学信息 |
| 2018 | A Secondary Analysis of Sleep Quality Changes in Older Adults From a Randomized Trial of an MBSR Program | Journal of applied gerontology |
| 2019 | 老年失眠患者睡眠影响因素与护理对策 | 养生保健指南 |

Note. Italic article title means conference proceeding.

**Table S21. Using not defined diagnostic criteria (n = 24)**

| **Year** | **Title** | **Journal** |
| --- | --- | --- |
| 1993 | The effectiveness of acupressure in improving the quality of sleep of institutionalized residents | Journals of gerontology. Series A, Biological sciences and medical sciences |
| 1994 | **A behavioral intervention to enhance the sleep-wake patterns of older adults with insomnia** | UNIVERSITY OF ARIZONA |
| 2002 | Effectiveness of auricular therapy on sleep promotion in the elderly | American Journal of Chinese Medicine |
| 2005 | 耳穴疗法治疗老人失眠症之临床研究 | 针灸临床杂志 |
| 2010 | The effect of acupressure on quality of sleep in Iranian elderly nursing home residents | Complement Ther Clin Pract |
| 2011 | **社区老年失眠患者自我管理模式研究** | 河南大学 |
| 2011 | 社区老年患者失眠自我管理模式研究 | 中华护理杂志 |
| 2011 | 社区老年失眠患者的自我管理模式探讨 | 世界感染杂志 |
| 2012 | 睡眠卫生指导配合音乐疗法治疗老年失眠症的临床观察 | 国际护理学杂志 |
| 2012 | 药物和认知行为疗法治疗社区老年人慢性失眠的随机对照研究 | 中国临床医学 |
| 2013 | Effect of Resistance Training Over Aerobic Exercise in Improving Quality of Sleep in Older Adults | Indian Journal of Physiotherapy & Occupational Therapy |
| 2013 | Self-relaxation training can improve sleep quality and cognitive functions in the older: a one-year randomised controlled trial | Journal of clinical nursing |
| 2013 | 认知行为干预对老年高血压患者失眠的影响 | 中国现代医学杂志 |
| 2013 | **노인 불면에 대한 EFT 불면치료 프로그램(EFT-I)의 효과** | 경희대학교 |
| 2015 | 循证护理在老年高血压失眠患者中的应用 | 福建医药杂志 |
| 2016 | Comparing and contrasting therapeutic effects of cognitive-behavior therapy for older adults suffering from insomnia with short and long objective sleep duration | Sleep medicine |
| 2016 | The Effects of Acupressure Training on Sleep Quality and Cognitive Function of Older Adults: a 1-Year Randomized Controlled Trial | Research in nursing & health |
| 2017 | Acupressure, Sleep, and Quality of Life in Institutionalized Older Adults: a Randomized Controlled Trial | Journal of the american geriatrics society |
| 2017 | 经络刺激改善老年失眠患者 睡眠质量的疗效观察 | 中医药通报 |
| 2018 | **八段锦锻炼对社区老年人群睡眠状况的短期影响** | 河北医科大学 |
| 2018 | 复方精油穴位按摩对老年人睡眠质量及认知功能的影响 | 中国老年学杂志 |
| 2018 | 探索中医内科护理干预老年失眠患者160例分析 | 健康必读 |
| 2018 | 中药穴位贴敷联合耳穴压豆治疗老年高血压失眠临床观察 | 实用中医药杂志 |
| 2019 | Effects of Acupressure on Sleep Quality and Psychological Distress in Nursing Home Residents: a Randomized Controlled Trial | Journal of the american medical directors association |

Note. Bolding article title means dissertation.

**Table S22. Not described the diagnostic criteria (n = 12)**

| **Year** | **Title** | **Journal** |
| --- | --- | --- |
| 2008 | 头电针治疗241例老年性失眠的临床疗效观察 | 亚太传统医药 |
| 2010 | Effects of a Bedtime Massage on Relaxation in Nursing Home Residents With Sleep Disorders | Activities, Adaptation and Aging |
| 2012 | Cognitive behavioral therapy for institutionalized elders complaining of sleep disturbance in Alexandria, Egypt | Sleep and breathing |
| 2012 | 对健康教育改善老年人失眠症状作用的研究 | 中国保健营养（下旬刊） |
| 2014 | 针对性护理干预对住院老年患者失眠的影响 | 齐鲁护理杂志 |
| 2015 | 中医护理干预对老年失眠患者的疗效观察 | 中外女性健康研究 |
| 2015 | “健康小屋”互助课堂健康管理模式应用于老年失眠患者的研究 | 基层医学论坛 |
| 2016 | 针刺四神聪联合耳穴埋籽治疗老年性失眠的临床疗效观察 | 中国医药导刊 |
| 2016 | 试析老年患者失眠的中医内科护理干预措施 | 中国保健营养 |
| 2017 | 老年失眠症杵针疗法的效果 | 中国老年学杂志 |
| 2018 | Effects of Brief Cognitive Behavioral Therapy for insomnia on sleep and usage of hypnotics among community-dwelling older adults: randomized controlled trial | Nihon koshu eisei zasshi [Japanese journal of public health] |
| 2019 | 针对性优质护理在老年慢性病失眠患者中的应用效果观察 | 世界睡眠医学杂志 |

**Table S23. Inappropriate for the intervention criteria (n = 25)**

| **Year** | **Title** | **Journal** |
| --- | --- | --- |
| 2009 | 针药并用治疗老年气血亏虚型失眠144例临床观察 | 河北中医 |
| 2009 | **通督调神法针刺治疗老年失眠临床研究** | 广州中医药大学 |
| 2010 | 调督补肾法治疗老年失眠症疗效观察 | 上海针灸杂志 |
| 2011 | 穴位埋线结合药物治疗老年性失眠的疗效 | 中国老年学杂志 |
| 2011 | 加味吴茱萸散穴位贴敷治疗老年失眠临床观察 | 辽宁中医杂志 |
| 2013 | Cognitive training improves sleep quality and cognitive function among older adults with insomnia | Plos one |
| 2013 | *Cognitive training improves sleep quality and cognitive function among older adults with insomnia* | Sleep medicine |
| 2013 | A randomized control trial for the evaluation of the effects of EFT-insomnia (EFT-I) for the elderly | Psychotherapy and Psychosomatics |
| 2013 | **杵针对老年失眠的临床操作规范化研究** | 成都中医药大学 |
| 2013 | 基于体质的饮食调护在老年失眠患者中的应用研究 | 中国临床护理 |
| 2014 | *Cognitive training improves sleep quality and cognitive function among older adults with insomnia* | Journal of molecular neuroscience. |
| 2014 | 按摩穴位配合音乐疗法改善老年失眠的临床效果分析 | 中外医疗 |
| 2015 | Comparing the effects of reflexology and footbath on sleep quality in the elderly: a controlled clinical trial | Iranian red crescent medical journal |
| 2015 | **“四神针”结合刮痧疗法治疗老年性失眠的临床研究** | 广州中医药大学 |
| 2016 | *Cognitive training improves sleep quality and cognitive function among older adults with insomnia* | Journal of sleep research |
| 2017 | *Auriculotherapy for insomnia in elderly people: a 6 week, double-blinded, randomised pilot study* | Lancet |
| 2017 | **不同穴位杵针治疗老年心脾两虚型失眠的临床疗效观察** | 成都中医药大学 |
| 2018 | 针药结合治疗老年性失眠的临床观察 | 中国保健营养 |
| 2018 | **耳穴压豆结合穴位按摩对老年高血压伴失眠患者的护理效果观察** | 黑龙江中医药大学 |
| 2018 | 彝药穴位敷贴联合耳穴压豆对老年性失眠患者的护理效果探讨 | 养生保健指南 |
| 2018 | **单穴与配穴治疗老年性失眠的临床对照研究** | 长春中医药大学 |
| 2018 | **隔药灸脐法治疗心肾不交型老年心绞痛患者失眠的临床研究** | 山东中医药大学 |
| 2018 | 天然负氧离子吸入疗法对老年非急性失眠症的影响 | 中国疗养医学 |
| 2019 | Comparison of Magnetic Auriculotherapy, Laser Auriculotherapy and Their Combination for Treatment of Insomnia in the Elderly: A Double-Blinded Randomised Trial | Evidence-based Complementary & Alternative Medicine (eCAM) |
| 2019 | 探讨中医内科护理干预治疗老年失眠临床效果 | 家庭医药 |

Note. Italic article title and bolding article title mean conference proceeding and dissertation, respectively.

**Table S24. Unclearly described intervention (n = 3)**

| **Year** | **Title** | **Journal** |
| --- | --- | --- |
| 2014 | *运动训练对老年失眠症的疗效观察及生活质量影响研究* | 2014浙江省医学会精神病学分会学术年会、浙江省医师协会精神科医师分会第七届年会 |
| 2015 | 穴位按摩结合理疗治疗老年失眠患者疗效分析 | 浙江临床医学 |
| 2016 | 高龄老年失眠症患者的康复治疗 | 浙江临床医学 |

Note. Italic article title means conference proceeding.

**Table S25. Using same data from other papers (n = 4)**

| **Year** | **Title** | **Journal** |
| --- | --- | --- |
| 2010 | *针灸结合生物反馈疗法治疗慢性失眠症的临床研究* | 第十二次全国行为医学学术会议论文集 |
| 2011 | *规律有氧运动训练对老年失眠症的疗效观察* | 2011年浙江省医学会精神病学分会老年精神障碍学组学术会议 |
| 2015 | 针药结合治疗老年性失眠:随机对照研究 | 中国针灸 |
| 2017 | *Impact of brief behavioral treatment for insomnia (BBT-I) on sleep and cognition in older adults with insomnia: the rest randomized controlled trial* | Sleep |

Note. Italic article title means conference proceeding.

**Table S26. Secondary analyses not relevant to the interests of this review (n = 8)**

| **Year** | **Title** | **Journal** |
| --- | --- | --- |
| 2014 | *A randomized controlled trial of behavioral treatment for insomnia in older veterans* | Journal of the american geriatrics society |
| 2014 | *Cognitive behavioral therapy for insomnia in older veterans: final results of a randomized trial* | Sleep |
| 2014 | Behavioral treatment of insomnia: also effective for nocturia | Journal of the american geriatrics society |
| 2014 | *Behavioral treatment of chronic insomnia in older adults: does nocturia matter?* | Sleep |
| 2018 | Efficacy of brief behavioral treatment for insomnia in older adults: examination of sleep, mood, and cognitive outcomes | Sleep medicine |
| 2018 | Sleep outcomes with cognitive behavioral therapy for insomnia are similar between older adults with low vs. high self-reported physical activity | Frontiers in Aging Neuroscience |
| 2019 | CBT for late-life insomnia and the accuracy of sleep and wake perceptions: results from a randomized-controlled trial | Journal of sleep research |
| 2019 | *Impact of brief behavioral treatment for insomnia (BBTI) on meta-cognition in older adults* | Sleep |

Note. Italic article title means conference proceeding.

**Supplementary Material 5. The network map, results of inconsistency test, network forest plot, interval plot, cumulative probability plot, SUCRA, mean rank, and netleague table in NMA for the outcomes**

**1. PSQI total score**

**Figure S3. Network map**

**Abbreviations:** ACU, acupuncture; BT, behavioral treatment; BZD, benzodiazepines; CBT, cognitive behavioral therapy; EDU, sleep education; EXR, exercise; MTN, melatonin; QIG, qigong; RLX, relaxation.

**Figure S4. Inconsistency test**

**Figure S5. Network forest plot**

**Abbreviations:** ACU, acupuncture; BT, behavioral treatment; BZD, benzodiazepines; CBT, cognitive behavioral therapy; EDU, sleep education; EXR, exercise; MTN, melatonin; QIG, qigong; RLX, relaxation.

**Figure S6. Interval plot**

**Abbreviations:** ACU, acupuncture; BT, behavioral treatment; BZD, benzodiazepines; CBT, cognitive behavioral therapy; EDU, sleep education; EXR, exercise; MTN, melatonin; QIG, qigong; RLX, relaxation.

**Figure S7. Cumulative probability plot**

**Abbreviations:** ACU, acupuncture; BT, behavioral treatment; BZD, benzodiazepines; CBT, cognitive behavioral therapy; EDU, sleep education; EXR, exercise; MTN, melatonin; QIG, qigong; RLX, relaxation.

**Figure S8. SUCRA**

**Abbreviations:** ACU, acupuncture; BT, behavioral treatment; BZD, benzodiazepines; CBT, cognitive behavioral therapy; EDU, sleep education; EXR, exercise; MTN, melatonin; QIG, qigong; RLX, relaxation.

**Figure S9. Mean rank**

**Abbreviations:** ACU, acupuncture; BT, behavioral treatment; BZD, benzodiazepines; CBT, cognitive behavioral therapy; EDU, sleep education; EXR, exercise; MTN, melatonin; QIG, qigong; RLX, relaxation.

**Figure S10. Mean rank (after removing outlier)**


**Abbreviations:** ACU, acupuncture; BT, behavioral treatment; BZD, benzodiazepines; CBT, cognitive behavioral therapy; EDU, sleep education; EXR, exercise; MTN, melatonin; QIG, qigong; RLX, relaxation.

**Table S27. Netleague table (SMD, 95% CI)**

| **WLT** | **-4.37 (-8.53,-0.21)** | **-5.20 (-9.82,-0.57)** | **-4.18 (-9.08,0.72)** | **-10.44 (-17.31,-3.58)** | **-4.28 (-8.45,-0.11)** | **-7.18 (-12.17,-2.19)** | **-5.41 (-11.21,0.38)** | **-4.93 (-8.63,-1.22)** | -4.31 (-9.78,1.15) | **-4.38 (-9.42,0.67)** | **-5.46 (-10.94,0.02)** | -2.44 (-5.98,1.09) |
| --- | --- | --- | --- | --- | --- | --- | --- | --- | --- | --- | --- | --- |
| **4.37 (0.21,8.53)** | **ACU** | -0.82 (-3.10,1.45) | 0.19 (-3.72,4.11) | **-6.07 (-12.91,0.77)** | 0.09 (-1.23,1.42) | -2.81 (-6.91,1.30) | -1.04 (-5.27,3.19) | -0.55 (-4.22,3.11) | 0.06 (-5.38,5.50) | -0.01 (-2.86,2.85) | -1.09 (-6.54,4.37) | 1.93 (-1.60,5.46) |
| **5.20 (0.57,9.82)** | 0.82 (-1.45,3.10) | **ACU+BZD** | 1.02 (-3.48,5.51) | -5.25 (-12.34,1.85) | 0.92 (-1.25,3.09) | -1.99 (-6.47,2.50) | -0.22 (-4.79,4.35) | 0.27 (-3.84,4.38) | 0.88 (-4.87,6.63) | 0.82 (-2.83,4.47) | -0.26 (-6.03,5.50) | 2.75 (-1.38,6.89) |
| **4.18 (-0.72,9.08)** | -0.19 (-4.11,3.72) | -1.02 (-5.51,3.48) | **ACU+RLX** | -6.26 (-13.87,1.34) | -0.10 (-4.18,3.98) | -3.00 (-8.47,2.46) | -1.24 (-6.96,4.49) | -0.75 (-5.70,4.20) | -0.14 (-6.51,6.24) | -0.20 (-5.04,4.64) | -1.28 (-7.67,5.11) | 1.73 (-2.18,5.65) |
| **10.44 (3.58,17.31)** | **6.07 (-0.77,12.91)** | 5.25 (-1.85,12.34) | 6.26 (-1.34,13.87) | **BT** | **6.16 (-0.61,12.94)** | 3.26 (-3.74,10.26) | 5.03 (-2.85,12.91) | **5.52 (-0.27,11.30)** | **6.13 (1.98,10.28)** | 6.06 (-1.35,13.48) | 4.98 (-2.07,12.04) | **8.00 (0.87,15.13)** |
| **4.28 (0.11,8.45)** | -0.09 (-1.42,1.23) | -0.92 (-3.09,1.25) | 0.10 (-3.98,4.18) | **-6.16 (-12.94,0.61)** | **BZD** | -2.90 (-6.84,1.04) | -1.14 (-5.15,2.88) | -0.65 (-4.19,2.89) | -0.04 (-5.39,5.32) | -0.10 (-3.25,3.05) | -1.18 (-6.55,4.19) | 1.83 (-1.82,5.49) |
| **7.18 (2.19,12.17)** | 2.81 (-1.30,6.91) | 1.99 (-2.50,6.47) | 3.00 (-2.46,8.47) | -3.26 (-10.26,3.74) | 2.90 (-1.04,6.84) | **BZD+CBT** | 1.77 (-3.86,7.39) | 2.25 (-1.70,6.20) | 2.87 (-2.77,8.50) | 2.80 (-2.20,7.80) | 1.72 (-3.93,7.37) | **4.74 (-0.23,9.70)** |
| **5.41 (-0.38,11.21)** | 1.04 (-3.19,5.27) | 0.22 (-4.35,4.79) | 1.24 (-4.49,6.96) | -5.03 (-12.91,2.85) | 1.14 (-2.88,5.15) | -1.77 (-7.39,3.86) | **BZD+EXR** | 0.49 (-4.87,5.84) | 1.10 (-5.60,7.80) | 1.04 (-4.07,6.14) | -0.04 (-6.75,6.66) | 2.97 (-2.46,8.40) |
| **4.93 (1.22,8.63)** | 0.55 (-3.11,4.22) | -0.27 (-4.38,3.84) | 0.75 (-4.20,5.70) | **-5.52 (-11.30,0.27)** | 0.65 (-2.89,4.19) | -2.25 (-6.20,1.70) | -0.49 (-5.84,4.87) | **CBT** | 0.61 (-3.41,4.64) | 0.55 (-4.09,5.19) | -0.53 (-4.57,3.51) | 2.48 (-1.69,6.65) |
| 4.31 (-1.15,9.78) | -0.06 (-5.50,5.38) | -0.88 (-6.63,4.87) | 0.14 (-6.24,6.51) | **-6.13 (-10.28,-1.98)** | 0.04 (-5.32,5.39) | -2.87 (-8.50,2.77) | -1.10 (-7.80,5.60) | -0.61 (-4.64,3.41) | **EDU** | -0.06 (-6.21,6.08) | -1.14 (-6.85,4.56) | 1.87 (-3.92,7.66) |
| **4.38 (-0.67,9.42)** | 0.01 (-2.85,2.86) | -0.82 (-4.47,2.83) | 0.20 (-4.64,5.04) | -6.06 (-13.48,1.35) | 0.10 (-3.05,3.25) | -2.80 (-7.80,2.20) | -1.04 (-6.14,4.07) | -0.55 (-5.19,4.09) | 0.06 (-6.08,6.21) | **MTN** | -1.08 (-7.24,5.08) | 1.93 (-2.60,6.47) |
| **5.46 (-0.02,10.94)** | 1.09 (-4.37,6.54) | 0.26 (-5.50,6.03) | 1.28 (-5.11,7.67) | -4.98 (-12.04,2.07) | 1.18 (-4.19,6.55) | -1.72 (-7.37,3.93) | 0.04 (-6.66,6.75) | 0.53 (-3.51,4.57) | 1.14 (-4.56,6.85) | 1.08 (-5.08,7.24) | **QIG** | 3.01 (-2.79,8.82) |
| 2.44 (-1.09,5.98) | -1.93 (-5.46,1.60) | -2.75 (-6.89,1.38) | -1.73 (-5.65,2.18) | **-8.00 (-15.13,-0.87)** | -1.83 (-5.49,1.82) | **-4.74 (-9.70,0.23)** | -2.97 (-8.40,2.46) | -2.48 (-6.65,1.69) | -1.87 (-7.66,3.92) | -1.93 (-6.47,2.60) | -3.01 (-8.82,2.79) | **RLX** |

**Abbreviations**: ACU, acupuncture; BT, behavioral treatment; BZD, benzodiazepines; CBT, cognitive behavioral therapy; EDU, sleep education; EXR, exercise; MTN, melatonin; QIG, qigong; RLX, relaxation.

**Table S28. Netleague table (after removing outlier) (SMD, 95% CI)**

| **WLT** | **-4.27 (-8.57,0.04)** | **-5.93 (-10.99,-0.87)** | **-4.10 (-9.16,0.97)** | **-10.52 (-17.61,-3.43)** | **-4.41 (-8.72,-0.10)** | **-7.28 (-12.43,-2.13)** | **-5.54 (-11.53,0.44)** | **-5.00 (-8.82,-1.18)** | -4.39 (-10.04,1.26) | **-4.27 (-9.49,0.95)** | **-5.53 (-11.19,0.13)** | -2.39 (-6.04,1.26) |
| --- | --- | --- | --- | --- | --- | --- | --- | --- | --- | --- | --- | --- |
| **4.27 (-0.04,8.57)** | **ACU** | -1.66 (-4.59,1.26) | 0.17 (-3.87,4.21) | **-6.25 (-13.33,0.82)** | -0.14 (-1.59,1.31) | -3.02 (-7.28,1.24) | -1.28 (-5.68,3.12) | -0.74 (-4.54,3.06) | -0.12 (-5.76,5.51) | -0.01 (-2.96,2.94) | -1.27 (-6.92,4.38) | 1.88 (-1.77,5.52) |
| **5.93 (0.87,10.99)** | 1.66 (-1.26,4.59) | **ACU+BZD** | 1.83 (-3.12,6.79) | -4.59 (-12.10,2.92) | 1.52 (-1.25,4.29) | -1.35 (-6.26,3.56) | 0.38 (-4.61,5.38) | 0.93 (-3.63,5.48) | 1.54 (-4.63,7.71) | 1.66 (-2.50,5.81) | 0.40 (-5.79,6.58) | 3.54 (-1.06,8.14) |
| **4.10 (-0.97,9.16)** | -0.17 (-4.21,3.87) | -1.83 (-6.79,3.12) | **ACU+RLX** | -6.42 (-14.29,1.44) | -0.31 (-4.55,3.92) | -3.19 (-8.84,2.47) | -1.45 (-7.38,4.49) | -0.91 (-6.03,4.22) | -0.29 (-6.89,6.30) | -0.17 (-5.18,4.83) | -1.44 (-8.04,5.17) | 1.71 (-2.34,5.75) |
| **10.52 (3.43,17.61)** | **6.25 (-0.82,13.33)** | 4.59 (-2.92,12.10) | 6.42 (-1.44,14.29) | **BT** | **6.11 (-0.89,13.11)** | 3.23 (-4.00,10.47) | 4.97 (-3.17,13.11) | **5.52 (-0.45,11.48)** | **6.13 (1.85,10.41)** | 6.25 (-1.42,13.91) | 4.98 (-2.30,12.27) | **8.13 (0.76,15.49)** |
| **4.41 (0.10,8.72)** | 0.14 (-1.31,1.59) | -1.52 (-4.29,1.25) | 0.31 (-3.92,4.55) | **-6.11 (-13.11,0.89)** | **BZD** | -2.88 (-6.95,1.20) | -1.14 (-5.29,3.02) | -0.59 (-4.25,3.06) | 0.02 (-5.52,5.56) | 0.14 (-3.15,3.42) | -1.13 (-6.68,4.43) | 2.02 (-1.78,5.81) |
| **7.28 (2.13,12.43)** | 3.02 (-1.24,7.28) | 1.35 (-3.56,6.26) | 3.19 (-2.47,8.84) | -3.23 (-10.47,4.00) | 2.88 (-1.20,6.95) | **BZD+CBT** | 1.74 (-4.08,7.56) | 2.28 (-1.80,6.36) | 2.89 (-2.93,8.72) | 3.01 (-2.17,8.20) | 1.75 (-4.09,7.59) | **4.89 (-0.25,10.03)** |
| **5.54 (-0.44,11.53)** | 1.28 (-3.12,5.68) | -0.38 (-5.38,4.61) | 1.45 (-4.49,7.38) | -4.97 (-13.11,3.17) | 1.14 (-3.02,5.29) | -1.74 (-7.56,4.08) | **BZD+EXR** | 0.54 (-4.99,6.08) | 1.15 (-5.77,8.08) | 1.27 (-4.02,6.57) | 0.01 (-6.92,6.94) | 3.15 (-2.47,8.78) |
| **5.00 (1.18,8.82)** | 0.74 (-3.06,4.54) | -0.93 (-5.48,3.63) | 0.91 (-4.22,6.03) | **-5.52 (-11.48,0.45)** | 0.59 (-3.06,4.25) | -2.28 (-6.36,1.80) | -0.54 (-6.08,4.99) | **CBT** | 0.61 (-3.55,4.77) | 0.73 (-4.08,5.54) | -0.53 (-4.71,3.65) | 2.61 (-1.70,6.93) |
| 4.39 (-1.26,10.04) | 0.12 (-5.51,5.76) | -1.54 (-7.71,4.63) | 0.29 (-6.30,6.89) | **-6.13 (-10.41,-1.85)** | -0.02 (-5.56,5.52) | -2.89 (-8.72,2.93) | -1.15 (-8.08,5.77) | -0.61 (-4.77,3.55) | **EDU** | 0.12 (-6.24,6.48) | -1.14 (-7.04,4.75) | 2.00 (-3.99,7.99) |
| **4.27 (-0.95,9.49)** | 0.01 (-2.94,2.96) | -1.66 (-5.81,2.50) | 0.17 (-4.83,5.18) | -6.25 (-13.91,1.42) | -0.14 (-3.42,3.15) | -3.01 (-8.20,2.17) | -1.27 (-6.57,4.02) | -0.73 (-5.54,4.08) | -0.12 (-6.48,6.24) | **MTN** | -1.26 (-7.63,5.11) | 1.88 (-2.81,6.57) |
| **5.53 (-0.13,11.19)** | 1.27 (-4.38,6.92) | -0.40 (-6.58,5.79) | 1.44 (-5.17,8.04) | -4.98 (-12.27,2.30) | 1.13 (-4.43,6.68) | -1.75 (-7.59,4.09) | -0.01 (-6.94,6.92) | 0.53 (-3.65,4.71) | 1.14 (-4.75,7.04) | 1.26 (-5.11,7.63) | **QIG** | 3.14 (-2.86,9.15) |
| 2.39 (-1.26,6.04) | -1.88 (-5.52,1.77) | -3.54 (-8.14,1.06) | -1.71 (-5.75,2.34) | **-8.13 (-15.49,-0.76)** | -2.02 (-5.81,1.78) | **-4.89 (-10.03,0.25)** | -3.15 (-8.78,2.47) | -2.61 (-6.93,1.70) | -2.00 (-7.99,3.99) | -1.88 (-6.57,2.81) | -3.14 (-9.15,2.86) | **RLX** |

**Abbreviations**: ACU, acupuncture; BT, behavioral treatment; BZD, benzodiazepines; CBT, cognitive behavioral therapy; EDU, sleep education; EXR, exercise; MTN, melatonin; QIG, qigong; RLX, relaxation.

**2. Polysomnography data**

**Figure S11. Network map**

**Abbreviations:** ACU, acupuncture; BT, behavioral treatment; BZD, benzodiazepines; CBT, cognitive behavioral therapy; EDU, sleep education; WLT, wait-list.

**3. Drop-outs for any reasons**

**Figure S12. Network map**

**Abbreviations**: ACU, acupuncture; ATC, attention control; BT, behavioral treatment; BZD, benzodiazepines; CBT, cognitive behavioral therapy; EDU, sleep education; EXR, exercise; MTN, melatonin; QIG, qigong; RLX, relaxation; SSRI, selective serotonin reuptake inhibitor; WLT, wait-list.

**Figure S13. Inconsistency test**

**Figure S14. Network forest plot**

**Abbreviations**: ACU, acupuncture; ATC, attention control; BT, behavioral treatment; BZD, benzodiazepines; CBT, cognitive behavioral therapy; EDU, sleep education; EXR, exercise; MTN, melatonin; QIG, qigong; RLX, relaxation; SSRI, selective serotonin reuptake inhibitor; WLT, wait-list.

**Figure S15. Interval plot**

**Abbreviations**: ACU, acupuncture; ATC, attention control; BT, behavioral treatment; BZD, benzodiazepines; CBT, cognitive behavioral therapy; EDU, sleep education; EXR, exercise; MTN, melatonin; QIG, qigong; RLX, relaxation; SSRI, selective serotonin reuptake inhibitor; WLT, wait-list.

**Figure S16. Cumulative probability plot**

**Abbreviations**: ACU, acupuncture; ATC, attention control; BT, behavioral treatment; BZD, benzodiazepines; CBT, cognitive behavioral therapy; EDU, sleep education; EXR, exercise; MTN, melatonin; QIG, qigong; RLX, relaxation; SSRI, selective serotonin reuptake inhibitor; WLT, wait-list.

**Figure S17. SUCRA**

**Abbreviations**: ACU, acupuncture; ATC, attention control; BT, behavioral treatment; BZD, benzodiazepines; CBT, cognitive behavioral therapy; EDU, sleep education; EXR, exercise; MTN, melatonin; QIG, qigong; RLX, relaxation; SSRI, selective serotonin reuptake inhibitor; WLT, wait-list.

**Figure S18. Mean rank**

**Abbreviations**: ACU, acupuncture; ATC, attention control; BT, behavioral treatment; BZD, benzodiazepines; CBT, cognitive behavioral therapy; EDU, sleep education; EXR, exercise; MTN, melatonin; QIG, qigong; RLX, relaxation; SSRI, selective serotonin reuptake inhibitor; WLT, wait-list.

**Table S29. Netleague table (OR, 95% CI)**

| **WLT** | 0.94 (0.09,9.48) | 0.93 (0.04,20.41) | 1.41 (0.02,99.89) | 2.72 (0.01,902.73) | 0.43 (0.03,5.43) | 33.08 (0.16,7010.22) | 20.13 (0.11,3656.54) | 0.97 (0.09,10.38) | 1.06 (0.06,20.29) | 0.95 (0.01,93.77) | 1.41 (0.28,7.10) | 2.80 (0.04,197.24) | 0.94 (0.03,35.33) | 0.80 (0.10,6.07) | 0.57 (0.07,4.83) | 0.43 (<0.01,46.71) |
| --- | --- | --- | --- | --- | --- | --- | --- | --- | --- | --- | --- | --- | --- | --- | --- | --- |
| 1.06 (0.11,10.66) | **ACU** | 0.98 (0.12,8.36) | 1.49 (0.01,155.99) | 2.89 (0.01,1279.20) | 0.46 (0.11,1.93) | 35.09 (0.12,10163.88) | 21.35 (0.09,5347.96) | 1.03 (0.42,2.55) | 1.13 (0.11,11.89) | 1.01 (0.02,57.08) | 1.49 (0.13,17.53) | 2.97 (0.03,308.22) | 1.00 (0.06,16.34) | 0.85 (0.05,13.24) | 0.61 (0.17,2.19) | 0.46 (0.01,30.42) |
| 1.08 (0.05,23.85) | 1.02 (0.12,8.68) | **ACU+BZD** | 1.52 (0.01,242.77) | 2.94 (<0.01,1811.15) | 0.47 (0.04,6.13) | 35.76 (0.09,14728.81) | 21.76 (0.06,7817.63) | 1.05 (0.13,8.49) | 1.15 (0.06,23.87) | 1.03 (0.01,88.31) | 1.52 (0.06,37.00) | 3.03 (0.02,479.99) | 1.02 (0.03,34.45) | 0.86 (0.03,26.30) | 0.62 (0.05,7.39) | 0.47 (<0.01,51.72) |
| 0.71 (0.01,50.32) | 0.67 (0.01,69.85) | 0.66 (<0.01,104.67) | **ACU+CBT** | 1.93 (<0.01,1782.94) | 0.31 (<0.01,36.57) | 23.48 (0.04,14869.99) | 14.29 (0.03,7966.30) | 0.69 (0.01,72.60) | 0.75 (0.01,102.65) | 0.68 (<0.01,299.74) | 1.00 (0.02,51.54) | 1.99 (0.01,521.99) | 0.67 (<0.01,151.60) | 0.57 (0.01,35.16) | 0.41 (<0.01,40.81) | 0.31 (<0.01,150.47) |
| 0.37 (<0.01,121.71) | 0.35 (<0.01,153.31) | 0.34 (<0.01,209.01) | 0.52 (<0.01,477.22) | **ACU+EDU** | 0.16 (<0.01,77.84) | 12.15 (0.07,2028.02) | 7.39 (0.05,1050.01) | 0.36 (<0.01,159.04) | 0.39 (<0.01,212.01) | 0.35 (<0.01,496.13) | 0.52 (<0.01,136.27) | 1.03 (0.02,53.41) | 0.35 (<0.01,282.13) | 0.29 (<0.01,88.17) | 0.21 (<0.01,90.39) | 0.16 (<0.01,245.06) |
| 2.31 (0.18,29.02) | 2.18 (0.52,9.18) | 2.14 (0.16,28.07) | 3.26 (0.03,388.24) | 6.30 (0.01,3087.11) | **ACU+RLX** | 76.50 (0.24,24712.64) | 46.55 (0.17,13039.75) | 2.25 (0.42,12.13) | 2.45 (0.16,37.42) | 2.21 (0.03,158.66) | 3.26 (0.22,48.66) | 6.49 (0.05,767.27) | 2.18 (0.09,50.48) | 1.85 (0.09,35.91) | 1.32 (0.28,6.15) | 1.00 (0.02,51.42) |
| 0.03 (<0.01,6.40) | 0.03 (<0.01,8.25) | 0.03 (<0.01,11.52) | 0.04 (<0.01,26.97) | 0.08 (<0.01,13.74) | 0.01 (<0.01,4.22) | **ATC** | 0.61 (0.17,2.18) | 0.03 (<0.01,8.57) | 0.03 (<0.01,11.59) | 0.03 (<0.01,28.70) | 0.04 (<0.01,7.03) | 0.08 (<0.01,2.20) | 0.03 (<0.01,15.83) | 0.02 (<0.01,4.60) | 0.02 (<0.01,4.86) | 0.01 (<0.01,14.24) |
| 0.05 (<0.01,9.02) | 0.05 (<0.01,11.73) | 0.05 (<0.01,16.51) | 0.07 (<0.01,39.03) | 0.14 (<0.01,19.22) | 0.02 (<0.01,6.02) | 1.64 (0.46,5.88) | **BT** | 0.05 (<0.01,12.18) | 0.05 (<0.01,16.56) | 0.05 (<0.01,41.89) | 0.07 (<0.01,9.83) | 0.14 (0.01,2.79) | 0.05 (<0.01,22.84) | 0.04 (<0.01,6.46) | 0.03 (<0.01,6.90) | 0.02 (<0.01,20.81) |
| 1.03 (0.10,10.99) | 0.97 (0.39,2.40) | 0.95 (0.12,7.69) | 1.45 (0.01,152.62) | 2.80 (0.01,1249.05) | 0.44 (0.08,2.40) | 34.04 (0.12,9929.17) | 20.71 (0.08,5225.40) | **BZD** | 1.09 (0.12,10.02) | 0.98 (0.02,49.93) | 1.45 (0.12,17.28) | 2.89 (0.03,301.56) | 0.97 (0.05,18.30) | 0.82 (0.05,13.03) | 0.59 (0.13,2.72) | 0.44 (0.01,32.33) |
| 0.94 (0.05,18.00) | 0.89 (0.08,9.38) | 0.87 (0.04,18.13) | 1.33 (0.01,180.82) | 2.57 (<0.01,1395.30) | 0.41 (0.03,6.21) | 31.16 (0.09,11252.12) | 18.96 (0.06,5953.63) | 0.92 (0.10,8.40) | **BZD+CBT** | 0.90 (0.01,81.80) | 1.33 (0.07,24.96) | 2.64 (0.02,357.42) | 0.89 (0.02,34.35) | 0.75 (0.03,18.07) | 0.54 (0.04,7.18) | 0.41 (<0.01,49.01) |
| 1.05 (0.01,102.95) | 0.99 (0.02,55.70) | 0.97 (0.01,82.99) | 1.48 (<0.01,653.30) | 2.85 (<0.01,4040.03) | 0.45 (0.01,32.58) | 34.66 (0.03,34491.43) | 21.09 (0.02,18635.54) | 1.02 (0.02,51.77) | 1.11 (0.01,101.21) | **BZD+EXR** | 1.48 (0.01,153.63) | 2.94 (0.01,1293.12) | 0.99 (0.01,133.40) | 0.84 (0.01,102.00) | 0.60 (0.01,40.66) | 0.45 (<0.01,151.76) |
| 0.71 (0.14,3.58) | 0.67 (0.06,7.85) | 0.66 (0.03,15.95) | 1.00 (0.02,51.54) | 1.93 (0.01,509.11) | 0.31 (0.02,4.58) | 23.48 (0.14,3874.82) | 14.29 (0.10,2005.45) | 0.69 (0.06,8.22) | 0.75 (0.04,14.17) | 0.68 (0.01,70.49) | **CBT** | 1.99 (0.04,101.71) | 0.67 (0.02,27.72) | 0.57 (0.17,1.93) | 0.41 (0.04,4.43) | 0.31 (<0.01,36.50) |
| 0.36 (0.01,25.07) | 0.34 (<0.01,34.83) | 0.33 (<0.01,52.23) | 0.50 (<0.01,131.73) | 0.97 (0.02,50.36) | 0.15 (<0.01,18.24) | 11.80 (0.45,305.91) | 7.18 (0.36,143.50) | 0.35 (<0.01,36.20) | 0.38 (<0.01,51.21) | 0.34 (<0.01,149.73) | 0.50 (0.01,25.67) | **EDU** | 0.34 (<0.01,75.68) | 0.28 (<0.01,17.52) | 0.20 (<0.01,20.35) | 0.15 (<0.01,75.17) |
| 1.06 (0.03,39.75) | 1.00 (0.06,16.34) | 0.98 (0.03,33.17) | 1.49 (0.01,338.59) | 2.89 (<0.01,2354.13) | 0.46 (0.02,10.62) | 35.09 (0.06,19489.65) | 21.35 (0.04,10413.06) | 1.03 (0.05,19.45) | 1.13 (0.03,43.55) | 1.01 (0.01,136.69) | 1.49 (0.04,61.90) | 2.97 (0.01,669.72) | **MTN** | 0.85 (0.02,42.67) | 0.61 (0.03,13.13) | 0.46 (<0.01,70.82) |
| 1.25 (0.16,9.53) | 1.18 (0.08,18.48) | 1.16 (0.04,35.35) | 1.77 (0.03,109.60) | 3.41 (0.01,1026.76) | 0.54 (0.03,10.55) | 41.45 (0.22,7907.93) | 25.22 (0.15,4111.71) | 1.22 (0.08,19.32) | 1.33 (0.06,31.97) | 1.20 (0.01,145.87) | 1.77 (0.52,6.01) | 3.51 (0.06,216.35) | 1.18 (0.02,59.55) | **QIG** | 0.72 (0.05,10.51) | 0.54 (<0.01,75.22) |
| 1.75 (0.21,14.74) | 1.65 (0.46,5.94) | 1.62 (0.14,19.33) | 2.46 (0.02,247.43) | 4.76 (0.01,2047.50) | 0.76 (0.16,3.51) | 57.81 (0.21,16233.03) | 35.18 (0.14,8534.35) | 1.70 (0.37,7.85) | 1.86 (0.14,24.71) | 1.67 (0.02,113.09) | 2.46 (0.23,26.86) | 4.90 (0.05,488.86) | 1.65 (0.08,35.63) | 1.39 (0.10,20.45) | **RLX** | 0.76 (0.01,51.89) |
| 2.31 (0.02,249.75) | 2.18 (0.03,144.57) | 2.14 (0.02,236.73) | 3.26 (0.01,1597.30) | 6.30 (<0.01,9718.97) | 1.00 (0.02,51.42) | 76.50 (0.07,83336.72) | 46.55 (0.05,45096.91) | 2.25 (0.03,163.24) | 2.45 (0.02,295.34) | 2.21 (0.01,739.13) | 3.26 (0.03,387.50) | 6.49 (0.01,3161.95) | 2.18 (0.01,336.63) | 1.85 (0.01,256.16) | 1.32 (0.02,90.86) | **SSRI** |

**Abbreviations**: ACU, acupuncture; ATC, attention control; BT, behavioral treatment; BZD, benzodiazepines; CBT, cognitive behavioral therapy; EDU, sleep education; EXR, exercise; MTN, melatonin; QIG, qigong; RLX, relaxation; SSRI, selective serotonin reuptake inhibitor; WLT, wait-list.

**4. Drop-outs for AEs**

**Figure S19. Network map**

**Abbreviations**: ACU, acupuncture; AE, adverse event; ATC, attention control; BT, behavioral treatment; BZD, benzodiazepines; CBT, cognitive behavioral therapy; EDU, sleep education; EXR, exercise; MTN, melatonin; QIG, qigong; RLX, relaxation; WLT, wait-list.

**Figure S20. Inconsistency test**

**Figure S21. Network forest plot**

**Abbreviations**: ACU, acupuncture; AE, adverse event; ATC, attention control; BT, behavioral treatment; BZD, benzodiazepines; CBT, cognitive behavioral therapy; EDU, sleep education; EXR, exercise; MTN, melatonin; QIG, qigong; RLX, relaxation; WLT, wait-list.

**Figure S22. Interval plot**

**Abbreviations**: ACU, acupuncture; AE, adverse event; ATC, attention control; BT, behavioral treatment; BZD, benzodiazepines; CBT, cognitive behavioral therapy; EDU, sleep education; EXR, exercise; MTN, melatonin; QIG, qigong; RLX, relaxation; WLT, wait-list.

**Figure S23. Cumulative probability plot**

**Abbreviations**: ACU, acupuncture; AE, adverse event; ATC, attention control; BT, behavioral treatment; BZD, benzodiazepines; CBT, cognitive behavioral therapy; EDU, sleep education; EXR, exercise; MTN, melatonin; QIG, qigong; RLX, relaxation; WLT, wait-list.

**Figure S24. SUCRA**

**Abbreviations**: ACU, acupuncture; AE, adverse event; ATC, attention control; BT, behavioral treatment; BZD, benzodiazepines; CBT, cognitive behavioral therapy; EDU, sleep education; EXR, exercise; MTN, melatonin; QIG, qigong; RLX, relaxation; WLT, wait-list.

**Figure S25. Mean rank**

**Abbreviations**: ACU, acupuncture; AE, adverse event; ATC, attention control; BT, behavioral treatment; BZD, benzodiazepines; CBT, cognitive behavioral therapy; EDU, sleep education; EXR, exercise; MTN, melatonin; QIG, qigong; RLX, relaxation; WLT, wait-list.

**Table S30. Netleague table (OR, 95% CI)**

| **WLT** | 0.98 (<0.01,248.04) | 0.95 (<0.01,309.63) | 1.00 (<0.01,274.38) | 1.93 (<0.01,1840.95) | 2.02 (<0.01,5523.27) | 1.90 (<0.01,1801.31) | 0.99 (<0.01,216.60) | 1.01 (<0.01,220.11) | 0.97 (<0.01,765.55) | 1.00 (0.02,54.46) | 1.99 (0.01,542.85) | 0.98 (<0.01,482.40) | 1.00 (<0.01,275.07) | 1.00 (0.02,51.93) |
| --- | --- | --- | --- | --- | --- | --- | --- | --- | --- | --- | --- | --- | --- | --- |
| 1.02 (<0.01,258.48) | **ACU** | 0.97 (0.11,8.90) | 1.02 (<0.01,248.43) | 1.97 (<0.01,1704.10) | 2.06 (<0.01,5180.59) | 1.94 (<0.01,1667.34) | 1.01 (0.28,3.61) | 1.03 (0.08,13.97) | 0.99 (0.02,61.81) | 1.02 (0.02,46.89) | 2.03 (0.01,491.44) | 1.00 (0.06,16.34) | 1.02 (<0.01,249.07) | 1.02 (<0.01,915.85) |
| 1.05 (<0.01,342.72) | 1.03 (0.11,9.45) | **ACU+BZD** | 1.05 (<0.01,329.97) | 2.03 (<0.01,2161.78) | 2.13 (<0.01,6392.82) | 2.00 (<0.01,2115.34) | 1.04 (0.13,8.64) | 1.06 (0.05,23.71) | 1.02 (0.01,88.78) | 1.05 (0.02,68.99) | 2.09 (0.01,652.93) | 1.03 (0.03,36.46) | 1.05 (<0.01,330.78) | 1.05 (<0.01,1160.51) |
| 1.00 (<0.01,274.38) | 0.98 (<0.01,238.39) | 0.95 (<0.01,298.10) | **ACU+CBT** | 1.93 (<0.01,1783.00) | 2.02 (<0.01,5372.35) | 1.90 (<0.01,1744.59) | 0.99 (<0.01,207.94) | 1.01 (<0.01,211.31) | 0.97 (<0.01,740.77) | 1.00 (0.02,51.54) | 1.99 (0.01,522.00) | 0.98 (<0.01,465.63) | 1.00 (<0.01,264.52) | 1.00 (<0.01,957.92) |
| 0.52 (<0.01,492.72) | 0.51 (<0.01,437.67) | 0.49 (<0.01,522.72) | 0.52 (<0.01,477.21) | **ACU+EDU** | 1.05 (<0.01,974.90) | 0.98 (<0.01,260.29) | 0.51 (<0.01,392.82) | 0.52 (<0.01,399.18) | 0.50 (<0.01,1130.52) | 0.52 (<0.01,136.26) | 1.03 (0.02,53.41) | 0.51 (<0.01,761.99) | 0.52 (<0.01,478.20) | 0.52 (<0.01,1416.58) |
| 0.49 (<0.01,1351.79) | 0.48 (<0.01,1216.70) | 0.47 (<0.01,1413.52) | 0.49 (<0.01,1314.85) | 0.96 (<0.01,891.48) | **ATC** | 0.94 (0.02,48.79) | 0.49 (<0.01,1110.40) | 0.50 (<0.01,1128.39) | 0.48 (<0.01,2796.46) | 0.49 (<0.01,457.23) | 0.98 (<0.01,261.62) | 0.48 (<0.01,1973.33) | 0.49 (<0.01,1317.20) | 0.49 (<0.01,3429.85) |
| 0.53 (<0.01,500.57) | 0.52 (<0.01,444.63) | 0.50 (<0.01,531.07) | 0.53 (<0.01,484.81) | 1.02 (<0.01,270.26) | 1.07 (0.02,55.39) | **BT** | 0.52 (<0.01,399.04) | 0.53 (<0.01,405.51) | 0.51 (<0.01,1148.92) | 0.53 (<0.01,138.34) | 1.05 (0.02,54.15) | 0.52 (<0.01,774.28) | 0.53 (<0.01,485.81) | 0.53 (<0.01,1439.73) |
| 1.01 (<0.01,220.11) | 0.99 (0.28,3.52) | 0.96 (0.12,7.94) | 1.01 (<0.01,211.31) | 1.95 (<0.01,1491.47) | 2.04 (<0.01,4610.48) | 1.91 (<0.01,1459.21) | **BZD** | 1.02 (0.10,9.90) | 0.98 (0.02,49.93) | 1.01 (0.03,37.25) | 2.01 (0.01,417.95) | 0.99 (0.05,21.26) | 1.01 (<0.01,211.87) | 1.01 (<0.01,802.12) |
| 0.99 (<0.01,216.60) | 0.97 (0.07,13.19) | 0.94 (0.04,21.08) | 0.99 (<0.01,207.94) | 1.92 (<0.01,1467.70) | 2.01 (<0.01,4536.99) | 1.88 (<0.01,1435.96) | 0.98 (0.10,9.59) | **BZD+CBT** | 0.97 (0.01,90.62) | 0.99 (0.03,36.66) | 1.97 (0.01,411.29) | 0.97 (0.02,44.40) | 0.99 (<0.01,208.50) | 0.99 (<0.01,789.34) |
| 1.03 (<0.01,806.62) | 1.01 (0.02,62.49) | 0.98 (0.01,84.51) | 1.03 (<0.01,780.52) | 1.98 (<0.01,4450.61) | 2.07 (<0.01,12039.22) | 1.95 (<0.01,4356.23) | 1.02 (0.02,51.77) | 1.03 (0.01,97.03) | **BZD+EXR** | 1.03 (<0.01,213.03) | 2.04 (<0.01,1545.71) | 1.01 (0.01,147.13) | 1.03 (<0.01,782.18) | 1.03 (<0.01,2380.73) |
| 1.00 (0.02,54.46) | 0.98 (0.02,45.00) | 0.95 (0.01,62.33) | 1.00 (0.02,51.54) | 1.93 (0.01,509.12) | 2.02 (<0.01,1868.20) | 1.90 (0.01,497.82) | 0.99 (0.03,36.66) | 1.01 (0.03,37.25) | 0.97 (<0.01,202.18) | **CBT** | 1.99 (0.04,101.71) | 0.98 (0.01,111.92) | 1.00 (0.02,51.73) | 1.00 (<0.01,275.84) |
| 0.50 (<0.01,136.99) | 0.49 (<0.01,119.01) | 0.48 (<0.01,148.86) | 0.50 (<0.01,131.73) | 0.97 (0.02,50.36) | 1.02 (<0.01,269.76) | 0.95 (0.02,49.17) | 0.50 (<0.01,103.79) | 0.51 (<0.01,105.47) | 0.49 (<0.01,370.20) | 0.50 (0.01,25.67) | **EDU** | 0.49 (<0.01,232.60) | 0.50 (<0.01,132.06) | 0.50 (<0.01,478.80) |
| 1.02 (<0.01,502.70) | 1.00 (0.06,16.34) | 0.97 (0.03,34.33) | 1.02 (<0.01,485.22) | 1.97 (<0.01,2966.85) | 2.06 (<0.01,8402.20) | 1.94 (<0.01,2903.51) | 1.01 (0.05,21.81) | 1.03 (0.02,47.02) | 0.99 (0.01,145.51) | 1.02 (0.01,116.63) | 2.03 (<0.01,960.53) | **MTN** | 1.02 (<0.01,486.34) | 1.02 (<0.01,1589.92) |
| 1.00 (<0.01,275.07) | 0.98 (<0.01,239.01) | 0.95 (<0.01,298.84) | 1.00 (<0.01,264.52) | 1.93 (<0.01,1786.69) | 2.02 (<0.01,5381.98) | 1.90 (<0.01,1748.20) | 0.99 (<0.01,208.49) | 1.01 (<0.01,211.87) | 0.97 (<0.01,742.35) | 1.00 (0.02,51.73) | 1.99 (0.01,523.33) | 0.98 (<0.01,466.69) | **QIG** | 1.00 (<0.01,959.89) |
| 1.00 (0.02,51.93) | 0.98 (<0.01,878.86) | 0.95 (<0.01,1048.44) | 1.00 (<0.01,957.92) | 1.93 (<0.01,5292.75) | 2.02 (<0.01,14014.03) | 1.90 (<0.01,5180.86) | 0.99 (<0.01,789.34) | 1.01 (<0.01,802.13) | 0.97 (<0.01,2259.49) | 1.00 (<0.01,275.84) | 1.99 (<0.01,1897.34) | 0.98 (<0.01,1525.70) | 1.00 (<0.01,959.89) | **RLX** |

**Abbreviations**: ACU, acupuncture; AE, adverse event; ATC, attention control; BT, behavioral treatment; BZD, benzodiazepines; CBT, cognitive behavioral therapy; EDU, sleep education; EXR, exercise; MTN, melatonin; QIG, qigong; RLX, relaxation; WLT, wait-list.

**Supplementary Material 6. The results of pair-wise meta-analysis for the outcomes**

**Table S31. PSQI total score**

| **Outcome** |  |  | **Studies** | **Participants** | **SMD [95% CI**] | ***I^2^*** | **Z** | **P** | **Model** |
| --- | --- | --- | --- | --- | --- | --- | --- | --- | --- |
| PSQI | ACU | ACU+BZD | 2 | 120 | -0.33 [-0.70, 0.04] | 88 | 1.76 | 0.08 | fixed |
|  | ACU | ACU+RLX | 1 | 89 | **0.56 [0.14, 0.99]** | NA | 2.61 | 0.009 | fixed |
|  | ACU | BZD | 9 | 535 | -0.15 [-1.08, 0.79] | 96 | 0.30 | 0.76 | random |
|  | ACU | MTN | 2 | 140 | -0.00 [-0.34, 0.33] | 0 | 0.02 | 0.98 | fixed |
|  | ACU | RLX | 1 | 90 | **-0.50 [-0.92, -0.08]** | NA | 2.33 | 0.02 | fixed |
|  | ACU+BZD | BZD | 3 | 195 | **-1.24 [-1.56, -0.91]** | 95 | 7.40 | <0.00001 | fixed |
|  | ACU+RLX | RLX | 1 | 87 | **-1.00 [-1.44, -0.55]** | NA | 4.37 | <0.0001 | fixed |
|  | BT | EDU | 1 | 79 | **-6.13 [-7.20, -5.05]** | NA | 11.17 | <0.00001 | fixed |
|  | BZD | BZD+CBT | 1 | 54 | **2.51 [1.78, 3.23]** | NA | 6.77 | <0.00001 | fixed |
|  | BZD | BZD+EXR | 1 | 220 | **1.14 [0.85, 1.42]** | NA | **7.81** | <0.00001 | fixed |
|  | BZD | CBT | 1 | 54 | **-2.77 [-3.53, -2.01]** | NA | 7.13 | <0.00001 | fixed |
|  | BZD+CBT | CBT | 1 | 54 | **-2.89 [-3.67, -2.11]** | NA | 7.28 | <0.00001 | fixed |
|  | CBT | EDU | 1 | 159 | **-0.61 [-0.95, -0.28]** | NA | 3.57 | 0.0004 | fixed |
|  | CBT | QIG | 1 | 61 | **0.53 [0.02, 1.04]** | NA | 2.03 | 0.04 | fixed |
|  | CBT | WLT | 1 | 39 | **-6.55 [-8.20, -4.89]** | NA | 7.75 | <0.00001 | fixed |
|  | RLX | WLT | 1 | 64 | **-1.04 [-1.56, -0.52]** | NA | 3.89 | <0.0001 | fixed |

**Abbreviations**: ACU, acupuncture; BZD, benzodiazepines; CBT, cognitive behavioral therapy; CI, confidence interval; EDU, sleep education; SMD, standardized mean difference; MTN, melatonin; NA, not applicable; PSQI, the Pittsburgh sleep quality index; QIG, qigong; RLX, relaxation.

**Table S32. PSQI total score (after removing outlier)**

| **Outcome** |  |  | **Studies** | **Participants** | **SMD [95% CI**] | ***I^2^*** | **Z** | **P** | **Model** |
| --- | --- | --- | --- | --- | --- | --- | --- | --- | --- |
| PSQI | ACU | ACU+BZD | 2 | 120 | -0.33 [-0.70, 0.04] | 88 | 1.76 | 0.08 | fixed |
|  | After removing outlier | | 1 | 68 | 0.12 [-0.36, 0.59] | NA | 0.48 | 0.63 | fixed |
|  | ACU | BZD | 9 | 535 | -0.15 [-1.08, 0.79] | 96 | 0.30 | 0.76 | random |
|  | After removing outlier | | 8 | 483 | 0.04 [-0.97, 1.06] | 96 | 0.09 | 0.93 | random |
|  | ACU+BZD | BZD | 3 | 195 | **-1.24 [-1.56, -0.91]** | 95 | 7.40 | <0.00001 | fixed |
|  | After removing outlier | | 2 | 143 | **-1.47 [-1.87, -1.07]** | 97 | 7.19 | <0.00001 | fixed |

**Abbreviations**: ACU, acupuncture; BZD, benzodiazepines; CI, confidence interval; SMD, standardized mean difference; NA, not applicable; PSQI, the Pittsburgh sleep quality index.

**Table S33. Polysomnography data**

| **Outcome** |  |  | **Studies** | **Participants** | **SMD [95% CI]** | ***I^2^*** | **Z** | **P** | **Model** |
| --- | --- | --- | --- | --- | --- | --- | --- | --- | --- |
| (1) SE (%) | BZD | BZD+CBT | 1 | 82 | **-3.51 [-4.22, -2.81]** | NA | 9.83 | <0.00001 | fixed |
|  | BT | EDU | 1 | 79 | 0.42 [-0.03, 0.86] | NA | 1.84 | 0.07 | fixed |
|  | CBT | WLT | 1 | 48 | **0.79 [0.20, 1.38]** | NA | 2.62 | 0.009 | fixed |
|  | ACU+EDU | EDU | 1 | 67 | **6.84 [5.56, 8.13]** | NA | 10.42 | <0.00001 | fixed |
| (2) WASO (min) | BT | EDU | 1 | 79 | **-0.85 [-1.31, -0.38]** | NA | 3.59 | 0.0003 | fixed |
|  | CBT | WLT | 1 | 48 | -0.53 [-1.11, 0.04] | NA | 1.81 | 0.07 | fixed |
| (3) AN | BZD | BZD+CBT | 1 | 82 | **2.02 [1.49, 2.56]** | NA | 7.38 | <0.00001 | fixed |
|  | ACU+EDU | EDU | 1 | 67 | **-2.11 [-2.72, -1.51]** | NA | 6.85 | <0.00001 | fixed |
| (4) SMR (%) | BZD | BZD+CBT | 1 | 82 | **-1.05 [-1.51, -0.59]** | NA | 4.44 | <0.00001 | fixed |
|  | ACU+EDU | EDU | 1 | 67 | **2.38 [1.74, 3.01]** | NA | 7.35 | <0.00001 | fixed |
| (5) SOL (min) | BT | EDU | 1 | 79 | **0.75 [0.29, 1.21]** | NA | 3.22 | 0.001 | fixed |
|  | CBT | WLT | 1 | 48 | -0.39 [-0.96, 0.19] | NA | 1.32 | 0.19 | fixed |
| (6) TST (min) | BT | EDU | 1 | 79 | **-0.90 [-1.36, -0.43]** | NA | 3.78 | 0.0002 | fixed |
|  | CBT | WLT | 1 | 48 | -0.17 [-0.73, 0.40] | NA | 0.57 | 0.57 | fixed |
| (7) AD (min) | CBT | WLT | 1 | 48 | **-0.99 [-1.59, -0.39]** | NA | 3.22 | 0.001 | fixed |
|  | BZD | BZD+CBT | 1 | 82 | **1.65 [1.15, 2.15]** | NA | 6.41 | <0.00001 | fixed |
|  | ACU+EDU | EDU | 1 | 67 | **-2.25 [-2.87, -1.63]** | NA | 7.12 | <0.00001 | fixed |

**Abbreviations**: ACU, acupuncture; AD, awaken duration; AN, awakening number; BT, behavioral treatment; BZD, benzodiazepines; CBT, cognitive behavioral therapy; CI, confidence interval; EDU, sleep education; SMD, standardized mean difference; NA, not applicable; SE, sleep efficiency; SMR, sleep maintenance rate; SOL, sleep onset latency; TST, total sleep time; WASO, wake after sleep onset; WLT, wait-list.

**Table S34. Drop-outs for any reason**

| **Outcome** |  |  | **Studies** | **Participants** | **OR [95% CI]** | ***I^2^*** | **Z** | **P** | **Model** |
| --- | --- | --- | --- | --- | --- | --- | --- | --- | --- |
| All-cause drop-out | ACU | ACU+BZD | 2 | 120 | there was no drop-out case | NA | NA | NA | NA |
|  | ACU | ACU+RLX | 1 | 100 | 2.14 [0.50, 9.07] | NA | 1.03 | 0.30 | fixed |
|  | ACU | BZD | 10 | 625 | 0.80 [0.26, 2.47] | 0 (only three studies were included in this meta-analysis) | 0.39 | 0.70 | fixed |
|  | ACU | MTN | 2 | 124 | there was no drop-out case | NA | NA | NA | NA |
|  | ACU | RLX | 1 | 100 | 1.57 [0.41, 5.93] | NA | 0.66 | 0.51 | fixed |
|  | ACU+BZD | BZD | 3 | 195 | there was no drop-out case | NA | NA | NA | NA |
|  | ACU+CBT | CBT | 1 | 86 | there was no drop-out case | NA | NA | NA | NA |
|  | ACU+EDU | EDU | 1 | 67 | there was no drop-out case | NA | NA | NA | NA |
|  | ACU+RLX | RLX | 1 | 100 | 0.73 [0.16, 3.46] | NA | 0.39 | 0.70 | fixed |
|  | ACU+RLX | SSRI | 1 | 96 | there was no drop-out case | NA | NA | NA | NA |
|  | ATC | BT | 1 | 62 | 1.64 [0.46, 5.88] | NA | 0.76 | 0.45 | fixed |
|  | BT | EDU | 1 | 82 | 7.18 [0.36, 143.50] | NA | 1.29 | 0.20 | fixed |
|  | BZD | BZD+CBT | 3 | 296 | there was no drop-out case | NA | NA | NA | NA |
|  | BZD | BZD+EXR | 1 | 220 | there was no drop-out case | NA | NA | NA | NA |
|  | BZD | CBT | 1 | 54 | there was no drop-out case | NA | NA | NA | NA |
|  | BZD+CBT | CBT | 1 | 54 | there was no drop-out case | NA | NA | NA | NA |
|  | CBT | EDU | 1 | 159 | there was no drop-out case | NA | NA | NA | NA |
|  | CBT | QIG | 1 | 74 | 1.77 [0.52, 6.01] | NA | 0.91 | 0.36 | fixed |
|  | CBT | WLT | 2 | 63 | 1.69 [0.25, 11.42] | NA (only one study was included in this meta-analysis) | 0.54 | 0.59 | fixed |
|  | RLX | WLT | 1 | 64 | 0.48 [0.04, 5.62] | NA | 0.58 | 0.56 | fixed |

**Abbreviations**: ACU, acupuncture; ATC, attention control; BT, behavioral treatment; BZD, benzodiazepines; CBT, cognitive behavioral therapy; CI, confidence interval; EDU, sleep education; EXR, exercise; MTN, melatonin; NA, not applicable; OR, odds ratio; QIG, qigong; RLX, relaxation; SSRI, selective serotonin reuptake inhibitor; WLT, wait-list.

**Table S35. Drop-outs for AEs**

| **Outcome** |  |  | **Studies** | **Participants** | **OR [95% CI]** | ***I^2^*** | **Z** | **P** | **Model** |
| --- | --- | --- | --- | --- | --- | --- | --- | --- | --- |
| AE drop-out | ACU | ACU+BZD | 2 | 120 | there was no drop-out case | NA | NA | NA | NA |
|  | ACU | BZD | 9 | 565 | 1.58 [0.06, 41.03] | NA (only one study was included in this meta-analysis) | 0.27 | 0.78 | fixed |
|  | ACU | MTN | 2 | 124 | there was no drop-out case | NA | NA | NA | NA |
|  | ACU+BZD | BZD | 3 | 195 | there was no drop-out case | NA | NA | NA | NA |
|  | ACU+CBT | CBT | 1 | 86 | there was no drop-out case | NA | NA | NA | NA |
|  | ACU+EDU | EDU | 1 | 67 | there was no drop-out case | NA | NA | NA | NA |
|  | ACU+RLX | SSRI | 1 | 96 | there was no drop-out case | NA | NA | NA | NA |
|  | ATC | CBT | 1 | 62 | there was no drop-out case | NA | NA | NA | NA |
|  | BZD | BZD+CBT | 3 | 298 | there was no drop-out case | NA | NA | NA | NA |
|  | BZD | BZD+EXR | 1 | 220 | there was no drop-out case | NA | NA | NA | NA |
|  | BZD | CBT | 1 | 54 | there was no drop-out case | NA | NA | NA | NA |
|  | BZD+CBT | CBT | 1 | 54 | there was no drop-out case | NA | NA | NA | NA |
|  | CBT | EDU | 2 | 241 | there was no drop-out case | NA | NA | NA | NA |
|  | CBT | QIG | 1 | 74 | there was no drop-out case | NA | NA | NA | NA |
|  | CBT | WLT | 1 | 24 | there was no drop-out case | NA | NA | NA | NA |
|  | RLX | WLT | 1 | 64 | there was no drop-out case | NA | NA | NA | NA |

**Abbreviations**: ACU, acupuncture; AE, adverse event; ATC, attention control; BT, behavioral treatment; BZD, benzodiazepines; CBT, cognitive behavioral therapy; CI, confidence interval; EDU, sleep education; EXR, exercise; MTN, melatonin; NA, not applicable; OR, odds ratio; QIG, qigong; RLX, relaxation; WLT, wait-list.

**Supplementary Material 7. Network funnel plots**

**Figure S26. PSQI total score**

**
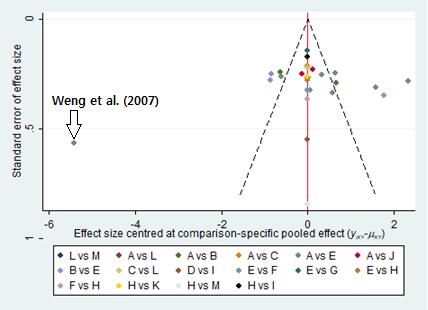
**

**Note**. A=acupuncture, B=acupuncture combined with benzodiazepines, C=acupuncture combined with relaxation, D=benzodiazepines, E=benzodiazepines combined with CBT, F=CBT, G=sleep education, H=melatonin, I=qigong, J=relaxation, K=wait-list.

**Figure S27. Drop-outs for any reason**

**
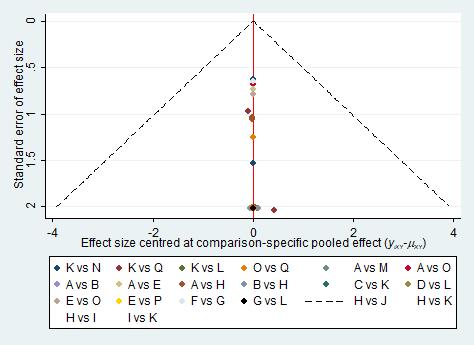
**

**Note**. A=acupuncture, B=acupuncture combined with benzodiazepines, C=acupuncture combined with CBT, D=acupuncture combined with relaxation, E=attention control, F=benzodiazepines, G=benzodiazepines combined with CBT, H=CBT, I=sleep education, J=melatonin, K=qigong, L=relaxation, M=wait-list.

**Supplementary Material 8. Quality of evidence**

**Table S36. PSQI total score**

| **Comparison** | | **Direct evidence** | **Indirect evidence** | **Network meta-analysis** |
| --- | --- | --- | --- | --- |
| ACU | ACU+BZD | Very low | Very low | Very low |
| ACU | ACU+RLX | Moderate | Moderate | Moderate |
| ACU | BT | - | Low | Low |
| ACU | BZD | Very low | Very low | Very low |
| ACU | BZD+CBT | - | Low | Low |
| ACU | BZD+EXR | - | Low | Low |
| ACU | CBT | - | Low | Low |
| ACU | EDU | - | Low | Low |
| ACU | MTN | Low | - | Low |
| ACU | QIG | - | Low | Low |
| ACU | RLX | Moderate | Moderate | Moderate |
| ACU | WLT | - | Moderate | Moderate |
| ACU+BZD | ACU+RLX | - | Low | Low |
| ACU+BZD | BT | - | Low | Low |
| ACU+BZD | BZD | Very low | Very low | Very low |
| ACU+BZD | BZD+CBT | - | Low | Low |
| ACU+BZD | BZD+EXR | - | Low | Low |
| ACU+BZD | CBT | - | Low | Low |
| ACU+BZD | EDU | - | Low | Low |
| ACU+BZD | MTN | - | Low | Low |
| ACU+BZD | QIG | - | Low | Low |
| ACU+BZD | RLX | - | Low | Low |
| ACU+BZD | WLT | - | Low | Low |
| ACU+RLX | BT | - | Low | Low |
| ACU+RLX | BZD | - | Low | Low |
| ACU+RLX | BZD+CBT | - | Low | Low |
| ACU+RLX | BZD+EXR | - | Low | Low |
| ACU+RLX | CBT | - | Low | Low |
| ACU+RLX | EDU | - | Low | Low |
| ACU+RLX | MTN | - | Low | Low |
| ACU+RLX | QIG | - | Low | Low |
| ACU+RLX | RLX | Moderate | Moderate | Moderate |
| ACU+RLX | WLT | - | Moderate | Moderate |
| BT | BZD | - | Low | Low |
| BT | BZD+CBT | - | Low | Low |
| BT | BZD+EXR | - | Low | Low |
| BT | CBT | - | Low | Low |
| BT | EDU | Moderate | - | Moderate |
| BT | MTN | - | Low | Low |
| BT | QIG | - | Low | Low |
| BT | RLX | - | Moderate | Moderate |
| BT | WLT | - | Moderate | Moderate |
| BZD | BZD+CBT | Moderate | Moderate | Moderate |
| BZD | BZD+EXR | Moderate | - | Moderate |
| BZD | CBT | Moderate | Moderate | Moderate |
| BZD | EDU | - | Low | Low |
| BZD | MTN | - | Low | Low |
| BZD | QIG | - | Low | Low |
| BZD | RLX | - | Low | Low |
| BZD | WLT | - | Moderate | Moderate |
| BZD+CBT | BZD+EXR | - | Low | Low |
| BZD+CBT | CBT | Moderate | Moderate | Moderate |
| BZD+CBT | EDU | - | Low | Low |
| BZD+CBT | MTN | - | Low | Low |
| BZD+CBT | QIG | - | Low | Low |
| BZD+CBT | RLX | - | Low | Low |
| BZD+CBT | WLT | - | Moderate | Moderate |
| BZD+EXR | CBT | - | Low | Low |
| BZD+EXR | EDU | - | Low | Low |
| BZD+EXR | MTN | - | Low | Low |
| BZD+EXR | QIG | - | Low | Low |
| BZD+EXR | RLX | - | Low | Low |
| BZD+EXR | WLT | - | Low | Low |
| CBT | EDU | High | - | High |
| CBT | MTN | - | Low | Low |
| CBT | QIG | Moderate | - | Moderate |
| CBT | RLX | - | Low | Low |
| CBT | WLT | Moderate | - | Moderate |
| EDU | MTN | - | Low | Low |
| EDU | QIG | - | Low | Low |
| EDU | RLX | - | Low | Low |
| EDU | WLT | - | Low | Low |
| MTN | QIG | - | Low | Low |
| MTN | RLX | - | Low | Low |
| MTN | WLT | - | Low | Low |
| QIG | RLX | - | Low | Low |
| QIG | WLT | - | Low | Low |
| RLX | WLT | Moderate | - | Moderate |

**Abbreviations.** ACU, acupuncture; BT, behavioral treatment; BZD, benzodiazepines; CBT, cognitive behavioral therapy; EDU, sleep education; EXR, exercise; MTN, melatonin; QIG, qigong; RLX, relaxation.

Table S37. Drop-outs for any reasons

| **Comparison** | | **Direct evidence** | **Indirect evidence** | **Network meta-analysis** |
| --- | --- | --- | --- | --- |
| ACU | ACU+BZD | Moderate | Low | Moderate |
| ACU | ACU+CBT | - | Low | Low |
| ACU | ACU+EDU | - | Low | Low |
| ACU | ACU+RLX | Low | Low | Low |
| ACU | ATC | - | Low | Low |
| ACU | BT | - | Low | Low |
| ACU | BZD | Low | Moderate | Moderate |
| ACU | BZD+CBT | - | Low | Low |
| ACU | BZD+EXR | - | Low | Low |
| ACU | CBT | - | Low | Low |
| ACU | EDU | - | Low | Low |
| ACU | MTN | Moderate | - | Moderate |
| ACU | QIG | - | Low | Low |
| ACU | RLX | Low | Low | Low |
| ACU | SSRI | - | Low | Low |
| ACU | WLT | - | Low | Low |
| ACU+BZD | ACU+CBT | - | Low | Low |
| ACU+BZD | ACU+EDU | - | Low | Low |
| ACU+BZD | ACU+RLX | - | Low | Low |
| ACU+BZD | ATC | - | Low | Low |
| ACU+BZD | BT | - | Low | Low |
| ACU+BZD | BZD | Moderate | Low | Moderate |
| ACU+BZD | BZD+CBT | - | Low | Low |
| ACU+BZD | BZD+EXR | - | Low | Low |
| ACU+BZD | CBT | - | Low | Low |
| ACU+BZD | EDU | - | Low | Low |
| ACU+BZD | MTN | - | Low | Low |
| ACU+BZD | QIG | - | Low | Low |
| ACU+BZD | RLX | - | Low | Low |
| ACU+BZD | SSRI | - | Low | Low |
| ACU+BZD | WLT | - | Low | Low |
| ACU+CBT | ACU+EDU | - | Low | Low |
| ACU+CBT | ACU+RLX | - | Low | Low |
| ACU+CBT | ATC | - | Low | Low |
| ACU+CBT | BT | - | Low | Low |
| ACU+CBT | BZD | - | Low | Low |
| ACU+CBT | BZD+CBT | - | Low | Low |
| ACU+CBT | BZD+EXR | - | Low | Low |
| ACU+CBT | CBT | Moderate | - | Moderate |
| ACU+CBT | EDU | - | Low | Low |
| ACU+CBT | MTN | - | Low | Low |
| ACU+CBT | QIG | - | Low | Low |
| ACU+CBT | RLX | - | Low | Low |
| ACU+CBT | SSRI | - | Low | Low |
| ACU+CBT | WLT | - | Low | Low |
| ACU+EDU | ACU+RLX | - | Low | Low |
| ACU+EDU | ATC | - | Low | Low |
| ACU+EDU | BT | - | Low | Low |
| ACU+EDU | BZD | - | Low | Low |
| ACU+EDU | BZD+CBT | - | Low | Low |
| ACU+EDU | BZD+EXR | - | Low | Low |
| ACU+EDU | CBT | - | Low | Low |
| ACU+EDU | EDU | Moderate | - | Moderate |
| ACU+EDU | MTN | - | Low | Low |
| ACU+EDU | QIG | - | Low | Low |
| ACU+EDU | RLX | - | Low | Low |
| ACU+EDU | SSRI | - | Low | Low |
| ACU+EDU | WLT | - | Low | Low |
| ACU+RLX | ATC | - | Low | Low |
| ACU+RLX | BT | - | Low | Low |
| ACU+RLX | BZD | - | Low | Low |
| ACU+RLX | BZD+CBT | - | Low | Low |
| ACU+RLX | BZD+EXR | - | Low | Low |
| ACU+RLX | CBT | - | Low | Low |
| ACU+RLX | EDU | - | Low | Low |
| ACU+RLX | MTN | - | Low | Low |
| ACU+RLX | QIG | - | Low | Low |
| ACU+RLX | RLX | Low | Low | Low |
| ACU+RLX | SSRI | Moderate | - | Moderate |
| ACU+RLX | WLT | - | Low | Low |
| ATC | BT | Low | - | Low |
| ATC | BZD | - | Low | Low |
| ATC | BZD+CBT | - | Low | Low |
| ATC | BZD+EXR | - | Low | Low |
| ATC | CBT | - | Low | Low |
| ATC | EDU | - | Low | Low |
| ATC | MTN | - | Low | Low |
| ATC | QIG | - | Low | Low |
| ATC | RLX | - | Low | Low |
| ATC | SSRI | - | Low | Low |
| ATC | WLT | - | Low | Low |
| BT | BZD | - | Low | Low |
| BT | BZD+CBT | - | Low | Low |
| BT | BZD+EXR | - | Low | Low |
| BT | CBT | - | Low | Low |
| BT | EDU | Low | - | Low |
| BT | MTN | - | Low | Low |
| BT | QIG | - | Low | Low |
| BT | RLX | - | Low | Low |
| BT | SSRI | - | Low | Low |
| BT | WLT | - | Low | Low |
| BZD | BZD+CBT | Moderate | Moderate | Moderate |
| BZD | BZD+EXR | Moderate | - | Moderate |
| BZD | CBT | Moderate | Moderate | Moderate |
| BZD | EDU | - | Low | Low |
| BZD | MTN | - | Low | Low |
| BZD | QIG | - | Low | Low |
| BZD | RLX | - | Low | Low |
| BZD | SSRI | - | Low | Low |
| BZD | WLT | - | Low | Low |
| BZD+CBT | BZD+EXR | - | Low | Low |
| BZD+CBT | CBT | Moderate | Moderate | Moderate |
| BZD+CBT | EDU | - | Low | Low |
| BZD+CBT | MTN | - | Low | Low |
| BZD+CBT | QIG | - | Low | Low |
| BZD+CBT | RLX | - | Low | Low |
| BZD+CBT | SSRI | - | Low | Low |
| BZD+CBT | WLT | - | Low | Low |
| BZD+EXR | CBT | - | Low | Low |
| BZD+EXR | EDU | - | Low | Low |
| BZD+EXR | MTN | - | Low | Low |
| BZD+EXR | QIG | - | Low | Low |
| BZD+EXR | RLX | - | Low | Low |
| BZD+EXR | SSRI | - | Low | Low |
| BZD+EXR | WLT | - | Low | Low |
| CBT | EDU | Moderate | - | Moderate |
| CBT | MTN | - | Low | Low |
| CBT | QIG | Low | - | Low |
| CBT | RLX | - | Low | Low |
| CBT | SSRI | - | Low | Low |
| CBT | WLT | Low | - | Low |
| EDU | MTN | - | Low | Low |
| EDU | QIG | - | Low | Low |
| EDU | RLX | - | Low | Low |
| EDU | SSRI | - | Low | Low |
| EDU | WLT | - | Low | Low |
| MTN | QIG | - | Low | Low |
| MTN | RLX | - | Low | Low |
| MTN | SSRI | - | Low | Low |
| MTN | WLT | - | Low | Low |
| QIG | RLX | - | Low | Low |
| QIG | SSRI | - | Low | Low |
| QIG | WLT | - | Low | Low |
| RLX | SSRI | - | Low | Low |
| RLX | WLT | Low | - | Low |
| SSRI | WLT | - | Low | Low |

**Abbreviations**: ACU, acupuncture; ATC, attention control; BT, behavioral treatment; BZD, benzodiazepines; CBT, cognitive behavioral therapy; EDU, sleep education; EXR, exercise; MTN, melatonin; QIG, qigong; RLX, relaxation; SSRI, selective serotonin reuptake inhibitor; WLT, wait-list.
